# Multiomics Analyses Provide New Insight into Genetic Variation of Reproductive Adaptability in Tibetan Sheep

**DOI:** 10.1093/molbev/msae058

**Published:** 2024-03-29

**Authors:** Buying Han, Dehong Tian, Xue Li, Sijia Liu, Fei Tian, Dehui Liu, Song Wang, Kai Zhao

**Affiliations:** Key Laboratory of Adaptation and Evolution of Plateau Biota, Northwest Institute of Plateau Biology, Chinese Academy of Sciences, Xining, China; University of Chinese Academy of Sciences, Beijing, China; Qinghai Provincial Key Laboratory of Animal Ecological Genomics, Northwest Institute of Plateau Biology, Chinese Academy of Sciences, Xining, China; Key Laboratory of Adaptation and Evolution of Plateau Biota, Northwest Institute of Plateau Biology, Chinese Academy of Sciences, Xining, China; Qinghai Provincial Key Laboratory of Animal Ecological Genomics, Northwest Institute of Plateau Biology, Chinese Academy of Sciences, Xining, China; Key Laboratory of Adaptation and Evolution of Plateau Biota, Northwest Institute of Plateau Biology, Chinese Academy of Sciences, Xining, China; Qinghai Provincial Key Laboratory of Animal Ecological Genomics, Northwest Institute of Plateau Biology, Chinese Academy of Sciences, Xining, China; Key Laboratory of Adaptation and Evolution of Plateau Biota, Northwest Institute of Plateau Biology, Chinese Academy of Sciences, Xining, China; Qinghai Provincial Key Laboratory of Animal Ecological Genomics, Northwest Institute of Plateau Biology, Chinese Academy of Sciences, Xining, China; Key Laboratory of Adaptation and Evolution of Plateau Biota, Northwest Institute of Plateau Biology, Chinese Academy of Sciences, Xining, China; Qinghai Provincial Key Laboratory of Animal Ecological Genomics, Northwest Institute of Plateau Biology, Chinese Academy of Sciences, Xining, China; Key Laboratory of Adaptation and Evolution of Plateau Biota, Northwest Institute of Plateau Biology, Chinese Academy of Sciences, Xining, China; University of Chinese Academy of Sciences, Beijing, China; Qinghai Provincial Key Laboratory of Animal Ecological Genomics, Northwest Institute of Plateau Biology, Chinese Academy of Sciences, Xining, China; Key Laboratory of Adaptation and Evolution of Plateau Biota, Northwest Institute of Plateau Biology, Chinese Academy of Sciences, Xining, China; University of Chinese Academy of Sciences, Beijing, China; Qinghai Provincial Key Laboratory of Animal Ecological Genomics, Northwest Institute of Plateau Biology, Chinese Academy of Sciences, Xining, China; Key Laboratory of Adaptation and Evolution of Plateau Biota, Northwest Institute of Plateau Biology, Chinese Academy of Sciences, Xining, China; Qinghai Provincial Key Laboratory of Animal Ecological Genomics, Northwest Institute of Plateau Biology, Chinese Academy of Sciences, Xining, China

**Keywords:** Tibetan sheep, adaptation, reproduction, multiomics, GWAS

## Abstract

Domestication and artificial selection during production-oriented breeding have greatly shaped the level of genomic variability in sheep. However, the genetic variation associated with increased reproduction remains elusive. Here, two groups of samples from consecutively monotocous and polytocous sheep were collected for genome-wide association, transcriptomic, proteomic, and metabolomic analyses to explore the genetic variation in fecundity in Tibetan sheep. Genome-wide association study revealed strong associations between *BMPR1B* (p.Q249R) and litter size, as well as between *PAPPA* and lambing interval; these findings were validated in 1,130 individuals. Furthermore, we constructed the first single-cell atlas of Tibetan sheep ovary tissues and identified a specific mural granulosa cell subtype with *PAPPA*-specific expression and differential expression of *BMPR1B* between the two groups. Bulk RNA-seq indicated that *BMPR1B* and *PAPPA* expressions were similar between the two groups of sheep. 3D protein structure prediction and coimmunoprecipitation analysis indicated that mutation and mutually exclusive exons of *BMPR1B* are the main mechanisms for prolific Tibetan sheep. We propose that *PAPPA* is a key gene for stimulating ovarian follicular growth and development, and steroidogenesis. Our work reveals the genetic variation in reproductive performance in Tibetan sheep, providing insights and valuable genetic resources for the discovery of genes and regulatory mechanisms that improve reproductive success.

## Introduction

Tibetan sheep (*Ovis aries*), one of the three major primitive sheep breeds in China, have lived on the Qinghai–Tibet Plateau (QTP) for thousands of years and adapted to the harsh environment (Yang et al. 2016; Chen, Sun, et al. 2021). It is an excellent breed formed through long-term natural selection and artificial breeding for its superior characteristics of drought, cold, rough feeding, and strong disease resistance, adaptation to high altitude, strong foraging ability and physique, and strong genetic adaptation (Zhang et al. 2016). In addition, it is extremely important for the economic development of pastoral areas (Hu et al. 2019). However, Tibetan sheep have the production disadvantages of late sexual maturity and low reproductive performance and typically give birth to one lamb a year. These characteristics restrict the efficient and sustainable development of Tibetan sheep breeding in pastoral areas.

The successful reproduction is a crucial part for adaptation to high altitude, particularly for domestic animals that face both natural and artificial selection. Tibetan sheep is an excellent model for providing insights into genetic mechanisms that characterize adaptive responses of livestock to extreme environments (Zhang et al. 2016). Litter size and lambing interval are two important reproductive traits that affect the production yield of sheep (Pasandideh et al. 2020). Litter size is the most heavily weighted reproductive trait among the three economically important traits of sheep (lambing, meat, and fur; Abdoli et al. 2016). Previous studies have emphasized the importance of bone morphogenetic protein receptor 1B (*BMPR1B*; Mulsant et al. 2001) on the genetics of prolificacy in different sheep breeds including Booroola Merino sheep (Montgomery et al. 1993), small-tailed Han sheep (Chu et al. 2007), Hu sheep (Lv et al. 2022), Mongolian sheep (Gao et al. 2021), and Garole sheep (Polley et al. 2010). A substitution mutation in *BMPR1B* has an additive effect on ovulation rate and litter size (Hua and Yang 2009). Pregnancy-associated plasma protein-A (*PAPPA*) plays a crucial role in various reproductive processes, such as the stimulation of granulosa cell (GC) proliferation and steroidogenesis, follicular development, and ovulation (Mazerbourg et al. 2001). Lambing interval is also an economically critical reproductive trait that directly affects reproductive efficiency and profitability in animal husbandry. Litter size and lambing interval are extremely complex traits regulated by genetic and epigenetic modifications, and hormonal factors. Researchers have investigated the mechanisms of prolificacy trait formation in sheep using physiology, reproduction, endocrinology, genetic markers, candidate gene, and single histology approaches (Butler et al. 1981; Abdoli et al. 2016; Cao et al. 2016; Gholizadeh and Esmaeili-Fard 2022). However, the lack of comprehensive insight into the genetic mechanisms underlying critical reproductive traits, particularly from a multiomics perspective, has hindered the effective advancement of sheep breeding.

To explore the molecular basis of reproductive characteristics in Tibetan sheep, we investigated reproduction in Tibetan sheep, which usually have one lamb a year (common), but also twins and triplets within the same population (uncommon). Notably, we found that ewes with high fecundity have shorter lambing interval, implying that they could have three births in 2 yr. Here, we combined genome–wide association study (GWAS), transcriptome, proteome, and metabolome data to explore the genetic mechanisms underlying the high-fertility (HF) and low-fertility (LF) phenotypes of Tibetan sheep. Furthermore, ovarian single-cell transcriptomic analysis was used to determine the genetic basis of unique traits that may contribute to reproductive performance and thereby improve the production performance of Tibetan sheep. Overall, this study provides a valuable genetic resource for future research, and the scientific use of these data will contribute to the improvement of sheep fertility and the sustainable development of animal husbandry. More broadly, our work will contribute to the planning of appropriate breeding programs under various future climate change scenarios.

## Results

### Litter Size and Lambing Interval–Related Single Nucleotide Polymorphisms

**Fig. 1.**
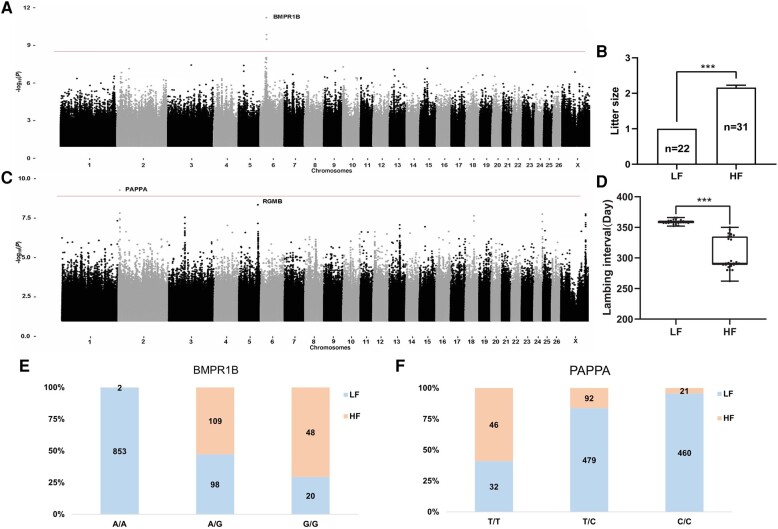
Whole-genome sequencing found that *BMPR1B* and *PAPPA* were associated with reproductive phenotypes in Tibetan sheep. a) Manhattan plot showing the significant association between the mutation in *BMPR1B* with litter size differences between HF and LF groups. The significance threshold *P*–value [−log_10_(*P*) = 8.87] is denoted by the line. b) Statistics of litter size in the two groups of Tibetan sheep. HF and LF indicate high-fertility and low-fertility groups. The bars represent mean ± SD. A two-tailed *t* test was used (***P < 0.001). c) Manhattan plot showing the significant association between a SNP in *PAPPA* on chromosome 2 with the lambing interval phenotype. The red horizontal line corresponds to the genome-wide significance threshold [−log_10_(*P*) = 8.87]. d) Statistics of lambing interval in the two groups of Tibetan sheep (*P < 0.05; **P < 0.01; ***P < 0.001). e) Statistics on the proportion of *BMPR1B* genotypes in a large population with different reproductive phenotypes. The orange and blue histograms represent sheep with high and low fertility in the large population (*n* = 1,130). f) Statistics on the proportion of *PAPPA* genotypes in a large population (*n* = 1,130).

To explore the genetics of highly prolific Tibetan sheep, we performed a whole-genome sequencing using 31 HF and 22 LF Tibetan sheep that had been lambing continuously for more than 3 yr (supplementary table S1, Supplementary Material online). In total, 1,442.37 Gb of sequence data was generated, resulting in averages over the 53 samples for depth (10.35×) and coverage (99.35%) of the *O. aries* reference genome (ARS-UI_Ramb_v2.0, GCF_016772045.1; Davenport et al. 2022; supplementary table S2, Supplementary Material online). After quality control, 37,101,958 single nucleotide polymorphisms (SNPs) remained for GWAS analysis of litter size and lambing interval phenotypes. We applied GWAS using Fisher's exact test with a Bonferroni-corrected *P* < 0.05 threshold (Tang et al. 2023). Significant SNPs were identified on chromosome 6, which contained the well-known reproductive gene *BMPR1B* (Souza et al. 2001) (−log_10_*P* = 8.87) and showed a strong association with the litter size phenotype (Fig. 1a and b). A significant SNP was also identified on chromosome 2, located on the *PAPPA* (−log_10_*P* = 8.87) and showed a strong association with lambing interval phenotype (Fig. 1c and d; supplementary table S3, Supplementary Material online). One variant (Chr6: 30,050,621, c.746A > G, p.Gln249Arg) was predicted to have a missense impact on proteins. The p.Gln249Arg missense mutation occurred specifically in the HF Tibetan sheep and was located within protein-coding exon 8 of *BMPR1B*. The *PAPPA* intron variant (Chr2: 7,400,929, c.3,618–1,333T > C) occurred specifically in LF ewes, which had longer lambing interval than HF ewes. These results imply that *PAPPA* may regulate the duration of the lambing interval.

To confirm the association between genetic architecture and litter size, we examined the genotypic data of the mutant loci. Genotyping revealed that 19 of the 22 LF individuals were homozygous for the A/A allele, whereas HF individuals were either heterozygous (A/G, *n* = 24) or homozygous (G/G, *n* = 7; supplementary fig. S1, Supplementary Material online). Likewise, examination of genotypic data of the mutant loci revealed an association between genetic architecture and lambing interval. Genotyping revealed that 16 of the 22 LF individuals were homozygous for the C/C allele, whereas HF individuals were mainly wild type (T/T, *n* = 14; supplementary fig. S2, Supplementary Material online). Subsequently, the KASP (Liang et al. 2021) method was used to examine the proportion of mutations in *BMPR1B* and *PAPPA* in a large population (*n* = 1,130, supplementary table S4, Supplementary Material online). These results showed that *BMPR1B* exhibited a high proportion of heterozygous (A/G, *n* = 109) and homozygous (G/G, *n* = 48) mutations in the 159 HF individuals (Fig. 1e). There were 92 heterozygous (T/C) and 46 homozygous (T/T) mutations in *PAPPA* in the 159 HF individuals (Fig. 1f). The HF individuals had shorter lambing interval than LF individuals. The results suggested that HF ewes had the ability to produce three births in 2 yr.

### Identification of Phenotypes of Dominant Follicles

**Fig. 2.**
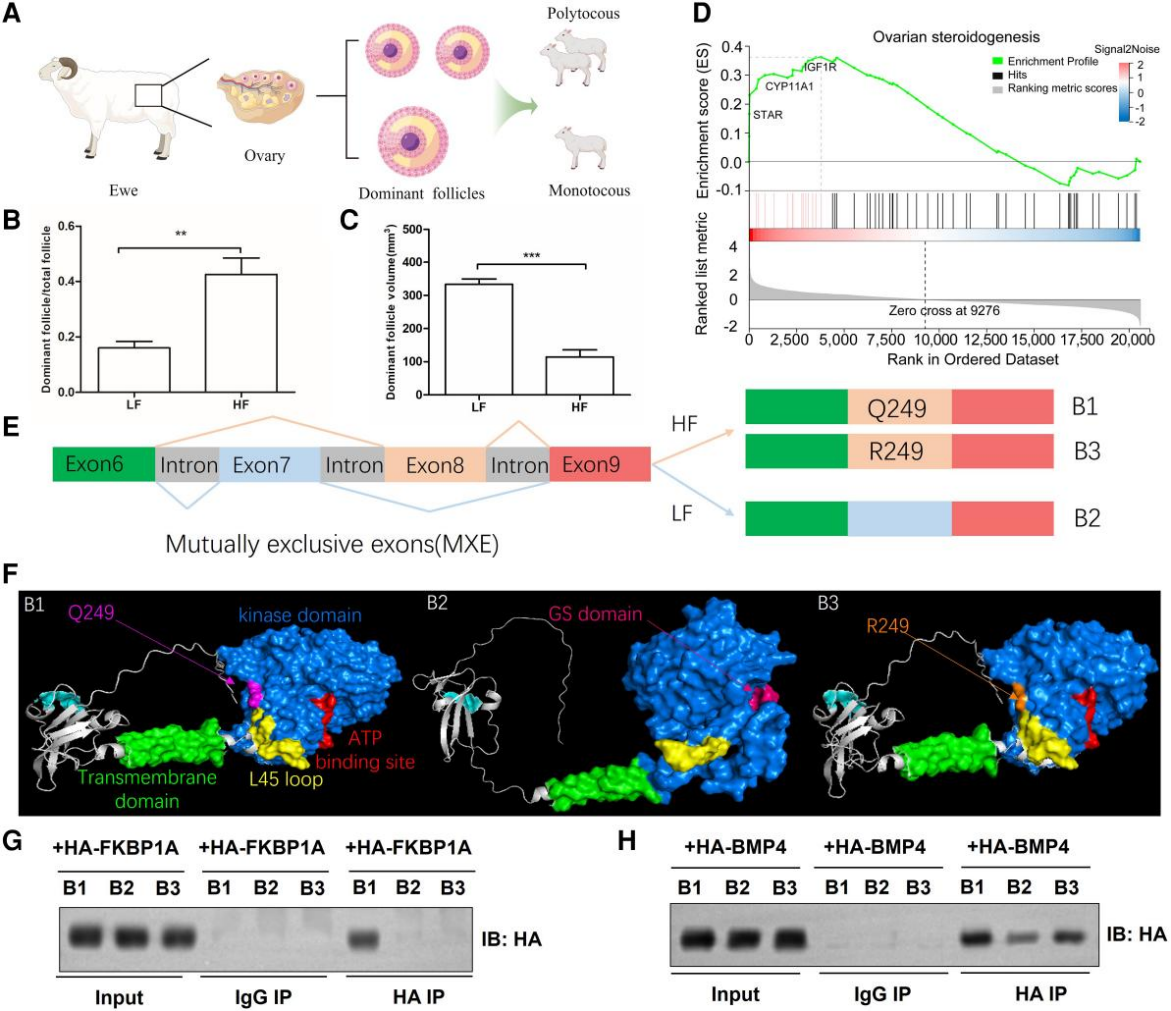
AS events and Co-IP analysis revealed that *BMPR1B* was associated with high production in Tibetan sheep. a) Schematic representation of Tibetan sheep ovarian tissue and follicular fluid preparation for multiomics analysis. b) Statistics of dominant follicle/total follicle numbers in HF and LF groups of Tibetan sheep (**P < 0.01). c) Statistics of the dominant follicle volume in the two groups of Tibetan sheep (***P < 0.001). d) GSEA results of the KEGG pathway “Ovarian steroidogenesis” is shown. e) Differences in MXE between the HF and LF groups. f) The 3D structure, predicted by AlphaFold2, of proteins B1, B2, and B3 encoded by variable splicing, with cysteine residues in the extracellular domain, the GS motif, transmembrane domain, loop 45, and kinase domain. Protein B3 included a mutation from Q to R on exon 8 of protein B1. g) Co-IP assays show that FKBP1A binds to B1 in Tibetan sheep. h) Co-IP assays show that sheep BMP4 complexes with B1, B2, and B3.

To further investigate phenotypic differences, three samples were randomly selected from each of the HF and LF groups for ovarian phenotype measurements. This study investigated the size and quantity of dominant ovarian follicles in ewes with varying litter sizes (Fig. 2a to c; supplementary table S5, Supplementary Material online). The results indicated that the number of dominant follicles exhibited a consistent relationship with litter size in both groups. There were no significant differences in ovarian weight/body weight ratios (supplementary fig. S3, Supplementary Material online), suggesting that ovary and body weights did not interfere with analysis results. Dominant follicle numbers were significantly greater in HF ewes (Fig. 2b). Notably, HF ewes had smaller dominant follicles, which ultimately could lead to an increased litter size (Fig. 2a and c). These findings indicated that differences in the number and size of dominant ovarian follicles, as found in a previous study of dominant follicles (Spicer 2004), may be responsible for the marked litter size difference in Tibetan sheep. Individuals with confirmed phenotypes were used for the following transcriptomic, proteomic, and metabolomic analyses to explore the genetic mechanisms of fecundity in Tibetan sheep.

### Analysis of Transcriptomics and Alternative Splicing Events

To determine whether differences in ovarian transcript levels contributed to the variation in lambing numbers in two groups, RNA sequencing (RNA-seq) was performed using ovarian samples from six estrus monotocous and polytocous ewes in three consecutive lambing seasons (the high and low reproductive groups with ≥2 lambs and one lamb per season, *n* = 3, respectively; supplementary table S5, Supplementary Material online). This analysis indicated that 86 genes in total were differentially expressed (supplementary table S6, Supplementary Material online), such as the key steroid hormone biosynthesis gene, steroidogenic acute regulatory protein (*STAR*; log_2_fold-change = 2.33; *Q* value = 3.65 × 10^−4^; supplementary fig. S4, Supplementary Material online). *STAR* plays a key role in steroid hormone synthesis by enhancing the metabolism of cholesterol into pregnenolone, and steroid production is essential for follicular development and ovulation (Men et al. 2017). Based on gene set enrichment analysis (GSEA), the most significantly enriched differentially expressed genes (DEGs), such as *STAR*, *IGF1R*, and *CYP11A1*, were involved in ovarian steroidogenesis (Fig. 2d). The significant difference in *STAR* expression further highlighted the crucial role of follicular development in determining the number and size of dominant follicles.

There were no significant differences in ovarian *BMPR1B* expression between the HF and LF groups. Five basic types of alternative splicing (AS) events were found, including skipped exon (SE), alternative 5′ splice site (A5SS), alternative 3′ splice site (A3SS), mutually exclusive exons (MXEs), and retained intron (RI). The SE was the most prevalent AS event, followed by A5SS and A3SS (supplementary table S7, Supplementary Material online). Analysis of AS events identified differential MXE of *BMPR1B* between monotocous and polytocous sheep (supplementary table S8, Supplementary Material online). B1 and B3 isoforms were identified in the HF group, while B2 isoforms were identified in the LF group (supplementary fig. S5A, Supplementary Material online). The predicted protein B1 was encoded by exons 6, 8, and 9; protein B2 was encoded by exons 6, 7, and 9; and B3 contained a glutamine (Gln) to arginine (Arg) mutation in exon 8 (Fig. 2e). The main domains of B1 and B3 included the extracellular domain, transmembrane domain, kinase domain, ATP-binding site, and loop 45 (Fig. 2f). Interestingly, B2 has a glycine–serine (GS) motif and no ATP-binding site (Fig. 2f; supplementary fig. S5B, Supplementary Material online). AS events and protein 3D structure prediction analyses may provide valuable insights into mutations and MXEs of the *BMPR1B* gene in prolific Tibetan sheep.

It has been found that BMPR1B activity is regulated by FKBP prolyl isomerase 1A (FKBP1A), which plays a key role in the control of BMPR1B (Aghdasi et al. 2001). FKBP1A is a small-molecule inhibitory protein that specifically binds to the GS domain of BMPR1B (Galat 2013). The bone morphogenetic protein 4 (BMP4) is a member of the bone morphogenetic protein family and one of the ligands for BMPR1B (Rajesh et al. 2018). Binding of BMP4 ligands to the BMPR1B initiates a phosphorylation cascade that subsequently phosphorylates receptor-activated SMAD proteins and regulates downstream gene expression. To study the interactions between BMPR1B isoforms (B1, B2, and B3) and FKBP1A or BMP4, recombinant proteins were expressed in a mammalian expression system. Coimmunoprecipitation (Co-IP) and immunoblotting (IB) showed that B1 interacted with FKBP1A, but B2 and B3 did not bind to FKBP1A (Fig. 2g). Further analysis of the binding of proteins from different AS types to BMP4 ligand showed that B2 bound to the BMP4 ligand weaklier than B1 and B3 (Fig. 2h). These results suggested that mutations and AS of *BMPR1B* may play an important role in increasing litter size in Tibetan sheep.

### Single-Cell Clustering and Cell Type Identification

**Fig. 3.**
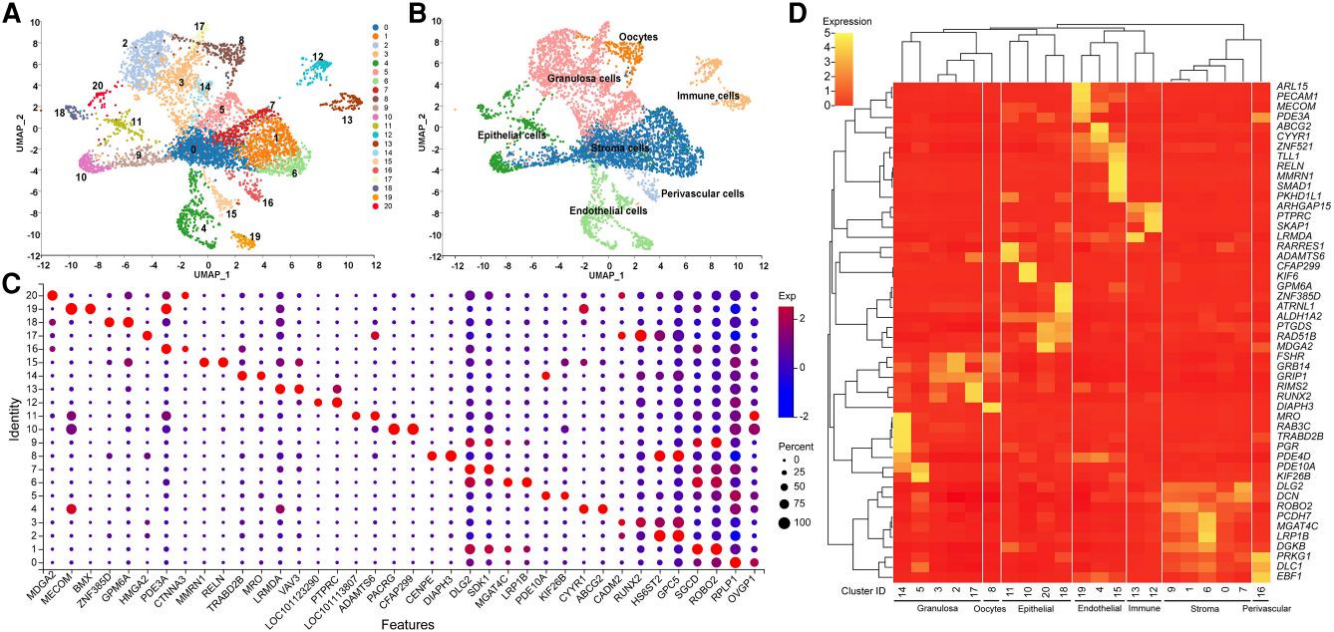
Clustering analysis of various cells in the ovaries of Tibetan sheep. a) UMAP cluster map revealing 21 (CL0 to CL20) specific clusters representing the major ovarian cell types. b) UMAP cluster map showing the seven major ovarian cell types in Tibetan sheep. c) Dot plot showing distinct expression patterns of the selected features genes for each cell type. The expression level of each gene is indicated by a color gradient from low to high. The percentage of cells expressing a specific gene is indicated by the size of a dot. d) Heatmap and hierarchical clustering based on the expression of the top 50 most variable genes.

To investigate the role of *BMPR1B* and *PAPPA* in ovarian development at single-cell resolution, a single-cell atlas was constructed for Tibetan sheep ovaries (Fig. 3a). The percentages of the “nFeature” (mRNA expression), “nCount” (mRNA read counts), and mitochondria (supplementary fig. S6, Supplementary Material online) were successfully applied to quality control. After the quality control filters, 111,548 single cells expressing 17,786 genes were retained for downstream analyses (supplementary table S9, Supplementary Material online). Then, cell clusters were visualized through the nonlinear dimensionality reduction algorithm “Uniform Manifold Approximation and Projection” (UMAP) in a 2D plot. The ovarian cells were clustered into 21 cell clusters (Fig. 3a). Subsequently, the clusters were manually annotated as distinct cell types according to “SCSA” annotation results (Cao et al. 2020), CellMarker data set, previous relevant references, and biological functions of characteristic genes (supplementary table S10, Supplementary Material online). Finally, cell clusters were annotated into seven cell types, including stromal cells (44.14%), GCs (28.73%), oocytes (3.77%), endothelial cells (8.73%), epithelial cells (7.81%), immune cells (4.85%), and perivascular cells (1.96%; Fig. 3b). The expression levels and percentages of genes for features across the different clusters are visualized in a dot matrix plot shown in Fig. 3c. To further define the identity of these cell types in the clusters (CL), hierarchical clustering was generated using the 50 most variably expressed gene means for each cluster, which distinguished seven major cell types: stromal cells (CL0, CL1, CL6, CL7, and CL9), GC (CL2, CL3, CL5, C14, and CL17), oocytes (CL8), endothelial cells (CL4, CL15, and CL19), epithelial cells (CL10, CL11, CL18, and CL20), immune cells (CL12 and CL13), and perivascular cells (CL16; Fig. 3d). The proportions of cell clusters differed between the HF and LF groups. The proportions of CL0 and CL10 in HF were lower than those in LF, whereas CL8 and CL9 were higher than those in LF (supplementary fig. S7, Supplementary Material online). Specific gene expression indicated that the cell type–specific genes *DCN*, *FSHR*, *DIAPH3*, *TLL1*, *ALDH1A2*, *PTPRC*, and *EBF1* can be considered marker genes for stromal cells, GC, oocytes, endothelial cells, epithelial cells, immune cells, and perivascular cells, respectively (supplementary fig. S8, Supplementary Material online). Most cells were broadly categorized as stromal cells, and there was no significant difference in gene expression between stromal cells, but rather high expression levels of the marker genes *DCN*, *PDGFRA*, *COL1A1*, and *COL6A1* (Wagner et al. 2020). A cluster was identified relative to GC by the expression of classical markers *FSH*, *CYP11A1*, and *INHBA* (Zhang et al. 2018). The expression levels of oocyte-related genes *CENPE*, *MIS18BP1*, and *TOP2A* were higher in oocytes than in other cell types, except *DIAPH3* (supplementary table S11, Supplementary Material online), consistent with results previously reported for oocyte gene expression in yaks in the Tibetan Plateau (Pei et al. 2023).

### Cellular Signatures of Different GC Populations in the Ovaries of Tibetan Sheep

**Fig. 4.**
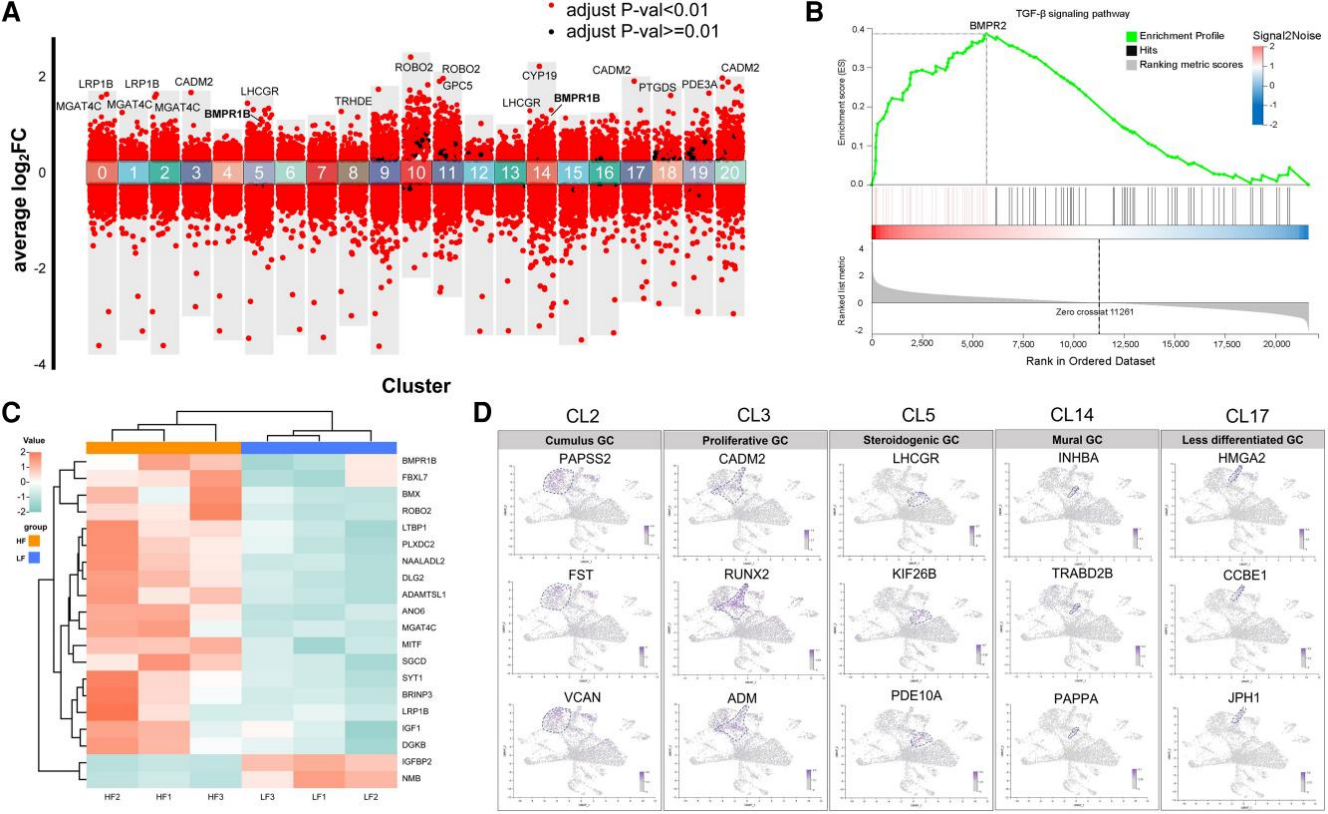
Analysis of DEGs in the ovaries of Tibetan sheep. a) Up- and downregulated genes across all clusters. The *x* axis represents CL0 to CL20, and *y* axis indicates the average log_2_FC. b) GSEA results of the KEGG pathway “TGF-β signaling pathway” is shown. c) Expression heatmap showing DEGs between HF and LF groups. d) UMAP cluster map showing expression of genes characteristic of the major ovarian GC types. Blue dashed lines indicate the boundaries of the main clusters of interest.

To clarify cell type–specific alterations in gene expression, DEGs were visualized by volcano map analysis (Fig. 4a). Previous studies indicated that *BMPR1B* was highly expressed in sheep ovaries (El-Halawany et al. 2018; Wen et al. 2021), but cell type expression levels could not be clarified. Here, we counted the number of DEGs in all clusters (supplementary fig. S9, Supplementary Material online). The *BMPR1B* gene was highly expressed in stromal cells, GCs, oocytes, and epithelial cells (supplementary fig. S10 and table S12, Supplementary Material online). The clusters 5 and 14 exhibited specifically high expression of *LHCGR* and *BMPR1B* (Fig. 4a; supplementary table S12, Supplementary Material online). Gene Ontology (GO) characteristics related to phosphorylation and steroid biosynthesis were detected across all GC clusters (CL2, 3, 5, 14, and 17), whereas the steroidogenic GC (CL5) was enriched in steroid biosynthetic and metabolic processes (supplementary fig. S11, Supplementary Material online). The *LRP1B*, *ROBO2*, and *GPC5* genes were reported to be associated with mammary and nipple development in sheep (Li et al. 2020). In the current study, the *LRP1B* (CL0 and CL2), *ROBO2* (CL10 and CL11), and *GPC5* (CL11) genes were highly expressed in HF group (Fig. 4a). The synthesis of estradiol (E_2_) by CYP19A1 in GC is related to follicle maturation and ovulation, and the inhibition of GC apoptosis (Sahmi et al. 2019). Based on GSEA, DEGs were enriched in the TGF-β signaling pathway core for *BMPR2* (Fig. 4b). *BMPR1B* genes belong to the TGF-β superfamily, which have been shown to affect ovulation rate and litter size (Mulsant et al. 2001).

To further examine DEGs in the clusters, the expression levels of DEGs per sample were demonstrated in a heatmap (Fig. 4c; supplementary table S13, Supplementary Material online). The expression of *BMPR1B* was higher in the HF group than in the LF group. Interestingly, insulin-like growth factor 1 gene (*IGF1*), a key factor related to GC steroidogenesis (Devoto et al. 1999), was also upregulated in the HF group (Fig. 4c). Notably, insulin-like growth factor binding protein 2 (*IGFBP2*) gene expression was significantly downregulated in the HF group (Fig. 4c). Dominant follicles have an enhanced ability to produce E_2_ and maintain low levels of *IGFBP2* (Austin et al. 2001). Based on the DEGs, GO enrichment analysis was performed to facilitate cluster identification (supplementary table S14, Supplementary Material online). Five cell type subpopulations were identified within GC clusters: cumulus GC (CL2), proliferative GC (CL3), steroidogenic GC (CL5), mural GC (CL14), and less differentiated GC (CL17; Fan et al. 2019; Fig. 4d; supplementary table S10, Supplementary Material online). CL2 was recognized as cumulus GC through high expression of Follistatin (*FST*). *FST* is involved in GC development and affects follicular growth (Kimura et al. 2011). Steroidogenic GC was identified based on the expression levels of cell marker *LHCGR* (Fig. 4d). CL3 was proliferative GC characterized by the high expression levels of the marker gene *RUNX2*. RUNX family transcription factor 2 (*RUNX2*) is involved in the steroidogenesis and progesterone (P_4_) synthesis in GC, which has been shown to play an important role in cell differentiation (Park et al. 2010; Fig. 4d). Additionally, LH-induced expression of *RUNX2* is important for the upregulation of specific luteal gene *PTGDS* (Park et al. 2010; Fig. 4a). Importantly, the specific expression of *PAPPA* was found in mural GC, and the latter was characterized by expression of the known marker *INHBA* (Fig. 4d). Thus, our results suggested that the *PAPPA* may be associated with the development of mural GC in the ovarian follicles.

**Fig. 5.**
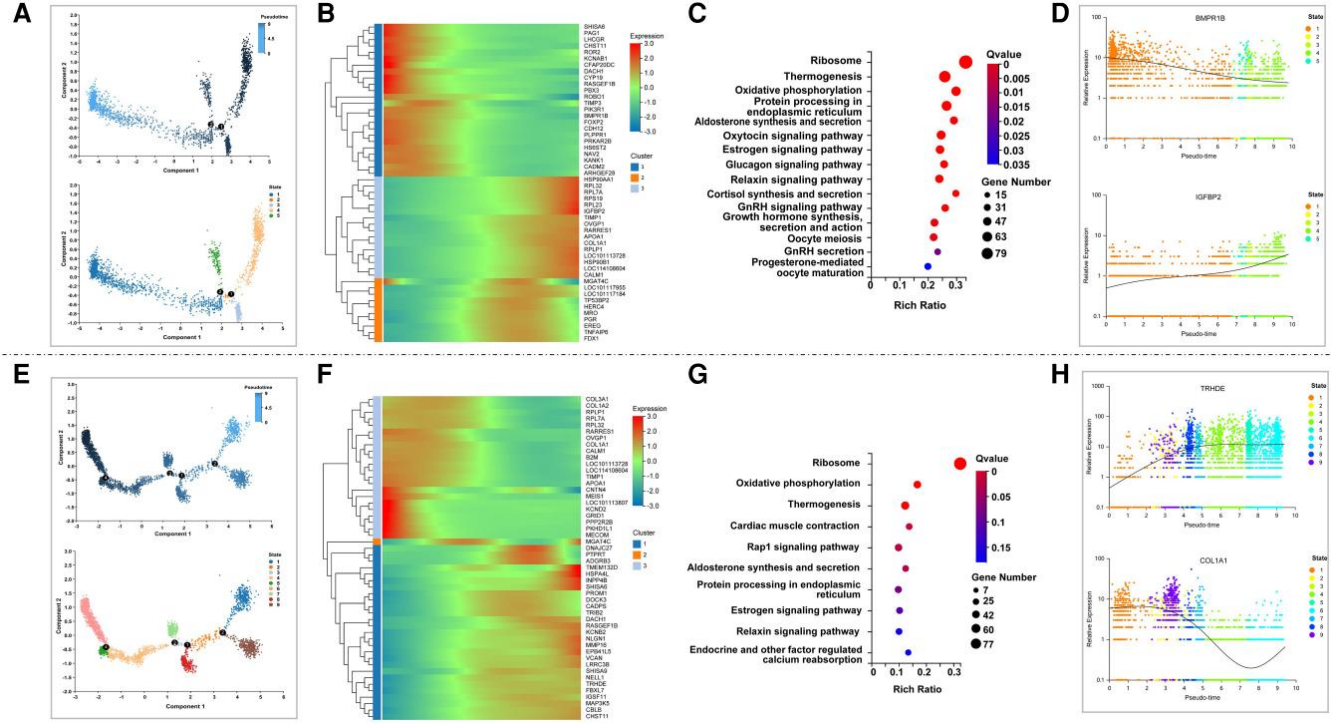
Pseudotime and clustering analyses of mural GCs and oocytes. a) Pseudotime trajectory analysis of mural GC (CL14). b) Heatmap pseudotime-dependent marker genes for mural GC subtypes. The expression level of each gene is indicated by a color gradient from low to high . c) KEGG enrichment of DEGs in ovarian mural GC subtypes of sheep with different lambing numbers. d) Expression trends of signature genes in mural GC subtypes arranged along pseudotime. e) Analysis of cell trajectories of oocytes (CL8) by Monocle. f) Pseudotime-ordered heatmap demonstrating the pseudotime order of selected marker genes for oocyte subtypes. The expression level of each gene is indicated from low to high by a color gradient from blue to red, respectively. g) KEGG enrichment of DEGs in ovarian oocyte subtypes of sheep with different lambing numbers. h) Expression plots demonstrating expression trends of the signature genes in oocyte subtypes arranged along the pseudotime.

To study the potential differentiation trajectory of the mural GC and oocytes, their developmental trajectories were constructed (Fig. 5a and e). A heatmap of potential marker genes showed their dynamic expression along the pseudotime, indicating the temporal and progressive dynamics of specific genes (Fig. 5b and f). For example, the expression level of *BMPR1B* was upregulated at the early stage and downregulated afterward (Fig. 5b). However, the expression of *IGFBP2* was maintained at medium levels at the early and middle stages and then significantly increased in the late period (Fig. 5b). Similar trends in the expression patterns of representative genes were shown along the pseudotime axis (Fig. 5d). Based on KEGG enrichment analysis, we identified that these genes were overrepresented in pathways related to steroidogenesis, such as “Aldosterone synthesis and secretion,” “Cortisol synthesis and secretion,” and “Estrogen signaling pathway” (Fig. 5c). The GCs are an integral part of the follicle and interact directly with the oocytes (Wang et al. 2020; Hu et al. 2022). Furthermore, we examine the developmental trajectory of oocytes. As shown in the oocyte pseudotime-ordered heatmap, the expression level of *TRHDE* gene was gradually upregulated in the early stage and then kept at a high level (Fig. 5f). Our analysis of single-cell data from Tibetan sheep ovaries showed that the *TRHDE* gene was specifically highly expressed in CL8 (Fig. 4a). The *TRHDE* gene was reported to be strongly associated with body temperature regulation and high-altitude adaptation (Edea et al. 2019). Meanwhile, we found that KEGG pathways of “oxidative phosphorylation,” “Thermogenesis,” and “Rap1 signaling” pathways were enriched in CL8 (Fig. 5g). The *COL1A1* gene plays an important role in oocyte maturation and embryo development (Liu et al. 2021). The expression level of *COL1A1* gene was upregulated at the early stage and downregulated afterward (Fig. 5f). A similar trend for the *COL1A1* gene could be found in gene expression patterns along the pseudotime axis (Fig. 5h). This finding indicated temporal and progressive dynamic changes in genes associated with ovarian reproduction.

### Proteomic Analysis of Tibetan Sheep Ovaries

**Fig. 6.**
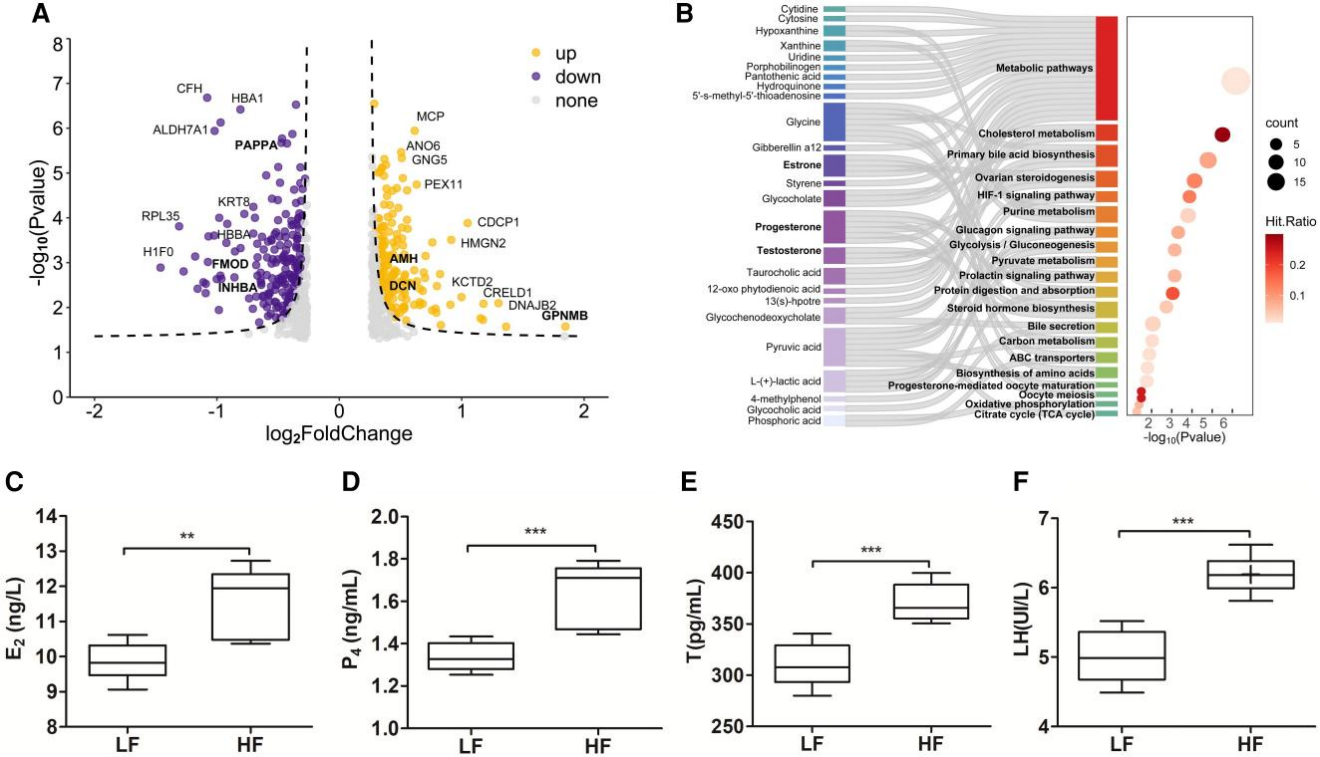
Proteomic and metabolomic analyses of ovaries and follicular fluid. a) A volcano plot shows the results of a Student's *t* test for DEPs (*P* < 0.05, fold change > 1) between the LF and HF groups. b) Sankey plot showing DMs were involved in each of the enriched pathways obtained via KEGG. Dot plot shows the ratio between DMs and the total number of metabolites in each enriched pathway (false detection rate–adjusted *P* ≤ 0.05). c to f) Determination of serum hormone levels in both groups by ELISA. E_2_, estradiol; P_4_, progesterone; T, testosterone; LH, luteinizing hormone. The bars display mean ± SD. A two-tailed *t* test was used (**P < 0.01; ***P < 0.001).

Proteome sequencing of ovarian samples from the HF and LF groups was performed to expand the findings from transcriptome analyses. This analysis identified 612 differentially expressed proteins (DEPs) in the HF and LF groups (Fig. 6a). Notably, ovarian PAPPA expression was significantly higher in LF than in HF ewes. PAPPA is a secreted metalloproteinase that cleaves IGFBP2, resulting in the release of bound IGF1 (Gerard et al. 2004). The expression level of glycoprotein nonmetastatic melanoma protein B (GPNMB) was upregulated in HF, which was consistent with the expression level of the transcript (Fig. 6a; supplementary fig. S4, Supplementary Material online). The varied expressions were observed in DEPs related to the ovarian steroidogenesis pathways (supplementary table S15, Supplementary Material online). Among all the DEPs, six (INHBA, AMH, AMHR2, FMOD, NBL1, and DCN) DEPs were associated with the TGF-β signaling pathway (supplementary fig. S12, Supplementary Material online) and were known to be functionally involved in various reproductive processes, including the regulation of fertilization, ovarian follicle development, oocyte maturation, and fertilization (Yang, Li, et al. 2019). Our results revealed that *PAPPA* plays an essential role in ovarian follicular growth, development, and steroidogenesis.

### Metabolomics of Follicular Fluid and Serum Hormone Analysis

To determine the effect of *PAPPA* on the size of dominant follicles in the ovary, liquid chromatography mass spectrometry (LC-MS) analyses were applied and identified 237 differential metabolites (DMs) in the fluid of dominant follicles. The metabolites were related to ovarian steroidogenesis, including estrone (E), P_4_, testosterone (T), and oocyte meiosis (Fig. 6b). To understand the possible regulatory signals for reproductive hormones, serum hormone levels were examined in the LF and HF groups. Serum levels of P_4_, T, luteinizing hormone (LH), and E_2_ were all higher in the HF group than in LF group (Fig. 6c to f; supplementary table S16, Supplementary Material online). The LH surge promotes the terminal differentiation of follicular cells into luteal cells (Park et al. 2010). Natural estrogens include E, E_2_, and estriol, among which E_2_ is the major circulating hormone, promoting the development of dominant follicles and protecting GC populations from apoptosis (Gong et al. 2017). Prolific ewes tend to have a shorter luteal phase than monotocous sheep of LF breeds, and there is abundant evidence that P_4_, in addition to regulating the release of LH, may also control the secretion of FSH (Bartlewski et al. 2017; supplementary fig. S13, Supplementary Material online). Several studies have shown higher T levels in sheep with larger litter sizes (Alon et al. 2021). The differential analysis confirmed that serum hormone contents were consistent with the steroid production. *STAR* mediated the rate-limiting step in ovarian steroidogenesis and P_4_ synthesis (Fang et al. 2019). This result was in an agreement with the expression trends of *STAR* transcripts. Additionally, P_4_ and E_2_ are synthesized in the GC by *CYP11A1* and *CYP19A1*, respectively (Pan et al. 2012). Our findings suggested that high levels of steroid hormones were the driving regulatory force behind ovulation rates in prolific ewes.

## Discussion

Although the Tibetan sheep have a wide distribution across the QTP, and their outstanding roles in altitude adaptation, grassland ecosystem balance, and livestock animals have been well recognized (Huang and Li 2018; Li et al. 2021; Xin et al. 2022), it remains rarely known about their phenotypic variation and the genes associated with reproductive traits. Reproductive traits, especially the litter size of livestock animals, are extremely intricate and influenced by multiple factors originating from heredity, domestication, and artificial selection (Ran et al. 2021). The role of *BMPR1B* in reproductive traits has been explored in many sheep breeds (Wen et al. 2021), and most studies simply analyzed the associations between litter size and genetic mutations. This produced challenging genetic breeding for many species, including sheep in the QTP. In this study, we investigated the reproductive traits of Tibetan sheep on the QTP by means of multiomics analysis and performed scRNA-seq for ovaries of Tibetan sheep to construct a map of the molecular signature. Transcriptomic, proteomic, and metabolomic data suggest that follicular development and ovulation are correlated well with cholesterol metabolism, ovarian steroidogenesis, and steroid hormone biosynthesis. The current work demonstrated that litter size was associated with the genetic mutation (c.746A > G, p.Q249R) in *BMPR1B*, which influences sheep reproduction through orchestrating multilevel regulatory mechanisms of AS, cell type–based hormone synthesis, and development of dominant follicles.

Previous studies on the genetics of prolificacy in sheep have emphasized the importance of *BMPR1B* (Mulsant et al. 2001; Polley et al. 2010; Wen et al. 2021). The present study found no significant difference in the expression level of *BMPR1B* in the ovaries. Based on the discovery of missense mutation (p.Q249R) in exon 8 of *BMPR1B*, we suggested that the prolificacy may be related to the mutation-induced AS of the *BMPR1B* transcript. AS is a main strategy for increasing the variety of transcripts expressed in eukaryotic cells (Singh et al. 2023). Previous studies indicated that the changes in gene expression between populations with high or low litter size were moderated in many ways, and the splicing variants were highly controlled (Ran et al. 2021). AS may play a fundamental role in reproduction. Genes essential for fertility are highly conserved in many mammals. DNA sequence variation among species, leading to amino acid substitutions and posttranscriptional modifications, including AS, is a result of natural and artificial selection (Gallego-Paez et al. 2017). For example, the mammalian follicle-stimulating hormone receptor (*FSHR*) gene encodes different splice variants, which result from exon skipping events and relate to the ovarian response to exogenous FSH stimulation (Karakaya et al. 2014). In addition, the current study used scRNA-seq to build a comprehensive cell atlas of the Tibetan sheep ovary and demonstrated that *BMPR1B* and *LHCGR* in steroidogenic GC and mural GC were differentially expressed between the HF and LF groups, and the expression level of *BMPR1B* was higher in the whole ovary. The high level of *LHCGR* in GC is necessary for preovulatory follicles to respond to the LH surge required for oocyte maturation, promotion of ovulation, and corpus luteum formation (Han et al. 2021). Previous researchers have shown that the *BMPR1B* mutation site encodes a region located near the binding site of *FKBP1A* and *BMPR1B* (Huse et al. 1999). The spatial structure of the BMPR1B protein is altered when the mutation occurs and likely affects the degree of binding between the two proteins. 3D protein structure prediction and Co-IP analysis indicated the potential functional consequence of splicing isoforms produced by the mutation in *BMPR1B* in HF ewes. These findings suggested that MXEs of *BMPR1B* caused by the mutation are the major mechanisms for highly productive ewes.

**Fig. 7.**
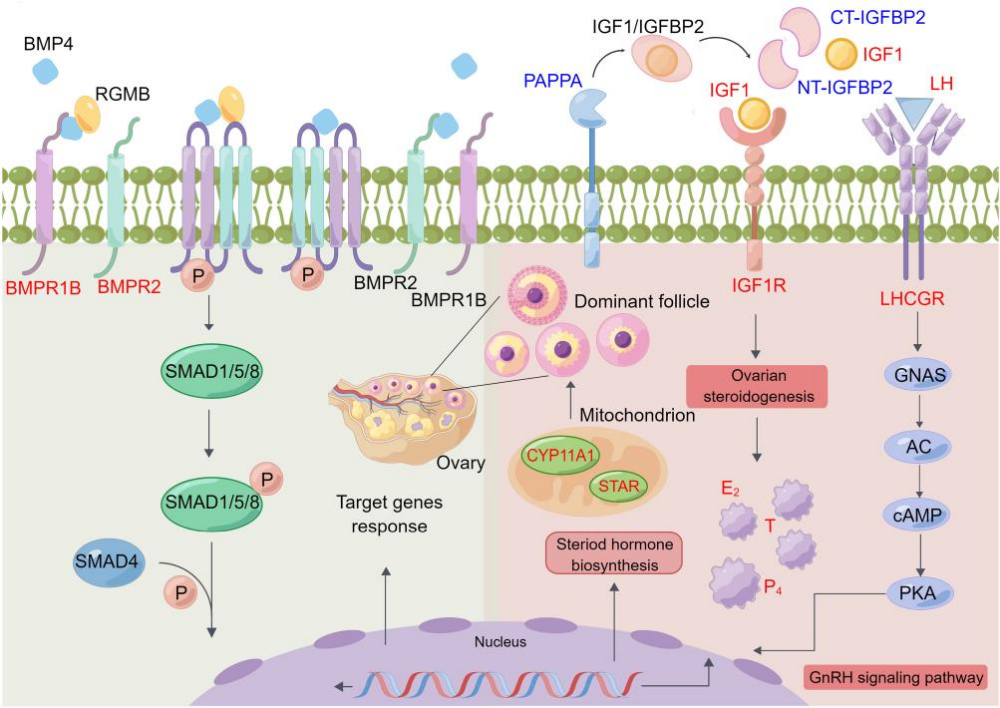
The main pathways of reproductive candidate genes for follicular growth and ovulation in Tibetan sheep. Upregulated and downregulated reproductive candidate genes during follicle growth and ovulation are highlighted in red and blue, respectively.

Lambing interval is an economically important reproductive trait directly related to reproductive efficiency and profitability in livestock farming. This study pinpointed that *PAPPA* was significantly related to lambing interval traits. We successfully mapped the first single-cell transcriptomic atlas of Tibetan sheep ovaries, providing high-quality data for revealing changes in gene expression related to ovarian GC and reproduction at the single-cell level. Using this atlas, the current study identified five subpopulations of cell types in GC clusters, including cumulus, proliferative, steroidogenic, mural, and less differentiated GC. Extensive GC proliferation and differentiation are required to support the oocyte (via cumulus GC) and allow the accumulation of follicular fluid in mural GC (Fan et al. 2019). Notably, the specific expression of *PAPPA* in mural GC is characterized by stimulating follicular growth. Together with the observation of upregulation of *IGF1* and downregulation of *IGFBP2* in the HF group, it is reasonable to assume that PAPPA regulates IGF1 through proteolysis of IGFBP-2, hereby altering IGF1 binding properties. It has been reported that IGF1 is mainly secreted from GC and plays a crucial role in the viability and growth of dominant follicles in concerted with LH, LHCGR, steroid hormone, and ovarian steroidogenesis (Han et al. 2021; Fig. 7). Similar biomarkers, such as E_2_, *LHCGR*, *CYP19A1*, and *PAPPA*, have been detected in the HF bovine with the capacity to ovulate two small follicles in the single estrous cycle (García-Guerra et al. 2018). A strong relationship between the concentrations of IGF1 and E_2_ in follicular fluid and in the formation of dominant follicles has been reported in bovine follicles (Satrapa et al. 2013). This is consistent with the metabolomic results of follicular fluid in our experiment, which was mainly expressed in the dominant follicles. In addition, previous studies found that transcription of *STAR* and *CYP11A1* were much lower in stromal cells than in luteal cells, and expression of *STAR* was higher in stromal cells than in fibroblasts (Jabara et al. 2003). Our results indicated that upregulation of *STAR* expression plays a key role in steroid hormone biosynthesis. *GPNMB* is a melanocyte-expressed gene with expression dependent on *MITF* transcription (Loftus et al. 2009), and the latter was associated with the tricarboxylic acid cycle to regulate the hypoxic response in melanoma (Louphrasitthiphol et al. 2019). Hemoglobin subunit alpha 1 (*HBA1*) is related to the pathway by which erythrocytes take up carbon dioxide and release oxygen (Storz and Bautista 2022). Aldehyde dehydrogenase 7 family member A1 (*ALDH7A1*) was reported to reduce energy consumption and promote cellular energy homeostasis during hypoxia and starvation (Yang, Hsu, et al. 2019). Other recent studies verified that the *PAPPA* mRNA in GC is not dispensable for follicular development in all species, indicating that other proteases or protease inhibitors may be involved in IGFBP degradation (Mazerbourg and Monget 2018). The IGFBP protease system consists of several enzymes that target proteolytic cleavage of IGFBPs and enable the release of IGFs, which promotes follicular growth, steroidogenesis, and oocyte maturation in most mammalian species (Silva et al. 2009). IGFBP-2 has an important role in ovarian folliculogenesis and steroidogenesis. In particular, the production of P_4_ is induced by the expression of the *STAR* gene (Balasubramanian et al. 1997). It has been reported that *STAR* plays an important role in many aspects of follicular development, including the activation of resting primordial follicles, proliferation and apoptosis of GC and membrane cells, steroid formation, gonadotropin receptor expression, oocyte maturation, ovulation, and luteinization (Elvin et al. 2000). Overall, these data indicated that the selection of dominant follicles was associated with increased proteolytic degradation of IGFBP-2 by PAPPA in the Tibetan sheep ovary, which may contribute to the bioavailability of IGF1.

Combining GWAS, transcriptomics, proteomics, and metabolomic evidence, we found that *PAPPA* may play a key role in sensitizing ovarian mural GC to *LHCGR* action during dominant follicular development, and this mutation may allow Tibetan sheep to develop smaller dominant follicles and effect the development and ovulation of dominant follicles. Previously, numerous studies identified proteolysis of IGFBP-2 as one of the primary functions of PAPPA (Monget et al. 2003; Gerard et al. 2004). The PAPPA protease-induced decrease in IGFBP-2 may cause increased levels of bioavailable IGFs that stimulate steroidogenesis and mitogenesis in the developing dominant follicles, which ultimately prepare the follicles and oocytes for successful ovulation and fertilization (Fig. 7). Whether the hormonal control of PAPPA production by the ovary differs between monotocous and polytocous animals requires further study.

## Conclusion

In summary, our work indicated that mutations and MXE events in *BMPR1B* in the Tibetan sheep population contribute to higher litter size, and *PAPPA* also plays an essential role in dominant follicle development and ovulation in Tibetan sheep ovaries (Fig. 7). Our results provide comprehensive insights into the candidate genes and adaptive potential of sheep reproduction at multiomics levels. Tibetan sheep are the only indigenous breed that can adapt to the extreme environmental conditions on the QTP. Plateau domesticated animals are subjected to strong selection pressure to improve reproductive success, which has led to the identification of animal strains with higher fecundity.

## Materials and Methods

### Experimental Animals and Grouping

All sheep used for sequencing and experiments were collected from Qinghai Province. The ewes were all approximately 5 yr old and weighed 47.7 ± 5.5 kg. The characteristics, appearance, and health status of the experimental animals were also basically the same. The experimental population was divided into two groups, where 31 HF (producing twins or triplets) and 22 LF (singleton offspring) sheep in three consecutive lambing seasons were collected and analyzed for GWAS, transcriptomic, proteomic, and metabolomic analysis to explore the genetic variation of fecundity in Tibetan sheep. Animal experiments were approved by the Animal Ethics Welfare Committee of the Northwest Institute of Plateau Biology, Chinese Academy of Sciences (Approval No. NWIPB2020302). All experiments and methods were performed according to the relevant guidelines and regulations.

### Collecting Samples and Data

Ear tip tissue was collected from ewes that had given birth to twins or triplets for more than 3 consecutive yr for whole–genome resequencing. The number of lambs and lambing interval were recorded for all test sheep. We also collected 1,130 samples to investigate genotype distribution in a large population. Experimental ewes were estrus synchronized to ensure that they were in the same state. All ewes received an intramuscular injection of 0.2 mg of cloprostenol sodium (Sansheng Biological Technology Co., Ltd, Ningbo, China) on day 0 (a random day of the estrous cycle or anovulatory period) and day 10 to effect synchronization of estrus. Subsequently, the estrus period was detected by teasing the rams, and the ewes were checked for marks three times a day to detect the onset of behavioral estrus. All ewes were placed under a uniform management system, fed corn silage, and balanced feed ratios twice daily, with unrestricted access to water and mineral salt licks. Samples were collected at the beginning of the second natural estrus period. Ewes were nonpregnant and free from any anatomical reproductive disorders. Then, blood was collected from the experimental sheep. Tibetan sheep were ethically sacrificed, ovaries were immediately removed, and follicular fluid was collected. The diameter of follicles was measured by a vernier caliper, and the estimated volume (*V*) of a dominant follicle was calculated using the following formula (Oliveira et al. 2016): *V* = 4/3 × *π* × *r*^3^. The ovarian weight/body weight ratio was also calculated (supplementary table S5, Supplementary Material online). Follicular fluid samples were aspirated from dominant follicles with a 22-gauge needle attached to a 2-mL sterile syringe (BD, Franklin Lakes, NJ, United States; Nandi et al. 2007). Six ovaries were derived from six independent sheep. All samples were washed with phosphate buffered saline twice to remove blood and then cut into small pieces, frozen instantly using liquid nitrogen, and stored at −80 °C until further analysis. Information on the animals tested for the LF and HF groups in the experiments, including their number, litter size, parity number, and lambing interval, is presented in supplementary table S1, Supplementary Material online.

### Whole–Genome Resequencing

Ear tip tissue samples were collected from LF and HF groups. High-quality DNA was extracted using a TIANGEN TIANamp Genomic DNA Kit. DNA purity and integrity were analyzed by 1% agarose gel electrophoresis. DNA purity was also checked using a NanoDrop spectrophotometer (Thermo, CA, United States). DNA concentration was measured using a Qubit DNA Assay Kit in Qubit 2.0 Fluorometer (Life Technologies, CA, United States). The prepared library was sequenced on the Illumina NovaSeq 6000 platform, and 150-bp paired-end reads were generated.

### Variant Detection

The quality of the generated raw reads was controlled using fastp (v0.21) with default parameters (Chen et al. 2018). Contaminated joints and low-quality reads with uncalled base content higher than 5% were removed using the ARS-UI_Ramb_v2.0 (GCF_016772045.1) reference genome. High-quality clean reads were mapped to the updated reference genome using BWA-MEM (v0.7.17; Li and Durbin 2009). Output alignments in bam format were marked in duplicates and sorted using SAMtools (v1.9; Li et al. 2009). Subsequently, the Genome Analysis Toolkit (v4.0.11.0; Brouard et al. 2019) was used to execute local realignment of reads to further improve the quality of alignments in regions around putative indels. The multiway pileup method implemented in BCFtools (v1.9; Danecek et al. 2021) was applied to generate a pileup file to be used for SNP identification. To obtain high-quality SNPs for analysis, the threshold of minimum mapping quality, minimum base quality, and adjusted mapping quality were set to 20, 20, and 50, respectively.

### GWAS and Population Genomic Analysis

The plink (v1.90; Chang et al. 2015) was used for GWAS analysis. The SNP sites with minimum allele frequency < 0.1, deletion rate of all individuals > 0.1, and Hardy–Weinberg *P* < 10^−5^ were filtered, with LF as the control group and HF as the experimental group. GWAS analysis was performed using Fisher's exact test with parameters “- assoc fisher”. The analysis results were visualized using the R package qqman (v0.1.4; Turner 2018). SnpEFF (v4.3t; Cingolani et al. 2012) was applied to annotate each variant using the annotation file in GTF format prepared for the ARS-UI_Ramb_v2.0 reference genome.

### KASP Variant Genotyping

To validate the GWAS signals, we performed an association analysis using a larger collection of samples (*n* = 1,130), including 159 HFs and 971 LFs that were sampled from Qinghai Province (supplementary table S4, Supplementary Material online). The KASP method was used for the validation of GWAS signals (Wuhan Tianyi Huayu Gene Technology Co., Ltd.). Based on SNP site sequence information, primers for the KASP reaction were designed using the online primer design tool SNPWay (http://www.snpway.com/). Primer information is shown in supplementary table S17, Supplementary Material online. The final reaction was done in a total volume of 5 μL, which contained 1 μL DNA template (50 ng/μL), 2.5 μL 2× KASP Master Mix (Parms, Gentides Biotech Co., Ltd.), 0.15 μL Primer Mix (10 μm each of forward primer, reverse primer, and common primer), and 1.35 μL ddH_2_O. PCR thermocycling was done as follows: initiation at 94 °C for 15 min; 10 cycles of denaturation at 94 °C for 20 s and touchdown annealing from 65 °C (−0.7 °C/cycle) for 60 s; followed by 30 cycles of denaturation at 94 °C for 20 s and annealing at 57 °C for 60 s; and finished by an extension at 30 °C for 60 s. The QuantStudio 5 Real-Time PCR System (Applied Biosystems, Thermo, CA, United States) was used for PCR amplification.

### Transcriptome Sequencing and Analysis

Total RNAs were extracted from ovarian samples collected from three LF and three HF ewes by TRIzol (Invitrogen, CA, United States). After monitoring degradation and contamination using 1% agarose gels, RNA purity was checked using a NanoDrop spectrophotometer (Thermo, CA, United States). RNA concentration was measured using a Qubit RNA Assay Kit and Qubit 2.0 Fluorometer (Life Technologies, CA, United States). RNA integrity was assessed using the RNA Nano 6000 Assay Kit with the Agilent Bioanalyzer 2100 system (Agilent Technologies, CA, United States). In total, 1.5 μg of RNA per sample was used as input material for RNA sample preparations. Sequencing libraries were generated using a MGIEasy RNA Library Prep Kit (BGI Genomics Co., Ltd., Shenzhen, China) following the manufacturer's recommendations. The library was sequenced on a MGISEQ-2000 platform, and 100-bp paired-end reads were generated (BGI Genomics Co., Ltd., Shenzhen, China). SOAPnuke (Cock et al. 2010) was used for filtering with parameters “-l 15 -q 0.2 -n 0.05”. After quality control of reads’ data, a reference genome index was built and high-quality RNA-seq reads were aligned to the reference genome using HISAT2 (v2.0.4; Kim et al. 2015) with parameters “--sensitive --no-discordant --no-mixed -I 1 -X 1000 -p 8 --rna-strandness RF”. Differential expression analysis was conducted using the R package DESeq2 (v1.38.3; Love et al. 2014) based on read count numbers. The significance of gene expression differences was determined using the Wald test, with |log_2_fold-change| ≥ 1 and “*Q* value” (adjusted *P*-value) < 0.05 considered noteworthy. GO and KEGG pathway enrichment analyses were then performed, and coding genes associated with ovarian reproduction and their biological functions were identified using the R package cluster-Profiler (v3.10.1; Yu et al. 2012).

### AS Events and Structural Prediction Analysis

The reads were compared to the reference genome (GCF_016772045.1), and then rMATS (v3.2.5; Shen et al. 2014) was used to detect differential AS events with a false discovery rate (FDR) ≤ 0.05 and |IncLevelDifference| ≥ 0.1. We detected five of these variable splicing events, including SE, A5SS, A3SS, MXE, and RI. The protein 3D structures of wild-type and mutant BMPR1B were predicted based on results of AS event analysis, using AlphaFold2 (v2.0.0) in casp14 mode (Jumper et al. 2021) and then visualized using Pymol (v2.5.5; Mooers 2020).

### Co-IP and IB

Genes, including those with variable splicing encoding B1, B2, and B3 of *BMPR1B*, as well as *FKBP1A* and *BMP4*, were synthesized by Sangon Biotech Co., Ltd. (Shanghai, China). The five genes were cloned into the *Eco*RI–*Xho*I and *Not*I–*Eco*RI sites of pCMV-HA or p3xFLAG-CMV-10 vectors, respectively, to express proteins with tags. The Co-IP and IB were performed as described previously (Zhong et al. 2018). 293T cells were purchased from the American Type Culture Collection (Manassas, VA, United States) and cultured in 100 mm Petri dishes until reaching 80% to 90% confluency. Cells were transfected with 5 μg of BMPR1B (B1/B2/B3)-Flag with or without 5 μg of FKBP1A-HA or BMP4-HA. Two days after transfection, cells were lysed in lysis buffer (Solarbio) and subjected to Co-IP according to the manufacturer's instructions (Solarbio). Cell lysates were incubated with protein-A-Sepharose beads conjugated with anti-HA antibodies at 4 °C overnight. After washing five times, the precipitates were resuspended in sodium dodecyl sulfate – polyacrylamide gel electrophoresis (SDS-PAGE) sample buffer, boiled for 4 min, and run on a 10% SDS-PAGE gel. IB was conducted with mouse monoclonal anti-Flag or anti-HA (Abcam) antibodies.

### Preparation of Ovarian Tissue for Single-Cell RNA-seq

A typical estrus stage ovary containing a dominant follicle with a diameter of 5 to 9 mm and several antral follicles with diameters under 5 mm was selected for the subsequent scRNA-seq experiment. A fresh ovary was flushed with normal saline to remove blood and then minced into approximately 3 mm cubed pieces with a sterile scalpel blade. In total, six ovarian pieces were obtained and then cut into small pieces, instantly frozen using liquid nitrogen, and stored at −80 °C until further analysis.

### Single-Cell Dissociation of Ovarian Tissue

Ovarian samples were dissociated for single-cell transcriptomics as previously described (Chen, Xu, et al. 2021). Around 100 mg flash-frozen minced tissue in liquid nitrogen was transferred to a tissue homogenizer (on ice) containing 2 mL prechilled homogenization buffer and incubated for 3 to 5 min until the tissue was fully infiltrated. The tissue was homogenized around 10 to 15 times. Sample suspensions were assessed by microscopy to determine if nuclei were completely released into suspension. Forty-micrometer cell strainers were used to filter the tissue homogenate, followed by collection into a 15-mL centrifuge tube. Samples were centrifuged at 500 × *g* and 4 °C for 5 min, then supernatants were discarded and cell pellets were washed twice with wash buffer. The precipitate was resuspended in the desired volume of blocking buffer and mixed thoroughly. The nuclei suspension was counted and stored for subsequent experiments.

### Single-Cell RNA-seq

Chromium Next GEM Single Cell 3′ Reagent Kit v3.1 (PN-1000121, 10x Genomics, United States) was used for Gel Beads-in-emulsion (GEM) formation. The cell suspensions, gel beads, and partitioning oil were loaded into a Chromium Single Cell Controller to produce single-cell GEMs. Then, a primer containing read 1 sequencing primer, 16 nucleotide 10x Barcode, and 12 nucleotide unique molecular identifier were applied to produce barcoded full-length cDNA by reverse transcriptase reaction. After incubation, GEMs were broken and pooled fractions were recovered. First-strand cDNAs were purified with silane magnetic beads and amplified to generate sufficient mass for library construction. Enzymatic fragmentation and size selection were used to optimize cDNA amplicon size. Read 1 primer sequence was added to the molecules during GEM incubation. P5, P7, a sample index, and read 2 primer sequence were added via end repair, A-tailing, adaptor ligation, and PCR. Subsequently, the library was sequenced on a DNBSEQ sequencing platform (MGISEQ-2000, BGI Genomics Co., Ltd., Shenzhen, China), and paired-end readings of 100 bp were generated.

### Primary Sequencing Analysis and Original Data Generation

Raw scRNA-seq data were converted using the Cell Ranger (v 5.0.1; Zheng et al. 2016) analysis pipeline provided by 10x Genomics, and reads were aligned to the sheep genome version ARS-UI_Ramb_v2.0 using the STAR aligner (Dobin et al. 2013). Cell Ranger output in the form of a “filtered gene-barcode” count matrix, containing the expression profiles of cells with correctly detected cellular barcodes, was used for downstream analyses.

### Quality Control of Single-Cell Data and Determination of Major Cell Types

The raw gene expression matrices generated from each sample were aggregated using Cell Ranger (v5.0.1) provided on the 10x Genomics website. Downstream analysis was done using the R package Seurat (v 3.2.0). Quality control was applied to cells based on number of detected genes and proportion of mitochondrial reads per cell. Specifically, cells with less than 200 detected genes or cells with > 90% of the proportion of maximum genes were filtered out. For the mitochondrial metric, cells were sorted in descending order of mitochondrial read ratio, and the top 15% of cells were filtered out. Potential doublets were identified and removed by DoubletDetection (https://rdrr.io/github/scfurl/m3addon/man/doubletdetection.html). Cell cycle analysis was performed by using the CellCycleScoring function in Seurat program. The gene expression data set was normalized, and subsequent principal component (*n* = 15) analysis was conducted using only the 2,000 most highly variable genes in the data set.

U-MAP was then used for 2D visualization of the resulting clusters. For each cluster, the marker genes were identified using the “FindAllMarkers” function as implemented in the Seurat package (logfc.threshold > 0.25, minPct > 0.1, and Padj ≤ 0.05). Then, cell clusters were automatically annotated using the SCSA (https://github.com/bioinfo-ibms-pumc/SCSA) method based on marker genes. The software SCSA integrates the information from Cellarer, CancerSEA, GO and many other databases. Subsequently, clusters were manually annotated as distinct cell types considering the SCSA annotation results, CellMarker data set, previous relevant references, and biological functions of the characteristic genes. The distinct annotated cell types were demonstrated in a UMAP plot by the “DimPlot” function. A showcase of diagrams, including dot plot, feature plots, violin plots, and heatmap, was produced by the “DotPlot”, “FeaturePlot”, “VlnPlot”, and “DoHeatmap” functions, respectively, to display expression specificity of the marker genes in each cell type. DEGs across different samples were identified using the “FindMarkers” function in Seurat with parameters “logfc.threshold > 0.25, minPct > 0.1 and Padj ≤ 0.05”.

### Marker Gene or DEG Function Analysis

GO and KEGG pathway analyses were performed using phyper (https://stat.ethz.ch/R-manual/R-devel/library/stats/html/Hypergeometric.html), a function of R. Then the “pvalue” was corrected by multiple testing, and the corrected package was “qvalue” (https://bioconductor.org/packages/release/bioc/html/qvalue.html). Finally, “qvalue” (corrected “pvalue”) ≤ 0.05 was used as the threshold, and the GO term that satisfied this condition was defined as the GO term or KEGG pathway that was significantly enriched for candidate genes. GSEA v 4.1.0 was used to perform KEGG enrichment analysis with the Molecular Signatures Database (MSigDB, v7.5.1, http://software.broadinstitute.org/gsea/msigdb).

### Developmental Trajectory Inference

To understand the transcriptional dynamics that occurred in GC and oocytes, pseudotime trajectory analysis was performed to predict continuous cell states in the sheep ovary. The Monocle2 (http://cole-trapnell-lab.github.io/monocle-release/docs/) uses reversed graph embedding to describe multiple fate decisions in a fully unsupervised manner. We used Monocle2 (Trapnell et al. 2014) to do pseudotime analysis.

### Proteome Analyses

Protein extraction from the tissues was carried out using the cold acetone method as described previously (Wu et al. 2014). Extracted proteins were subjected to trypsin digestion, followed by tandem mass tag labeling. A Shimadzu LC-20AD liquid phase system with a Gemini C18 column (5 μm, 20 cm, and 180 μm) was used for liquid phase separation of the sample. Dried peptide samples were reconstituted with mobile phase A (5% acetonitrile [ACN], pH 9.8) and injected, then eluted at a flow rate of 1 mL/min by the following gradients: 5% mobile phase B (95% ACN, pH 9.8) for 10 min; 5% to 35% mobile phase B for 40 min; 35% to 95% mobile phase B for 1 min; mobile phase B for 3 min; and 5% mobile phase B for 10 min. The elution peak was monitored at a wavelength of 214 nm, and one component was collected per minute, and the samples were combined according to the chromatographic elution peak map to obtain 20 fractions, which were then freeze dried. The dried peptide samples were reconstituted with another mobile phase A (2% ACN, 0.1% formic acid [FA]), centrifuged at 20,000 × *g* for 10 min, and the supernatant was used for injection. Separation was performed by Thermo UltiMate 3000 UHPLC. The sample was first enriched in a trap column and desalted, and then entered a C18 column (75 μm internal diameter, 3 μm column size, and 25 cm column length) and eluted at a flow rate of 300 nL/min by the following effective gradient: 0 to 5 min, 5% mobile phase B (98% ACN, 0.1% FA); 5 to 45 min, mobile phase B linearly increased from 5% to 25%; 45 to 50 min, mobile phase B increased from 25% to 35%; 50 to 52 min, mobile phase B from 35% to 80%; 52 to 54 min, 80% mobile phase B; and 54 to 60 min, 5% mobile phase B. The nanoliter liquid phase separation system was directly connected to the mass spectrometer.

Peptides separated by liquid phase chromatography were ionized by a nanoESI source and then passed to a tandem mass spectrometer Orbitrap Fusion Lumos (Thermo Fisher Scientific, San Jose, CA, United States) for data-dependent acquisition mode detection. The main parameters were set as follows: ion source voltage at 2 kV, MS1 mass spectrometer scanning range at 350 to 1,500 m/z; resolution at 60,000; MS2 starting m/z fixed at 100; and resolution at 15,000. The ion screening conditions for MS2 fragmentation were charge 2+ to 6+ and the top 30 parent ions with peak intensity exceeding 20,000. The ion fragmentation mode was high-energy C-trap dissociation, and the fragment ions were detected in Orbitrap. Dynamic exclusion time was set to 30 s. The automatic gain control was set to MS1 1E5, MS2 2E4.

Automated software IQuant (Wen et al. 2014) was used for quantitatively analyzing the labeled peptides with isobaric tags. To assess the confidence of peptides, the peptide–spectrum matches (PSMs) were prefiltered at a PSM-level FDR of 1%. Then based on the parsimony principle, identified peptide sequences were assembled into a set of confident proteins. Picked protein FDR strategy of 1% was selected to control the rate of false positives at protein level (Savitski et al. 2015). Proteins were considered differentially expressed if *P* ≤ 0.05. GO and KEGG pathway enrichment analysis for identifying proteins was performed, and their biological functions were identified using the R package clusterProfiler (v3.10.1; Yu et al. 2012).

### Metabolome Analyses

LC-MS was used for untargeted metabolomic analysis to determine metabolite differences between LF and HF ewes. Fluid from dominant follicles was collected as one pool after slaughter, and six biological replicates for each group were tested. Follicular fluid samples were frozen in liquid nitrogen for 30 min, and then metabolites were extracted. Metabolite profiles were detected by high-performance liquid chromatography (Waters 2D UPLC, Waters, United States) and high-resolution mass spectrometer (Q exactive HF, Thermo Fisher Scientific, United States). Metabolite data in both positive and negative ion modes were collected to improve metabolite coverage and accuracy. LC-MS data processing was performed using The Compound Discoverer 3.0 (Thermo Fisher Scientific, United States) software, mainly combined with BGI library, mzCloud, and ChemSpider (HMDB, KEGG, and LipidMaps). Data preprocessing, statistical analysis, metabolite classification annotations, and functional annotations were performed using the metabolomic R package metaX (Wen et al. 2017) and the metabolome bioinformatic analysis pipeline. The multivariate raw data were dimensionally reduced by principal component analysis to determine the groupings, trends (intra- and intergroup similarities and differences), and outliers of observed variables in the data set (whether there is an abnormal sample). Using partial least squares discriminant analysis, the “variable importance in projection” values of the first two principal components of the model, combined with the variability analysis, the fold change, and Student's test, were used to screen for DMs. DM screening conditions were as follows: (i) partial least squares discriminant analysis model with “variable importance in projection” ≥ 1 for the first two principal components; (ii) “FoldChange” ≥ 1.2 or ≤0.83; and (iii) “pvalue” < 0.05. Pathway enrichment analysis identified significantly enriched metabolic pathways or signal transduction pathways in DMs compared with the whole background.

### Enzyme-Linked Immunosorbent Assay

Blood samples from the jugular vein of ewes were collected into vacuum tubes prior to sampling (KANG JIAN MEDICAL, Jiangsu, China). Blood was naturally clotted at room temperature for 10 to 20 min, centrifuged at 2,000 × *g* for 20 min, and serum samples carefully collected and stored at −20 °C for later hormone measurements. Progesterone, E_2_, T, follicle-stimulating hormone (FSH), and LH were detected with P_4_ (PN-BYE80231), E_2_ (PN-BYE93019), T (PN-BYE80193), FSH (PN-BYE93020), and LH (PN-BYE93021) ELISA kits for sheep according to the manufacturer’s instructions (BangYi Biotech Co., Ltd., Shanghai, China).

## Supplementary Material

msae058_Supplementary_Data

## Data Availability

All data supporting the findings in this study are available within this article and its supplementary files. All the sequencing data reported in this study are available upon request for research purpose.
