# Supplementary material for: Multiomics Analyses Provide New Insight into Genetic Variation of Reproductive Adaptability in Tibetan Sheep
Source: Mol Biol Evol. 2024 Mar 29;41(3):msae058. doi: 10.1093/molbev/msae058 (PMC10980521; doi:10.1093/molbev/msae058)
Supplement: msae058_Supplementary_Data [file msae058_supplementary_data.pdf]

Supplementary Information for
“Multi-omics analyses provide new insight into genetic variation of reproductive adaptability in Tibetan sheep”

Buying Han[1,2,3], Dehong Tian[1,3], Xue Li[1,3], Sijia Liu[1,3], Fei Tian[1,3], Dehui Liu[1,2,3], Song Wang[1,2,3], and Kai Zhao[1,3*]

[1] Key Laboratory of Adaptation and Evolution of Plateau Biota, Northwest Institute of Plateau Biology, Chinese Academy of Sciences, Xining 810001, China
[2] University of Chinese Academy of Sciences, Beijing 100049, China
[3] Qinghai Provincial Key Laboratory of Animal Ecological Genomics, Northwest Institute of Plateau Biology, Chinese Academy of Sciences, Xining 810008, China

* Correspondence: zhaokai@nwipb.cas.cn

The supplementary file includes:

Figures S1 to S13
Tables S1 to S17
SI References

Figures S1 to S13

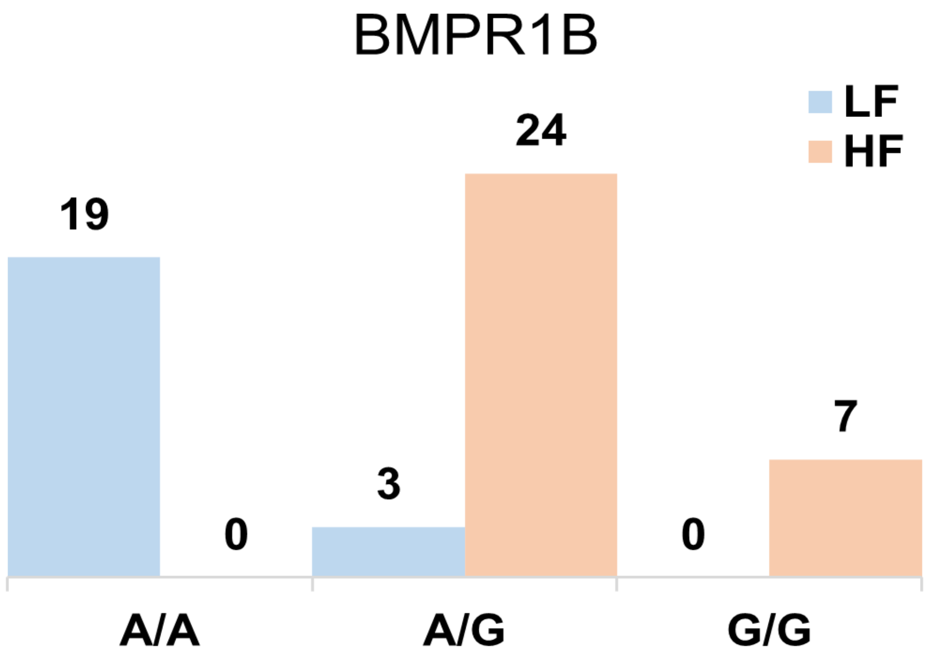

Fig. S1. Correlations between Tibetan sheep litter size phenotypes and genotypes of *BMPR1B*.

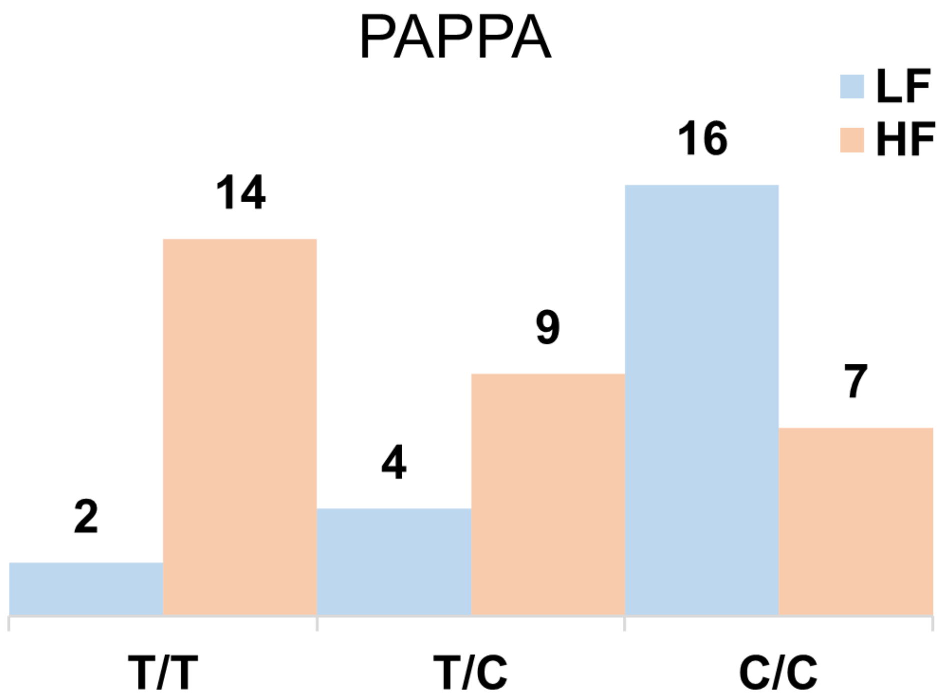

Fig. S2. Correlations between Tibetan sheep lambing interval phenotypes and genotypes of *PAPPA*.

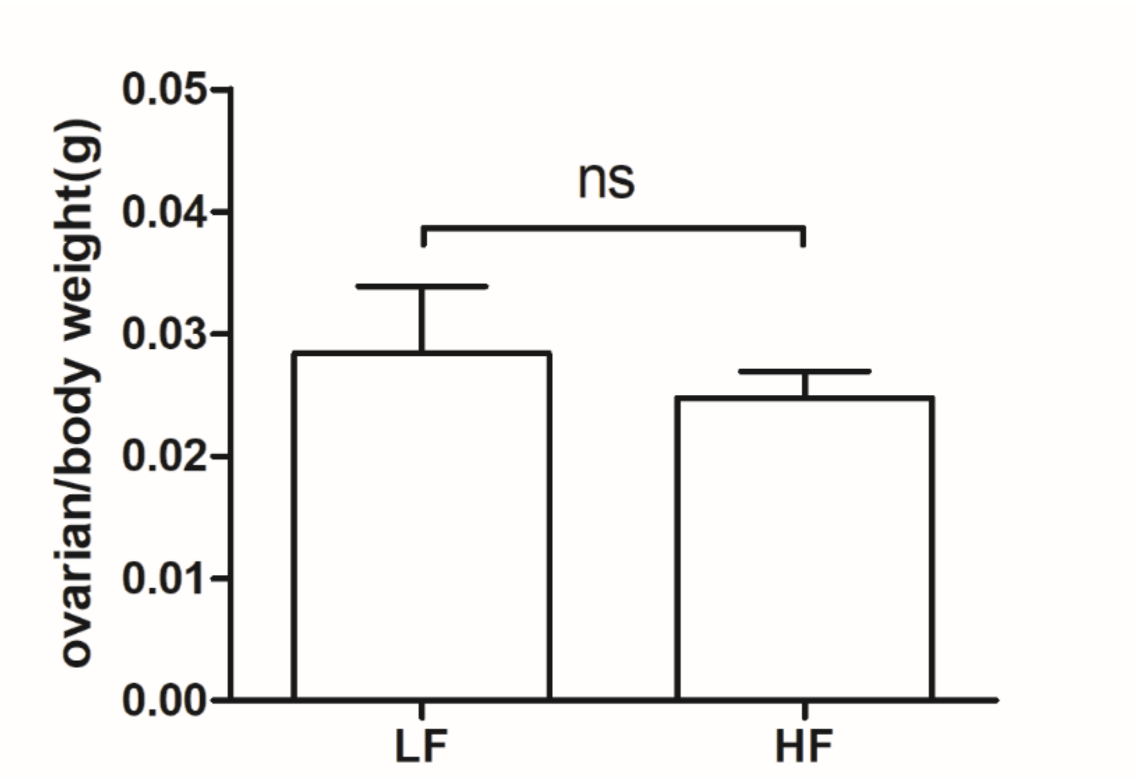

Fig. S3. Statistics of the ovarian/body weight in two groups of Tibetan sheep (*$P < 0.05$; **$P < 0.01$; ***$P < 0.001$; ns, not significant).

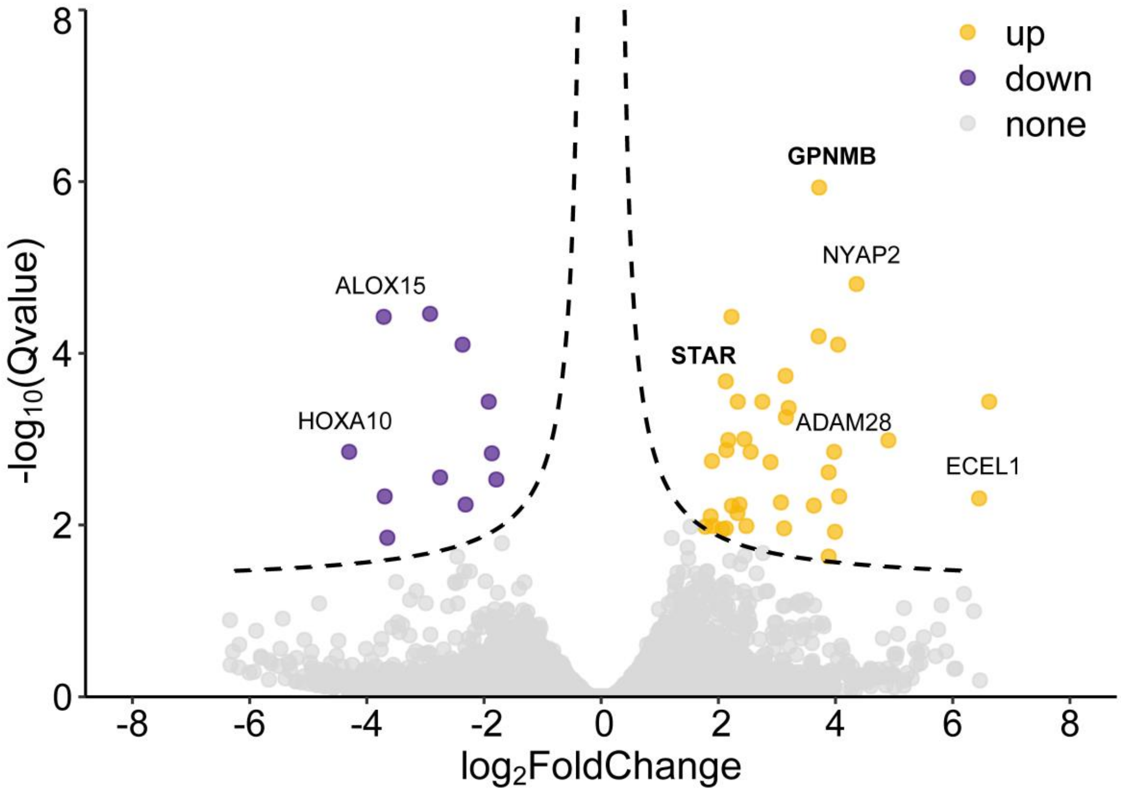

Fig. S4. A volcano plot shows the results of a Student's t test LF and HF groups of differentially expressed genes (DEGs) (Qvalue < 0.05, FoldChange >1), light yellow indicates up-regulated, light purple indicates down-regulated.

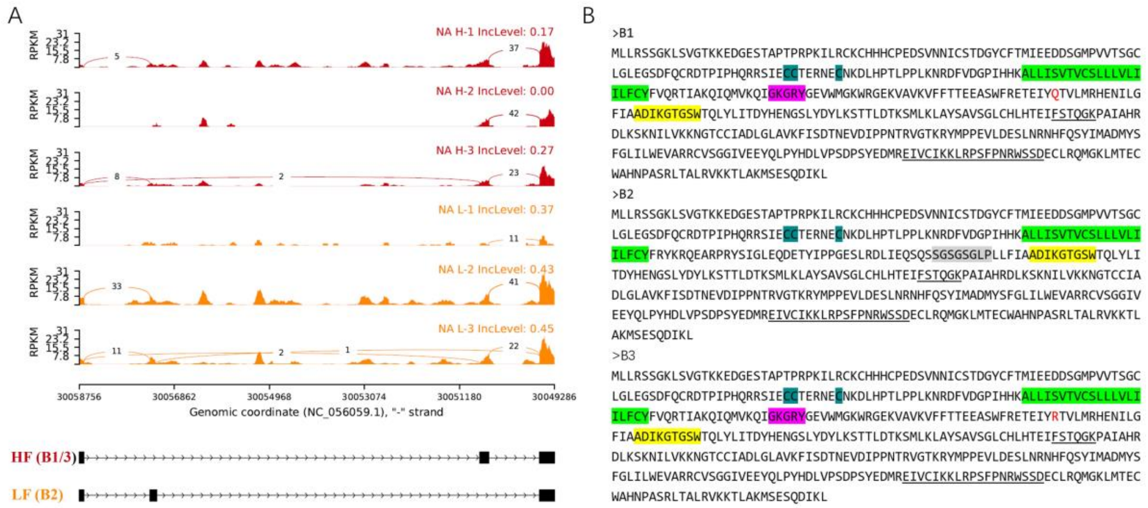

Fig. S5. Analysis of transcriptomics and alternative splicing events. (A) Use IGV software shows alternative sites for the *BMPR1B* gene as indicated from RNA-seq results. Black boxes indicate splice sites. HF group (H-1, H-2, and H-3), LF group (L-1, L-2, and L-3). (B) Amino acid sequences of three variable splicing types. Cysteine residues in the extracellular domain (cyan), transmembrane domain (green), GS motif (grey), ATP binding site (magenta), loop 45 (yellow), kinase inserts (underlined), red highlighted R represents amino acids after mutation.

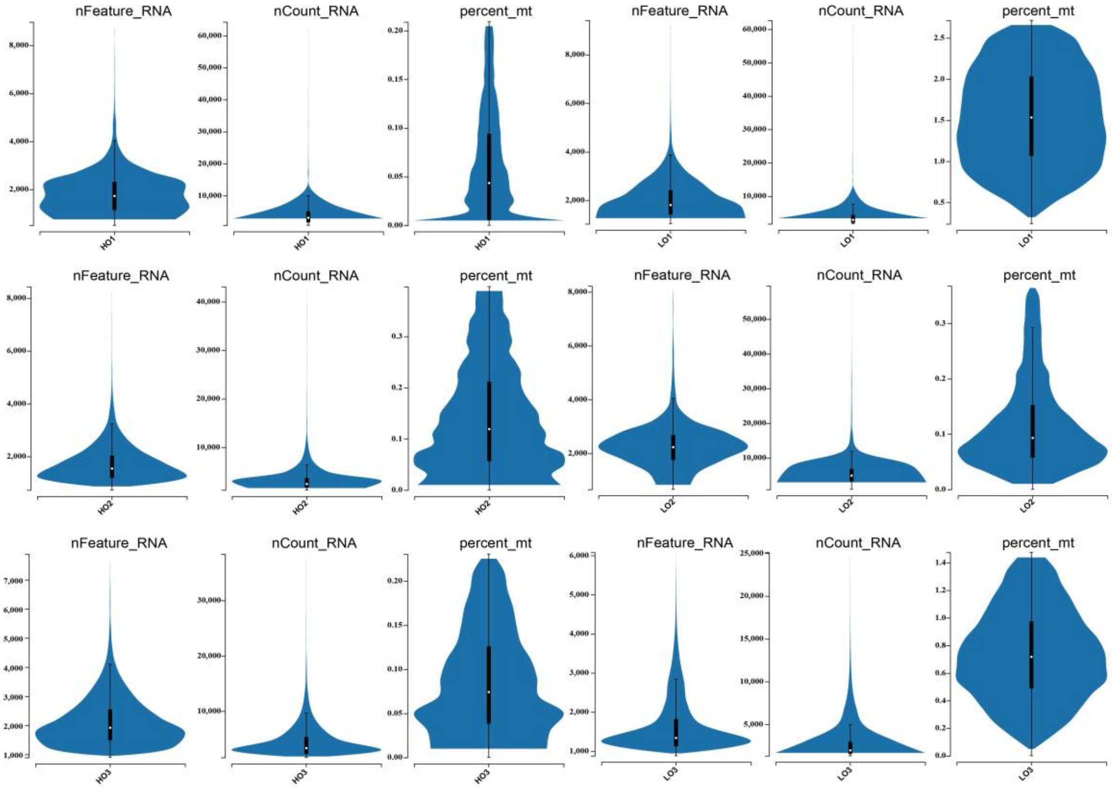

Fig. S6. QC violin plot. nFeature_RNA: the violin plot of mRNA expression; nCount_RNA：the violin plot of mRNA read counts; percent_mt: the violin plot of mitochondrial RNA ratio.

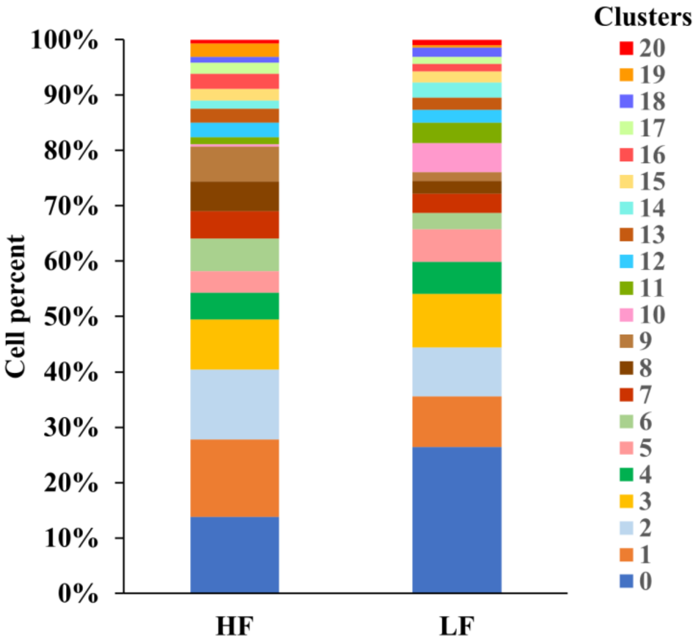

Fig. S7. All clusters were present in the HF and LF groups.

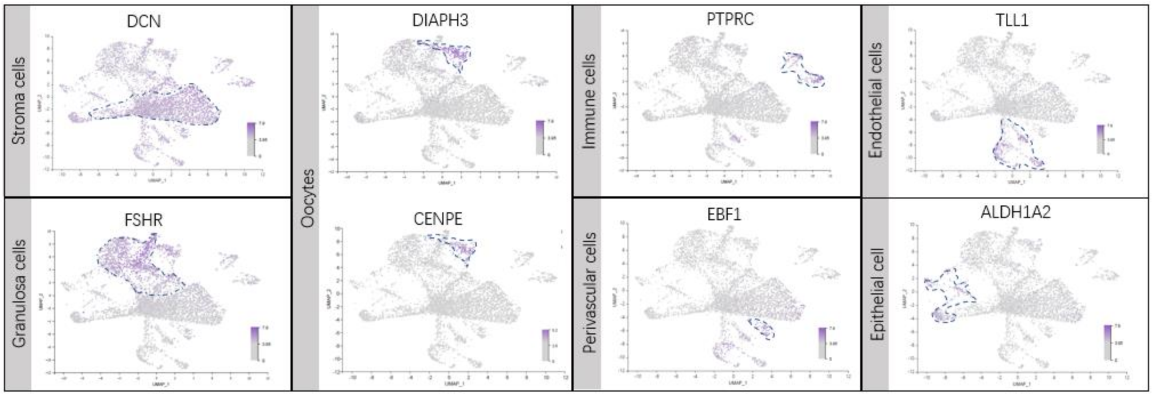

Fig. S8. UMAP cluster map showing expression of genes characteristic the major ovarian granulosa cell types. Blue dashed lines give the boundaries of the main clusters of interest.

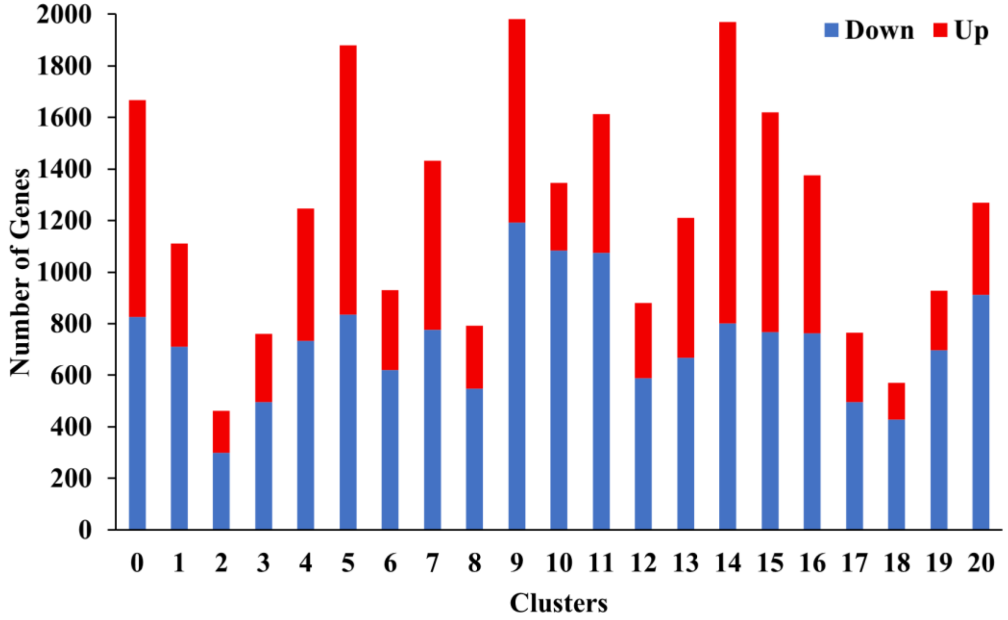

Fig. S9. Statistical plot of the number of differentially expressed genes (DEGs) in all clusters.

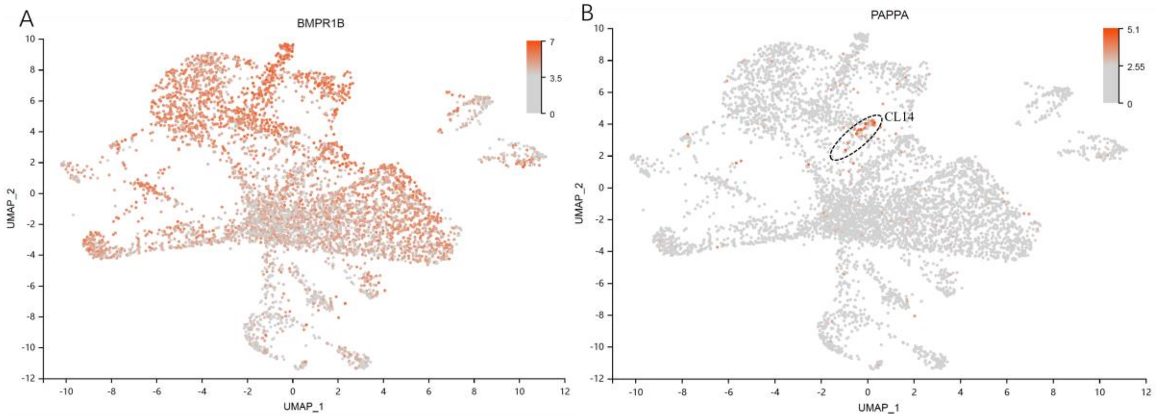

Fig. S10. UMAP cluster map showing expression of *BMPR1B* (A) and *PAPPA* (B) genes characteristic in all clusters.

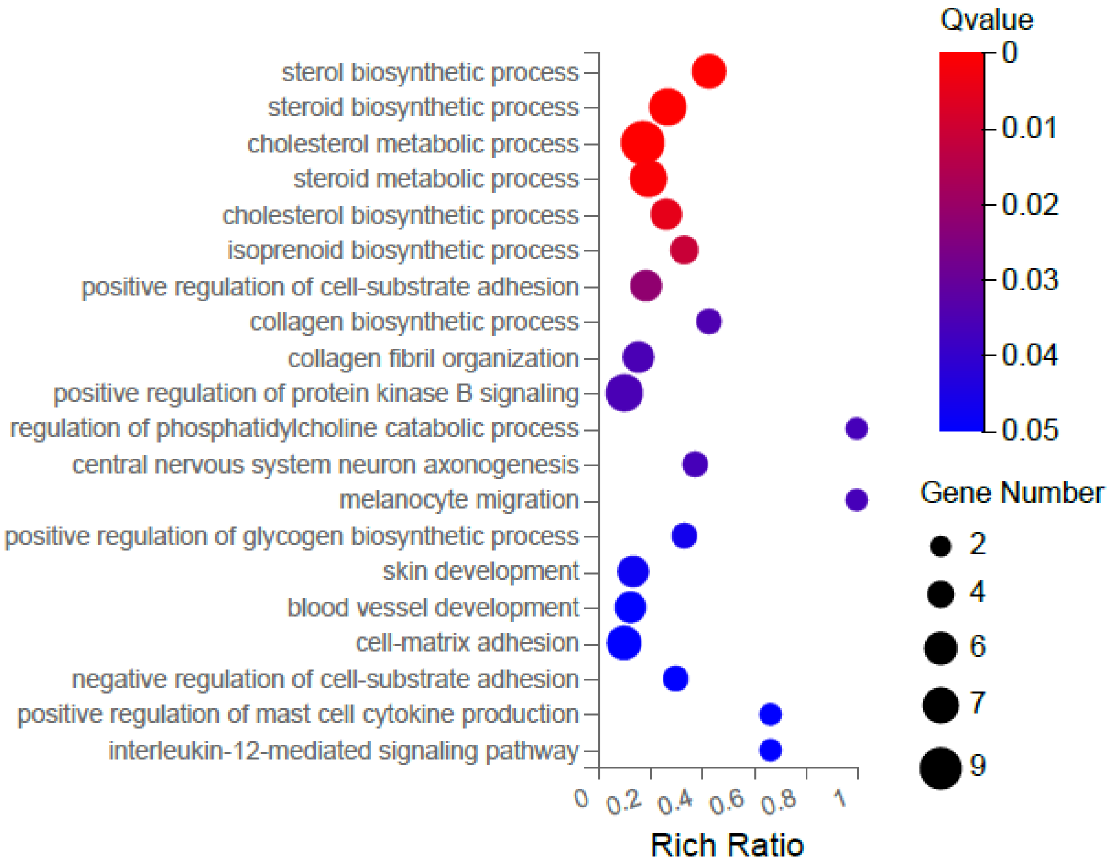

Fig. S11. GO biological process enrichment bubble chart (Cluster5). X-axis indicated enrichment ratio (enrichment Ratio=Term Candidate Gene Num / Term Gene Num), Y-axis indicated GO Term, bubble size indicated marker gene number annotated by GO term, color density indicated significance (Qvalue).

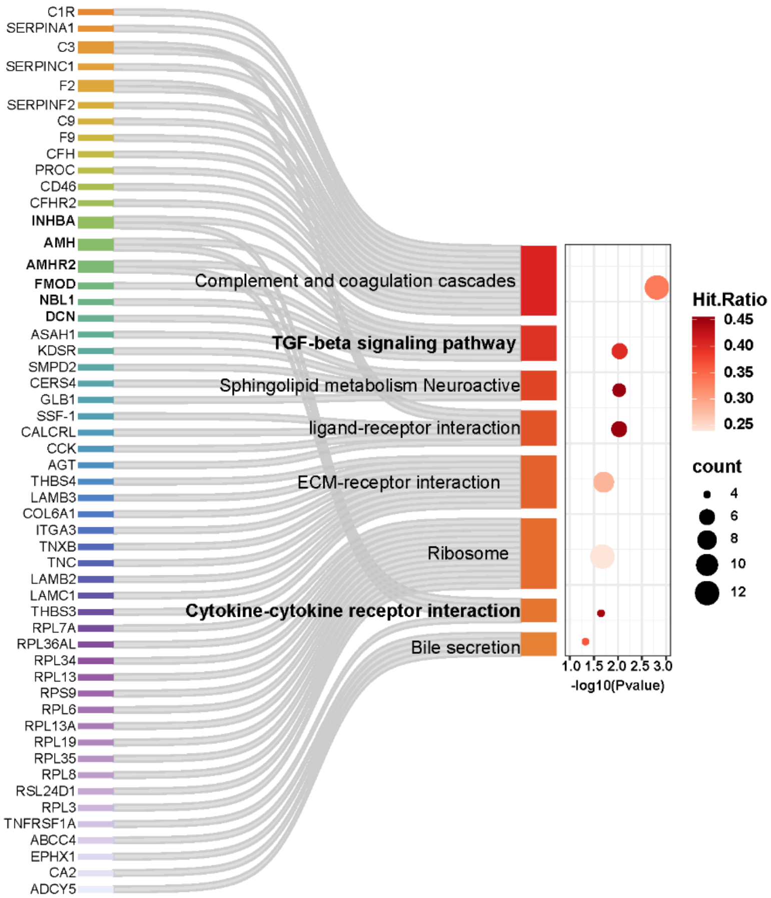

Fig. S12. Sankey plot showing DEPs were involved in each of the enriched pathways obtained via KEGG. The dot plot showed the ratio between DEPs and the total number of proteins in each enriched pathway (FDR-adjusted *P*≤ 0.05).

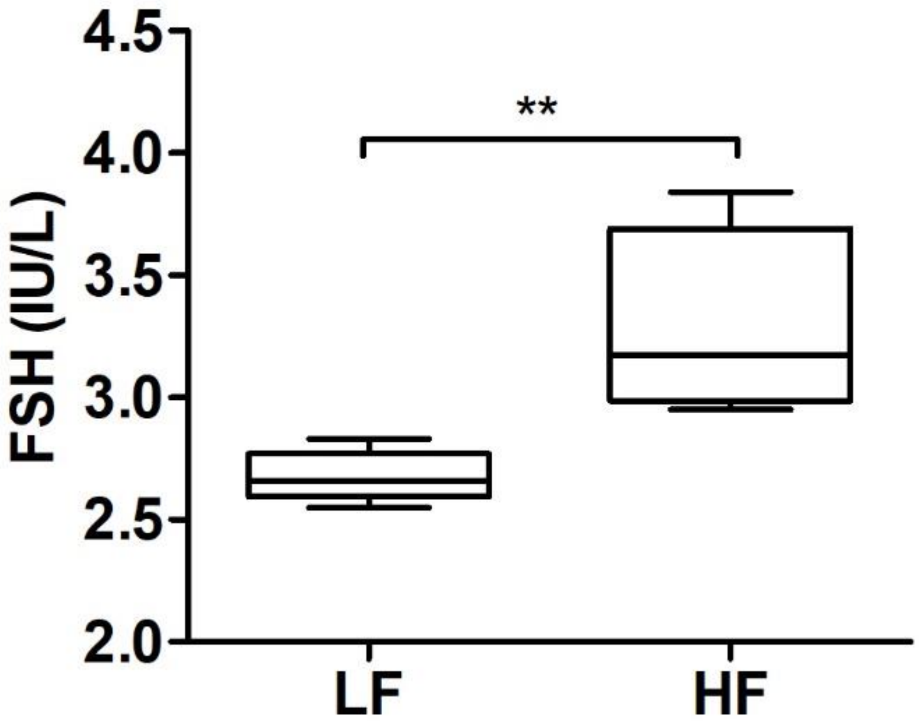

Fig. S13. Determination of serum FSH levels in both groups by ELISA. FSH, follicle stimulating hormone. The bars display mean ± SD. A two-tailed t test was used (**P* < 0.05; **P < 0.01; ***P < 0.001).

Tables S1 to S17

Table S1. Phenotypes of production traits in the genome-wide association analysis.

Sample	ID	group	Litter size of the first parity	Litter size of the second parity	Litter size of the third parity	Average litter size	lambing interval	*BMPR1B* genotype	*PAPPA* genotype
1	0005	HF	2	2	2	2.0	357	GG	TC
2	1527	HF	2	2	2	2.0	362	GG	CC
3	4918	HF	3	2	3	2.7	361	GG	CC
4	4922	HF	2	3	3	2.7	359	GG	CC
5	4962	HF	2	3	3	2.7	366	GG	CC
6	4963	HF	2	3	3	2.7	354	GG	TC
7	H10	LF	1	1	1	1.0	359	AA	TT
8	H1	LF	1	1	1	1.0	357	AA	CC
9	H2	LF	1	1	1	1.0	358	AA	CC
10	H3	LF	1	1	1	1.0	357	AA	CC
11	H4	LF	1	1	1	1.0	357	AA	CC
12	H5	LF	1	1	1	1.0	359	AA	CC
13	H6	LF	1	1	1	1.0	358	AA	CC
14	H7	LF	1	1	1	1.0	358	AA	CC
15	H8	LF	1	1	1	1.0	358	AA	CC
16	H9	LF	1	1	1	1.0	363	AA	TC
17	M10	HF	1	2	2	1.7	280	AG	TT
18	M11	HF	2	2	2	2.0	295	AG	TT
19	M12	HF	2	2	2	2.0	287	AG	TC
20	M13	HF	2	1	2	1.7	330	AG	TT
21	M14	HF	2	2	2	2.0	262	AG	TT
22	M15	HF	2	2	2	2.0	291	AG	TT
23	M16	HF	2	2	2	2.0	289	AG	TT
24	M17	HF	2	2	1	1.7	335	AG	TC
25	M18	LF	1	1	1	1.0	357	AG	CC
26	M19	HF	1	2	2	1.7	350	AG	CC
27	M1	HF	2	2	1	1.7	280	AG	TT
28	M20	HF	2	1	3	2.0	293	AG	TC
29	M21	LF	1	1	1	1.0	359	AG	CC
30	M22	HF	1	2	2	1.7	346	AG	TC
31	M23	HF	2	1	2	1.7	340	AG	TC
32	M24	LF	1	1	1	1.0	360	AG	CC
33	M25	HF	1	2	2	1.7	292	AG	CC
34	M26	HF	2	1	2	1.7	337	AG	TC
35	M27	LF	1	1	1	1.0	358	AA	CC
36	M28	LF	1	1	1	1.0	355	AA	CC
37	M29	LF	1	1	1	1.0	339	AA	TC

38	M2	HF	2	2	2	2.0	291	AG	TT
39	M30	LF	1	1	1	1.0	361	AA	CC
40	M31	LF	1	1	1	1.0	353	AA	CC
41	M32	LF	1	1	1	1.0	352	AA	TC
42	M33	LF	1	1	1	1.0	363	AA	TT
43	M34	LF	1	1	1	1.0	357	AA	TC
44	M35	LF	1	1	1	1.0	366	AA	CC
45	M36	HF	3	2	3	2.7	285	GG	TT
46	M37	HF	2	2	2	2.0	289	AG	TT
47	M3	HF	2	1	2	1.7	331	AG	TC
48	M4	HF	2	2	2	2.0	280	AG	TT
49	M5	HF	2	2	2	2.0	291	AG	TT
50	M6	HF	2	2	2	2.0	290	AG	TC
51	M7	HF	2	2	2	2.0	289	AG	TT
52	M8	HF	2	2	3	2.3	290	AG	TT
53	M9	HF	2	2	2	2.0	335	AG	CC

Table S2. Sequencing statistics for 31 high-fertility (HF) and 22 low-fertility (LF)Tibetan sheep.

				Before filtering		
**ID**	**group**	**total reads**	**total bases**	**Q20 bases**	**Q30 bases**	**GC content**
0005	HF	184.218906 M	27.632836 G	26.559064 G (96.114143%)	24.964234 G (90.342641%)	43.78%
1527	HF	181.138904 M	27.170836 G	26.195869 G (96.411715%)	24.686511 G (90.856650%)	44.24%
4918	HF	178.712138 M	26.806821 G	25.867828 G (96.497187%)	24.406235 G (91.044871%)	43.95%
4922	HF	176.082810 M	26.412422 G	25.439723 G (96.317268%)	23.960324 G (90.716121%)	43.73%
4962	HF	175.608954 M	26.341343 G	25.224282 G (95.759284%)	23.614469 G (89.647929%)	44.00%
4963	HF	177.859108 M	26.678866 G	25.606753 G (95.981412%)	24.016970 G (90.022452%)	44.24%
H1	LF	186.039722 M	27.905958 G	27.293540 G (97.805422%)	26.104057 G (93.542951%)	44.80%
H2	LF	178.631102 M	26.794665 G	26.173735 G (97.682634%)	25.013465 G (93.352406%)	44.77%
H3	LF	174.443184 M	26.166478 G	25.566228 G (97.706037%)	24.425339 G (93.345920%)	44.49%
H4	LF	176.903736 M	26.535560 G	25.885022 G (97.548427%)	24.663147 G (92.943759%)	44.24%
H5	LF	176.642578 M	26.496387 G	25.909725 G (97.785881%)	24.783178 G (93.534179%)	44.56%
H6	LF	174.393592 M	26.159039 G	25.593574 G (97.838357%)	24.502019 G (93.665596%)	44.39%
H7	LF	174.924806 M	26.238721 G	25.636841 G (97.706140%)	24.489815 G (93.334638%)	44.29%
H8	LF	175.763664 M	26.364550 G	25.750550 G (97.671115%)	24.596537 G (93.293978%)	44.44%
H9	LF	173.699530 M	26.054930 G	25.481132 G (97.797738%)	24.373673 G (93.547262%)	44.42%
H10	LF	175.663866 M	26.349580 G	25.776962 G (97.826844%)	24.663602 G (93.601500%)	44.29%
M1	HF	191.288640 M	28.693296 G	27.961555 G (97.449784%)	26.617663 G (92.766141%)	43.91%
M2	HF	178.923974 M	26.838596 G	26.176932 G (97.534655%)	24.971451 G (93.043060%)	44.41%
M3	HF	215.218042 M	32.282706 G	31.480539 G (97.515180%)	30.010956 G (92.962950%)	44.15%
M4	HF	175.040582 M	26.256087 G	25.599941 G (97.500973%)	24.405216 G (92.950697%)	44.35%
M5	HF	179.487996 M	26.923199 G	26.260002 G (97.536708%)	25.047814 G (93.034313%)	44.19%
M6	HF	176.863214 M	26.529482 G	26.015336 G (98.061981%)	24.971742 G (94.128268%)	44.32%
M7	HF	187.529796 M	28.129469 G	27.467714 G (97.647465%)	26.237443 G (93.273865%)	44.21%
M8	HF	177.972552 M	26.695883 G	26.062405 G (97.627059%)	24.900409 G (93.274341%)	44.12%
M9	HF	174.902558 M	26.235384 G	25.590521 G (97.542013%)	24.408689 G (93.037288%)	44.38%

M10	HF	174.621070 M	26.193161 G	25.543867 G (97.521132%)	24.353790 G (92.977669%)	44.18%
M11	HF	175.705156 M	26.355773 G	25.719494 G (97.585807%)	24.550536 G (93.150503%)	44.27%
M12	HF	208.777482 M	31.316622 G	30.515490 G (97.441830%)	29.067514 G (92.818163%)	44.22%
M13	HF	176.439260 M	26.465889 G	25.824539 G (97.576693%)	24.646686 G (93.126237%)	44.41%
M14	HF	178.651584 M	26.797738 G	26.173786 G (97.671625%)	25.018124 G (93.359090%)	44.01%
M15	HF	175.038952 M	26.255843 G	25.587784 G (97.455582%)	24.371837 G (92.824431%)	44.34%
M16	HF	175.728796 M	26.359319 G	25.636688 G (97.258537%)	24.335994 G (92.324060%)	44.16%
M17	HF	176.249932 M	26.437490 G	25.787669 G (97.542048%)	24.601107 G (93.053869%)	44.82%
M18	LF	177.481278 M	26.622192 G	25.986987 G (97.614002%)	24.800030 G (93.155479%)	44.32%
M19	HF	178.915094 M	26.837264 G	26.149392 G (97.436878%)	24.902736 G (92.791635%)	44.11%
M20	HF	176.820526 M	26.523079 G	25.840429 G (97.426204%)	24.602678 G (92.759509%)	44.15%
M21	LF	174.461096 M	26.169164 G	25.495563 G (97.425974%)	24.273590 G (92.756458%)	43.92%
M22	HF	174.789120 M	26.218368 G	25.592993 G (97.614746%)	24.428043 G (93.171484%)	43.93%
M23	HF	178.737748 M	26.810662 G	26.160946 G (97.576649%)	24.976460 G (93.158683%)	44.33%
M24	LF	174.059118 M	26.108868 G	25.481420 G (97.596803%)	24.317873 G (93.140283%)	44.25%
M25	HF	179.261638 M	26.889246 G	26.226666 G (97.535895%)	25.016584 G (93.035650%)	43.90%
M26	HF	193.200348 M	28.980052 G	28.316353 G (97.709806%)	27.052121 G (93.347385%)	44.12%
M27	LF	177.483190 M	26.622478 G	26.012942 G (97.710444%)	24.851671 G (93.348449%)	44.26%
M28	LF	175.103218 M	26.265483 G	25.662245 G (97.703306%)	24.492024 G (93.247948%)	44.20%
M29	LF	174.303492 M	26.145524 G	25.537028 G (97.672659%)	24.363812 G (93.185403%)	44.30%
M30	LF	185.037004 M	27.755551 G	27.073972 G (97.544350%)	25.793756 G (92.931883%)	44.18%
M31	LF	175.198622 M	26.279793 G	25.624987 G (97.508328%)	24.401093 G (92.851160%)	44.41%
M32	LF	176.115384 M	26.417308 G	25.805773 G (97.685099%)	24.633105 G (93.246084%)	43.98%
M33	LF	176.849090 M	26.527363 G	25.881204 G (97.564177%)	24.674783 G (93.016343%)	44.18%
M34	LF	176.695796 M	26.504369 G	25.882534 G (97.653839%)	24.696875 G (93.180390%)	44.55%
M35	LF	203.433056 M	30.514958 G	29.782806 G (97.600678%)	28.408170 G (93.095883%)	44.21%
M36	HF	177.857028 M	26.678554 G	26.060947 G (97.685005%)	24.882490 G (93.267760%)	44.39%
M37	HF	174.430470 M	26.164570 G	25.540842 G (97.616134%)	24.348295 G (93.058263%)	43.93%

After filtering

ID	group	total reads	total bases	Q20 bases	Q30 bases	GC content	reads passed filters
0005	HF	181.719780 M	27.241043 G	26.293650 G (96.522185%)	24.753557 G (90.868612%)	43.74%	98.64%
1527	HF	179.125010 M	26.845815 G	25.974109 G (96.752919%)	24.509042 G (91.295580%)	44.21%	98.89%
4918	HF	176.827502 M	26.506341 G	25.662652 G (96.817030%)	24.241667 G (91.456103%)	43.91%	98.95%
4922	HF	174.111434 M	26.098645 G	25.226909 G (96.659842%)	23.790904 G (91.157622%)	43.70%	98.88%
4962	HF	172.896062 M	25.909442 G	24.933212 G (96.232147%)	23.384867 G (90.256157%)	43.95%	98.46%
4963	HF	175.452200 M	26.295498 G	25.346491 G (96.390990%)	23.810186 G (90.548525%)	44.21%	98.65%
H1	LF	185.095508 M	27.743310 G	27.182687 G (97.979252%)	26.012648 G (93.761878%)	44.75%	99.49%
H2	LF	177.619358 M	26.621860 G	26.056406 G (97.875979%)	24.917309 G (93.597179%)	44.73%	99.43%
H3	LF	173.517512 M	26.008173 G	25.458459 G (97.886378%)	24.336851 G (93.573858%)	44.45%	99.47%
H4	LF	175.962788 M	26.374485 G	25.775537 G (97.729063%)	24.573592 G (93.171834%)	44.20%	99.47%
H5	LF	175.754884 M	26.339139 G	25.803301 G (97.965620%)	24.695883 G (93.761165%)	44.51%	99.50%
H6	LF	173.528366 M	26.010364 G	25.492557 G (98.009228%)	24.418749 G (93.880843%)	44.34%	99.50%
H7	LF	174.000680 M	26.085058 G	25.531649 G (97.878443%)	24.403054 G (93.551849%)	44.26%	99.47%
H8	LF	174.802808 M	26.200659 G	25.639418 G (97.857915%)	24.505438 G (93.529856%)	44.40%	99.45%
H9	LF	172.832542 M	25.902761 G	25.378212 G (97.974931%)	24.289213 G (93.770749%)	44.38%	99.50%
H10	LF	174.782672 M	26.198112 G	25.673769 G (97.998548%)	24.578516 G (93.817892%)	44.25%	99.50%
M1	HF	190.110180 M	28.502773 G	27.831556 G (97.645081%)	26.511366 G (93.013288%)	43.88%	99.38%
M2	HF	177.830174 M	26.653258 G	26.052419 G (97.745723%)	24.870254 G (93.310373%)	44.37%	99.39%
M3	HF	213.878780 M	32.063828 G	31.331926 G (97.717358%)	29.889465 G (93.218641%)	44.11%	99.38%
M4	HF	173.923054 M	26.071700 G	25.474799 G (97.710540%)	24.303071 G (93.216286%)	44.31%	99.36%
M5	HF	178.364636 M	26.738791 G	26.135058 G (97.742109%)	24.945908 G (93.294825%)	44.15%	99.37%
M6	HF	176.068100 M	26.394414 G	25.921291 G (98.207490%)	24.894027 G (94.315514%)	44.29%	99.55%
M7	HF	186.446386 M	27.951357 G	27.346698 G (97.836743%)	26.138170 G (93.513062%)	44.17%	99.42%
M8	HF	176.916894 M	26.520997 G	25.943820 G (97.823699%)	24.803471 G (93.523900%)	44.09%	99.41%
M9	HF	173.812848 M	26.058990 G	25.470321 G (97.741012%)	24.310334 G (93.289625%)	44.34%	99.38%

M10	HF	173.562300 M	26.017157 G	25.424598 G (97.722430%)	24.256551 G (93.232901%)	44.14%	99.39%
M11	HF	174.640716 M	26.180825 G	25.600583 G (97.783715%)	24.453228 G (93.401287%)	44.23%	99.39%
M12	HF	207.409012 M	31.092340 G	30.363749 G (97.656688%)	28.943812 G (93.089848%)	44.18%	99.34%
M13	HF	175.394590 M	26.290709 G	25.705550 G (97.774273%)	24.549279 G (93.376252%)	44.37%	99.41%
M14	HF	177.611428 M	26.626600 G	26.057002 G (97.860795%)	24.922287 G (93.599209%)	43.97%	99.42%
M15	HF	173.940590 M	26.073102 G	25.464150 G (97.664443%)	24.271111 G (93.088698%)	44.30%	99.37%
M16	HF	174.575178 M	26.172800 G	25.509773 G (97.466734%)	24.232624 G (92.587051%)	44.13%	99.34%
M17	HF	175.150816 M	26.255334 G	25.664346 G (97.749074%)	24.500248 G (93.315315%)	44.78%	99.38%
M18	LF	176.444878 M	26.451468 G	25.870588 G (97.803979%)	24.704448 G (93.395377%)	44.28%	99.42%
M19	HF	177.776500 M	26.650896 G	26.022621 G (97.642573%)	24.799230 G (93.052143%)	44.07%	99.36%
M20	HF	175.693314 M	26.337318 G	25.714320 G (97.634545%)	24.499853 G (93.023340%)	44.11%	99.36%
M21	LF	173.358346 M	25.985901 G	25.371551 G (97.635834%)	24.172596 G (93.021966%)	43.87%	99.37%
M22	HF	173.774052 M	26.052263 G	25.479732 G (97.802376%)	24.335068 G (93.408654%)	43.89%	99.42%
M23	HF	177.609044 M	26.625365 G	26.034954 G (97.782525%)	24.873389 G (93.419897%)	44.29%	99.37%
M24	LF	173.001992 M	25.935543 G	25.363086 G (97.792768%)	24.220825 G (93.388539%)	44.21%	99.39%
M25	HF	178.145496 M	26.706711 G	26.102261 G (97.736713%)	24.914810 G (93.290449%)	43.86%	99.38%
M26	HF	192.157292 M	28.805832 G	28.197383 G (97.887758%)	26.953966 G (93.571211%)	44.08%	99.46%
M27	LF	176.501674 M	26.459174 G	25.901100 G (97.890814%)	24.759494 G (93.576219%)	44.22%	99.45%
M28	LF	174.190664 M	26.115772 G	25.559013 G (97.868111%)	24.406399 G (93.454633%)	44.17%	99.48%
M29	LF	173.383522 M	25.992757 G	25.432062 G (97.842877%)	24.276931 G (93.398831%)	44.26%	99.47%
M30	LF	183.968150 M	27.580536 G	26.954015 G (97.728394%)	25.695076 G (93.163802%)	44.14%	99.42%
M31	LF	174.152902 M	26.105578 G	25.506067 G (97.703514%)	24.303229 G (93.095922%)	44.36%	99.40%
M32	LF	175.146846 M	26.254582 G	25.694510 G (97.866765%)	24.541138 G (93.473734%)	43.94%	99.45%
M33	LF	175.836838 M	26.356930 G	25.765147 G (97.754735%)	24.579606 G (93.256709%)	44.13%	99.43%
M34	LF	175.739418 M	26.344989 G	25.773463 G (97.830608%)	24.606971 G (93.402851%)	44.50%	99.46%
M35	LF	202.279842 M	30.319434 G	29.649953 G (97.791910%)	28.299074 G (93.336421%)	44.16%	99.43%
M36	HF	176.913706 M	26.520093 G	25.952758 G (97.860737%)	24.793222 G (93.488443%)	44.34%	99.47%
M37	HF	173.472736 M	26.008198 G	25.433200 G (97.789165%)	24.259371 G (93.275863%)	43.89%	99.45%

Table S3. The genome-wide significant SNPs association with the litter size and the lambing interval, respectively.

Litter size							Lambing interval						
**Chr**	**SNP site**	**-log$_{10}$(*P*)**	**Ref**	**Alt**	**Feature**	**Gene**	**Chr**	**SNP site**	**-log$_{10}$(*P*)**	**Ref**	**Alt**	**Feature**	**Gene**
6	30050621	11.22	T	C	missense_variant	*BMPR1B*	2	7400929	9.28	A	G	intron_variant	*PAPPA*
6	30073797	9.85	T	G	intron_variant	*BMPR1B*	2	7393090	7.82	A	G	intron_variant	*PAPPA*
6	30055664	9.49	G	A	intron_variant	*BMPR1B*	2	7400663	7.45	A	T	intron_variant	*PAPPA*
6	30072510	8.01	G	C	intron_variant	*BMPR1B*	2	7397119	7.38	T	C	intron_variant	*PAPPA*
6	26875618	7.98	A	G	intergenic_region	*RAP1GDS1-LOC114115502*	2	7393393	6.92	G	T	intron_variant	*PAPPA*
6	30075710	7.87	T	C	intron_variant	*BMPR1B*	2	7397217	6.87	A	G	intron_variant	*PAPPA*
6	24766679	7.65	C	A	intergenic_region	*EMCN-LOC105612761*	2	39024832	6.64	G	A	intergenic_region	*ADRA1A-DPYSL2*
6	30024064	7.62	T	C	upstream_variant	*LOC121819816*	2	7394074	6.63	C	T	intron_variant	*PAPPA*
6	24757607	7.29	C	T	intergenic_region	*EMCN-LOC105612761*	2	213388660	6.16	C	A	intron_variant	*ERBB4*
6	30050869	7.17	C	T	intron_variant	*BMPR1B*	2	7392811	6.13	C	T	intron_variant	*PAPPA*
6	30070718	7.15	C	G	intron_variant	*BMPR1B*	2	213387546	6.12	C	T	intron_variant	*ERBB4*
6	30019006	7.14	G	A	3_UTR_variant	*UNC5C*	2	54393553	6.09	C	T	intergenic_region	*TRNAG-CCC-LOC121818504*
6	24767095	6.94	T	C	intergenic_region	*EMCN-LOC105612761*	2	1385091	6.03	A	C	intergenic_region	*MYT1L-LOC121818568*
6	30037354	6.87	G	T	intron_variant	*BMPR1B*	2	242304497	6.01	T	C	intron_variant	*STPG1*
6	24766301	6.79	C	T	intergenic_region	*EMCN-LOC105612761*	2	149908393	5.88	A	G	intron_variant	*LOC101109941*
6	24774891	6.79	C	T	intergenic_region	*EMCN-LOC105612761*	2	158604807	5.84	A	G	intergenic_region	*TRNAG-CCC-LOC114112869*
6	30055737	6.76	G	A	intron_variant	*BMPR1B*	2	93862249	5.82	A	G	intergenic_region	*LOC101109243-LOC780442*
6	30053923	6.69	T	A	intron_variant	*BMPR1B*	2	216440439	5.71	G	T	intergenic_region	*LOC114113297-BARD1*
6	24766943	6.65	T	C	intergenic_region	*EMCN-LOC105612761*	2	1380936	5.70	T	C	intergenic_region	*MYT1L-LOC121818568*

6	24780740	6.65	A	G	intergenic_region	*EMCN-LOC105612761*	2	213388264	5.65	T	C	intron_variant	*ERBB4*
6	24758699	6.64	C	T	intergenic_region	*EMCN-LOC105612761*	2	158666978	5.60	G	T	intergenic_region	*TRNAG-CCC-LOC114112869*
6	30014573	6.54	G	T	downstream_variant	*LOC121819816*	2	7412471	5.55	T	C	intron_variant	*PAPPA*
6	24781054	6.54	G	T	intergenic_region	*EMCN-LOC105612761*	2	158664212	5.51	G	A	intergenic_region	*TRNAG-CCC-LOC114112869*
6	24756956	6.51	T	C	intergenic_region	*EMCN-LOC105612761*	2	122999018	5.50	A	C	intergenic_region	*TRNAC-GCA-LOC114113605*
6	26916382	6.51	G	A	intergenic_region	*LOC114115502-STPG2*	2	7393423	5.48	G	A	intron_variant	*PAPPA*
6	30054440	6.49	T	C	intron_variant	*BMPR1B*	2	126936868	5.47	A	G	downstream_variant	*LOC114113288*
6	30066368	6.43	A	G	intron_variant	*BMPR1B*	2	106112302	5.45	C	T	intron_variant	*GLRA3*
6	24771419	6.40	G	A	intergenic_region	*EMCN-LOC105612761*	2	166970943	5.41	C	T	intron_variant	*ARHGAP15*
6	29330636	6.29	A	G	intergenic_region	*PDHA2-UNC5C*	2	166959202	5.39	C	T	intron_variant	*ARHGAP15*
6	116092691	6.21	A	G	intron_variant	*LRPAP1*	2	54459101	5.35	A	C	intergenic_region	*TRNAG-CCC-LOC121818504*
6	29985599	6.19	C	T	intron_variant	*UNC5C*	2	32730241	5.35	C	G	intron_variant	*LOC105607140*
6	24768303	6.17	A	G	intergenic_region	*EMCN-LOC105612761*	2	32730609	5.35	C	T	upstream_variant	*LOC114113379*
6	95431923	6.16	G	C	intron_variant	*ANTXR2*	2	7392440	5.31	A	G	intron_variant	*PAPPA*
6	30024899	6.12	C	G	upstream_variant	*LOC121819816*	2	126946040	5.31	G	A	intergenic_region	*LOC114113288-LOC105608906*
6	28541763	6.12	T	C	intergenic_region	*LOC121819815-LOC121819867*	2	1376801	5.31	G	C	intergenic_region	*MYT1L-LOC121818568*
6	72397956	6.05	G	T	intron_variant	*CRACD*	2	31368641	5.31	G	A	intron_variant	*AOPEP*
6	29953443	6.03	T	G	intron_variant	*UNC5C*	2	54401853	5.25	G	A	intergenic_region	*TRNAG-CCC-LOC121818504*
6	26874053	5.99	C	T	intergenic_region	*RAP1GDS1-LOC114115502*	2	193590200	5.25	G	A	intron_variant	*MYO1B*
6	24765040	5.98	A	G	intergenic_region	*EMCN-LOC105612761*	2	166554762	5.25	C	T	intron_variant	*ARHGAP15*

6	24765391	5.98	G	T	intergenic_region	*EMCN-LOC105612761*	2	104932268	5.24	A	G	intron_variant	*XKR6*
6	24780971	5.98	A	T	intergenic_region	*EMCN-LOC105612761*	2	7399572	5.21	A	G	synonymous_variant	*PAPPA*
6	26871621	5.98	G	A	intergenic_region	*RAP1GDS1-LOC114115502*	2	166654534	5.19	C	G	intron_variant	*ARHGAP15*
6	34983624	5.90	C	T	intron_variant	*CCSER1*	2	32708485	5.17	T	C	intergenic_region	*TRNAA-AGC-LOC105607140*
6	30027418	5.84	T	C	downstream_variant	*BMPR1B*	2	7393611	5.17	T	C	intron_variant	*PAPPA*
6	30063912	5.84	T	G	intron_variant	*BMPR1B*	2	54396187	5.15	C	T	intergenic_region	*TRNAG-CCC-LOC121818504*
6	37063293	5.83	C	T	intergenic_region	*HERC6-LOC114115227*	2	7396330	5.15	C	T	intron_variant	*PAPPA*
6	72398078	5.82	C	T	intron_variant	*CRACD*	2	83725192	5.13	C	T	intron_variant	*TTC39B*
6	24765100	5.81	A	G	intergenic_region	*EMCN-LOC105612761*	2	12714658	5.08	C	T	upstream_variant	*MUSK*
6	30065263	5.81	A	G	intron_variant	*BMPR1B*	2	144263139	5.08	A	G	intron_variant	*SCN2A*
6	26378604	5.80	C	T	intergenic_region	*LOC105612638-LOC101123379*	2	83625426	5.06	C	T	intergenic_region	*FREM1-TTC39B*
6	26870422	5.80	C	T	intergenic_region	*RAP1GDS1-LOC114115502*	2	148286788	5.06	T	C	intergenic_region	*SLC4A10-TBR1*
6	29975058	5.78	T	A	intron_variant	*UNC5C*							
6	26875785	5.76	C	T	intergenic_region	*RAP1GDS1-LOC114115502*							
6	29950820	5.76	C	T	intron_variant	*UNC5C*							
6	29983535	5.76	C	A	intron_variant	*UNC5C*							
6	30020513	5.76	G	A	downstream_variant	*UNC5C*							
6	30026228	5.76	A	G	upstream_variant	*LOC121819816*							
6	30030903	5.75	T	C	3_UTR_variant	*BMPR1B*							
6	30055993	5.75	C	T	intron_variant	*BMPR1B*							
6	34984310	5.74	T	A	intron_variant	*CCSER1*							
6	30003364	5.74	A	G	intron_variant	*UNC5C*							

6	48132850	5.71	T	G	intergenic_region	*LOC114115229-LOC101107099*
6	30048543	5.67	C	T	intron_variant	*BMPR1B*
6	24784267	5.64	T	C	intergenic_region	*EMCN-LOC105612761*
6	31865796	5.64	G	A	intron_variant	*GRID2*
6	93784406	5.64	G	A	intron_variant	*FRAS1*
6	30002691	5.63	T	G	intron_variant	*UNC5C*
6	29950388	5.58	C	T	intron_variant	*UNC5C*
6	30002417	5.58	C	T	intron_variant	*UNC5C*
6	116110903	5.57	G	A	intron_variant	*DOK7*
6	26892849	5.57	G	T	upstream_variant	*LOC114115502*
6	31567617	5.55	A	C	intron_variant	*GRID2*
6	30048259	5.53	T	G	intron_variant	*BMPR1B*
6	30170543	5.52	G	T	intron_variant	*BMPR1B*
6	62659104	5.52	G	A	intergenic_region	*GRXCR1-LOC101111286*
6	101563956	5.51	A	G	intron_variant	*MAPK10*
6	21032455	5.50	T	C	intergenic_region	*LOC121819813-CXXC4*
6	29989674	5.49	T	C	intron_variant	*UNC5C*
6	30055702	5.47	G	A	intron_variant	*BMPR1B*
6	24756132	5.47	G	T	intergenic_region	*EMCN-LOC105612761*
6	24765667	5.47	G	T	intergenic_region	*EMCN-LOC105612761*
6	24774955	5.47	G	A	intergenic_region	*EMCN-LOC105612761*
6	24780968	5.47	A	T	intergenic_region	*EMCN-LOC105612761*
6	25194506	5.47	T	C	upstream_variant	*DAPP1*
6	29330399	5.47	C	T	intergenic_region	*PDHA2-UNC5C*

6	30063743	5.41	C	T	intron_variant	*BMPR1B*
6	29955615	5.37	G	A	intron_variant	*UNC5C*
6	35143851	5.36	G	T	intron_variant	*CCSER1*
6	30026109	5.34	C	A	upstream_variant	*LOC121819816*
6	30003166	5.34	G	A	intron_variant	*UNC5C*
6	45954878	5.33	A	G	intergenic_region	*SEL1L3-LOC105608876*
6	24782726	5.32	C	T	intergenic_region	*EMCN-LOC105612761*
6	24783240	5.32	C	A	intergenic_region	*EMCN-LOC105612761*
6	25199411	5.32	C	T	intergenic_region	*DAPP1-C6H4orf54*
6	26376250	5.32	T	C	upstream_variant	*LOC105612638*
6	26382851	5.32	T	A	intergenic_region	*LOC105612638-LOC101123379*
6	30025886	5.31	T	A	upstream_variant	*LOC121819816*
6	38547749	5.31	C	T	intergenic_region	*LCORL-LOC101104580*
6	24759433	5.29	C	T	intergenic_region	*EMCN-LOC105612761*
6	34984736	5.27	A	G	intron_variant	*CCSER1*
6	29966862	5.25	G	A	intron_variant	*UNC5C*
6	24796187	5.25	G	A	intergenic_region	*EMCN-LOC105612761*
6	30063846	5.25	C	T	intron_variant	*BMPR1B*
6	95388216	5.24	C	T	intron_variant	*ANTXR2*
6	24785673	5.23	A	G	intergenic_region	*EMCN-LOC105612761*
6	33802438	5.23	A	T	intron_variant	*CCSER1*
6	53257064	5.21	A	G	intergenic_region	*LOC101107868-TRNAC-GCA*
6	30057707	5.21	G	C	intron_variant	*BMPR1B*

6	30065207	5.21	G	A	intron_variant	*BMPR1B*
6	30065248	5.21	A	G	intron_variant	*BMPR1B*
6	25207851	5.20	A	T	intergenic_region	*DAPP1-C6H4orf54*
6	27888849	5.19	C	T	intergenic_region	*LOC121819815-LOC121819867*
6	24706595	5.19	G	A	intergenic_region	*EMCN-LOC105612761*
6	25236150	5.19	G	T	intergenic_region	*DAPP1-C6H4orf54*
6	25236200	5.19	C	T	intergenic_region	*DAPP1-C6H4orf54*
6	31047474	5.19	G	T	intron_variant	*SMARCAD1*
6	33780927	5.19	G	A	intron_variant	*CCSER1*
6	25195264	5.17	A	G	upstream_variant	*DAPP1*
6	34992759	5.17	G	A	intron_variant	*CCSER1*
6	95456590	5.14	A	G	intron_variant	*ANTXR2*
6	105535198	5.13	T	G	intron_variant	*TMEM128*
6	93654317	5.11	C	T	intron_variant	*FRAS1*
6	30640342	5.10	C	T	intron_variant	*PDLIM5*
6	30053720	5.09	C	G	intron_variant	*BMPR1B*
6	24932869	5.08	A	G	intergenic_region	*DDIT4L-H2AZ1*
6	31052800	5.08	A	T	intron_variant	*SMARCAD1*
6	25186929	5.07	T	C	intron_variant	*DAPP1*
6	25225505	5.07	A	T	intergenic_region	*DAPP1-C6H4orf54*
6	25225520	5.07	T	A	intergenic_region	*DAPP1-C6H4orf54*
6	24780828	5.06	G	A	intergenic_region	*EMCN-LOC105612761*
6	29950454	5.05	G	A	intron_variant	*UNC5C*
6	30045239	5.04	G	A	intron_variant	*BMPR1B*

6	24765297	5.04	C	T	intergenic_region	*EMCN-LOC105612761*
6	93785708	5.04	T	C	intron_variant	*FRAS1*
6	36034531	5.03	T	C	intergenic_region	*LOC121819880-GPRIN3*
6	25191998	5.03	C	G	intron_variant	*DAPP1*
6	25193645	5.03	C	T	upstream_variant	*DAPP1*
6	25199623	5.03	A	C	intergenic_region	*DAPP1-C6H4orf54*
6	26375860	5.03	G	A	upstream_variant	*LOC105612638*
6	34992737	5.03	A	G	intron_variant	*CCSER1*
6	37099452	5.03	C	G	intergenic_region	*LOC114115227-PPM1K*
6	108133086	5.03	G	T	intergenic_region	*TRNAW-CCA-LOC114115439*
6	116094250	5.03	C	T	downstream_variant	*LRPAP1*
6	72389057	5.01	A	G	intron_variant	*CRACD*
6	36021578	5.01	A	G	intergenic_region	*LOC121819880-GPRIN3*
6	92786503	5.01	C	T	intergenic_region	*CCNG2-LOC101107023*
6	107331079	5.01	T	C	intergenic_region	*TRNAW-CCA-LOC114115439*

Notes: The position of all regions were located on the genomes of ARS-UI_Ramb_v2.0 (GCF_016772045.1). Chr: Chromosome; Ref: Reference allele; Alt: Alternative allele.

Table S4. Statistical information on production trait phenotypes and *PAPPA* and *BMPR1B* genotypes in large populations (n=1,130)

Sample	ID	Litter size	Lambing interval	*BMPR1B*	*PAPPA*
1	D101	1	368	A/A	C/C
2	D107	1	368	A/A	C/C
3	D119	1	367	A/A	C/C
4	D120	1	367	A/A	C/C
5	D122	1	367	A/A	C/C
6	D128	1	367	A/A	C/C
7	D129	1	367	A/A	C/C
8	D138	1	367	A/A	C/C
9	D139	1	366	A/A	C/C
10	D141	1	366	A/A	C/C
11	D150	1	366	A/A	C/C
12	D153	1	366	A/A	C/C
13	D154	1	366	A/A	C/C
14	D155	1	366	A/A	C/C
15	D157	1	366	A/A	C/C
16	D166	1	366	A/A	C/C
17	D17	1	366	A/A	C/C
18	D170	1	365	A/A	C/C
19	D171	1	365	A/A	C/C
20	D172	1	365	A/A	C/C
21	D173	1	365	A/A	C/C
22	D176	1	365	A/A	C/C
23	D189	1	365	A/A	C/C
24	D190	1	365	A/A	C/C
25	D191	1	365	A/A	C/C
26	D192	1	364	A/A	C/C
27	D194	1	364	A/A	C/C
28	D195	1	364	A/A	C/C
29	D2	1	364	A/A	C/C
30	D202	1	364	A/A	C/C
31	D203	1	364	A/A	C/C
32	D211	1	364	A/A	C/C
33	D212	1	363	A/A	C/C
34	D214	1	363	A/A	C/C
35	D215	1	363	A/A	C/C
36	D216	1	363	A/A	C/C
37	D229	1	363	A/A	C/C
38	D232	1	363	A/A	C/C
39	D233	1	363	A/A	C/C

40	D242	1	363	A/A	C/C
41	D243	1	363	A/A	C/C
42	D247	1	363	A/A	C/C
43	D248	1	362	A/A	C/C
44	D250	1	362	A/A	C/C
45	D252	1	362	A/A	C/C
46	D254	1	362	A/A	C/C
47	D255	1	362	A/A	C/C
48	D256	1	362	A/A	C/C
49	D258	1	362	A/A	C/C
50	D262	1	362	A/A	C/C
51	D267	1	361	A/A	C/C
52	D268	1	361	A/A	C/C
53	D269	1	361	A/A	C/C
54	D272	1	361	A/A	C/C
55	D273	1	361	A/A	C/C
56	D274	1	361	A/A	C/C
57	D279	1	361	A/A	C/C
58	D280	1	361	A/A	C/C
59	D283	1	361	A/A	C/C
60	D285	1	361	A/A	C/C
61	D286	1	361	A/A	C/C
62	D289	1	360	A/A	C/C
63	D290	1	360	A/A	C/C
64	D293	1	360	A/A	C/C
65	D294	1	360	A/A	C/C
66	D295	1	360	A/A	C/C
67	D298	1	360	A/A	C/C
68	D302	1	360	A/A	C/C
69	D303	1	360	A/A	C/C
70	D306	1	360	A/A	C/C
71	D31	1	360	A/A	C/C
72	D310	1	360	A/A	C/C
73	D312	1	360	A/A	C/C
74	D314	1	360	A/A	C/C
75	D317	1	360	A/A	C/C
76	D321	1	360	A/A	C/C
77	D323	1	360	A/A	C/C
78	D324	1	360	A/A	C/C
79	D325	1	360	A/A	C/C
80	D327	1	360	A/A	C/C
81	D328	1	359	A/A	C/C
82	D33	1	359	A/A	C/C

83	D330	1	359	A/A	C/C
84	D336	1	359	A/A	C/C
85	D337	1	359	A/A	C/C
86	D34	1	359	A/A	C/C
87	D342	1	359	A/A	C/C
88	D343	1	359	A/A	C/C
89	D346	1	359	A/A	C/C
90	D347	1	359	A/A	C/C
91	D35	1	359	A/A	C/C
92	D352	1	359	A/A	C/C
93	D353	1	359	A/A	C/C
94	D356	1	358	A/A	C/C
95	D36	1	358	A/A	C/C
96	D360	1	358	A/A	C/C
97	D362	1	358	A/A	C/C
98	D368	1	358	A/A	C/C
99	D369	1	358	A/A	C/C
100	D370	1	358	A/A	C/C
101	D372	1	358	A/A	C/C
102	D379	1	358	A/A	C/C
103	D380	1	358	A/A	C/C
104	D381	1	358	A/A	C/C
105	D383	1	358	A/A	C/C
106	D384	1	358	A/A	C/C
107	D39	1	358	A/A	C/C
108	D398	1	357	A/A	C/C
109	D399	1	357	A/A	C/C
110	D40	1	357	A/A	C/C
111	D404	1	357	A/A	C/C
112	D405	1	357	A/A	C/C
113	D407	1	357	A/A	C/C
114	D411	1	357	A/A	C/C
115	D413	1	357	A/A	C/C
116	D415	1	357	A/A	C/C
117	D416	1	357	A/A	C/C
118	D417	1	357	A/A	C/C
119	D418	1	357	A/A	C/C
120	D42	1	356	A/A	C/C
121	D425	1	356	A/A	C/C
122	D426	1	356	A/A	C/C
123	D434	1	356	A/A	C/C
124	D436	1	356	A/A	C/C
125	D437	1	356	A/A	C/C

126	D47	1	356	A/A	C/C
127	D52	1	356	A/A	C/C
128	D58	1	356	A/A	C/C
129	D59	1	356	A/A	C/C
130	D6	1	356	A/A	C/C
131	D61	1	356	A/A	C/C
132	D67	1	356	A/A	C/C
133	D73	1	356	A/A	C/C
134	D77	1	356	A/A	C/C
135	D82	1	356	A/A	C/C
136	D84	1	356	A/A	C/C
137	D87	1	356	A/A	C/C
138	D88	1	355	A/A	C/C
139	D89	1	355	A/A	C/C
140	D9	1	355	A/A	C/C
141	D90	1	355	A/A	C/C
142	D94	1	355	A/A	C/C
143	D95	1	355	A/A	C/C
144	D97	1	355	A/A	C/C
145	D98	1	355	A/A	C/C
146	LD1	1	355	A/A	C/C
147	LD101	1	355	A/A	C/C
148	LD105	1	355	A/A	C/C
149	LD108	1	355	A/A	C/C
150	LD111	1	355	A/A	C/C
151	LD121	1	355	A/A	C/C
152	LD132	1	355	A/A	C/C
153	LD135	1	355	A/A	C/C
154	LD136	1	355	A/A	C/C
155	LD138	1	355	A/A	C/C
156	LD158	1	355	A/A	C/C
157	LD164	1	355	A/A	C/C
158	LD165	1	355	A/A	C/C
159	LD167	1	355	A/A	C/C
160	LD20	1	355	A/A	C/C
161	LD28	1	355	A/A	C/C
162	LD30	1	355	A/A	C/C
163	LD31	1	355	A/A	C/C
164	LD48	1	355	A/A	C/C
165	LD54	1	355	A/A	C/C
166	LD55	1	354	A/A	C/C
167	LD61	1	354	A/A	C/C
168	LD64	1	354	A/A	C/C

169	LD72	1	354	A/A	C/C
170	LD73	1	354	A/A	C/C
171	LD79	1	354	A/A	C/C
172	LD81	1	354	A/A	C/C
173	LD92	1	354	A/A	C/C
174	LD96	1	354	A/A	C/C
175	LD98	1	354	A/A	C/C
176	LD99	1	354	A/A	C/C
177	S106	1	354	A/A	C/C
178	S107	1	354	A/A	C/C
179	S108	1	354	A/A	C/C
180	S109	1	354	A/A	C/C
181	S11	1	354	A/A	C/C
182	S112	1	354	A/A	C/C
183	S12	1	354	A/A	C/C
184	S121	1	354	A/A	C/C
185	S124	1	354	A/A	C/C
186	S129	1	354	A/A	C/C
187	S13	1	354	A/A	C/C
188	S132	1	354	A/A	C/C
189	S133	1	354	A/A	C/C
190	S135	1	354	A/A	C/C
191	S139	1	354	A/A	C/C
192	S2	1	353	A/A	C/C
193	S20	1	353	A/A	C/C
194	S22	1	353	A/A	C/C
195	S25	1	353	A/A	C/C
196	S26	1	353	A/A	C/C
197	S27	1	353	A/A	C/C
198	S28	1	353	A/A	C/C
199	S29	1	353	A/A	C/C
200	S30	1	353	A/A	C/C
201	S31	1	353	A/A	C/C
202	S33	1	353	A/A	C/C
203	S40	1	353	A/A	C/C
204	S42	1	353	A/A	C/C
205	S44	1	353	A/A	C/C
206	S45	1	353	A/A	C/C
207	S53	1	353	A/A	C/C
208	S60	1	353	A/A	C/C
209	S61	1	353	A/A	C/C
210	S64	1	353	A/A	C/C
211	S65	1	353	A/A	C/C

212	S68	1	353	A/A	C/C
213	S71	1	353	A/A	C/C
214	S72	1	353	A/A	C/C
215	S81	1	353	A/A	C/C
216	S85	1	353	A/A	C/C
217	S87	1	353	A/A	C/C
218	S89	1	353	A/A	C/C
219	S9	1	353	A/A	C/C
220	S93	1	353	A/A	C/C
221	S95	1	353	A/A	C/C
222	S96	1	353	A/A	C/C
223	S98	1	352	A/A	C/C
224	S99	1	352	A/A	C/C
225	WJ104	1	352	A/A	C/C
226	WJ108	1	352	A/A	C/C
227	WJ113	1	352	A/A	C/C
228	WJ114	1	352	A/A	C/C
229	WJ115	1	352	A/A	C/C
230	WJ117	1	352	A/A	C/C
231	WJ12	1	352	A/A	C/C
232	WJ120	1	352	A/A	C/C
233	WJ122	1	352	A/A	C/C
234	WJ125	1	352	A/A	C/C
235	WJ126	1	352	A/A	C/C
236	WJ128	1	352	A/A	C/C
237	WJ130	1	352	A/A	C/C
238	WJ132	1	352	A/A	C/C
239	WJ133	1	352	A/A	C/C
240	WJ134	1	351	A/A	C/C
241	WJ135	1	351	A/A	C/C
242	WJ14	1	351	A/A	C/C
243	WJ141	1	351	A/A	C/C
244	WJ143	1	351	A/A	C/C
245	WJ145	1	351	A/A	C/C
246	WJ146	1	351	A/A	C/C
247	WJ149	1	351	A/A	C/C
248	WJ15	1	351	A/A	C/C
249	WJ151	1	351	A/A	C/C
250	WJ152	1	351	A/A	C/C
251	WJ157	1	351	A/A	C/C
252	WJ158	1	351	A/A	C/C
253	WJ159	1	351	A/A	C/C
254	WJ163	1	351	A/A	C/C

255	WJ164	1	351	A/A	C/C
256	WJ168	1	351	A/A	C/C
257	WJ17	1	351	A/A	C/C
258	WJ170	1	351	A/A	C/C
259	WJ175	1	351	A/A	C/C
260	WJ18	1	351	A/A	C/C
261	WJ182	1	351	A/A	C/C
262	WJ183	1	351	A/A	C/C
263	WJ186	1	351	A/A	C/C
264	WJ189	1	351	A/A	C/C
265	WJ190	1	351	A/A	C/C
266	WJ191	1	351	A/A	C/C
267	WJ192	1	351	A/A	C/C
268	WJ198	1	351	A/A	C/C
269	WJ20	1	351	A/A	C/C
270	WJ203	1	351	A/A	C/C
271	WJ204	1	351	A/A	C/C
272	WJ210	1	351	A/A	C/C
273	WJ211	1	351	A/A	C/C
274	WJ212	1	351	A/A	C/C
275	WJ213	1	351	A/A	C/C
276	WJ215	1	351	A/A	C/C
277	WJ216	1	351	A/A	C/C
278	WJ219	1	351	A/A	C/C
279	WJ220	1	351	A/A	C/C
280	WJ221	1	351	A/A	C/C
281	WJ223	1	351	A/A	C/C
282	WJ225	1	350	A/A	C/C
283	WJ227	1	350	A/A	C/C
284	WJ229	1	350	A/A	C/C
285	WJ240	1	350	A/A	C/C
286	WJ246	1	350	A/A	C/C
287	WJ25	1	350	A/A	C/C
288	WJ251	1	350	A/A	C/C
289	WJ252	1	350	A/A	C/C
290	WJ253	1	350	A/A	C/C
291	WJ257	1	350	A/A	C/C
292	WJ270	1	350	A/A	C/C
293	WJ271	1	350	A/A	C/C
294	WJ273	1	350	A/A	C/C
295	WJ277	1	350	A/A	C/C
296	WJ286	1	350	A/A	C/C
297	WJ29	1	350	A/A	C/C

298	WJ290	1	349	A/A	C/C
299	WJ299	1	349	A/A	C/C
300	WJ30	1	349	A/A	C/C
301	WJ304	1	349	A/A	C/C
302	WJ305	1	349	A/A	C/C
303	WJ31	1	349	A/A	C/C
304	WJ312	1	349	A/A	C/C
305	WJ32	1	349	A/A	C/C
306	WJ323	1	349	A/A	C/C
307	WJ325	1	349	A/A	C/C
308	WJ329	1	349	A/A	C/C
309	WJ33	1	349	A/A	C/C
310	WJ330	1	349	A/A	C/C
311	WJ332	1	349	A/A	C/C
312	WJ339	1	348	A/A	C/C
313	WJ343	1	348	A/A	C/C
314	WJ345	1	348	A/A	C/C
315	WJ349	1	348	A/A	C/C
316	WJ35	1	348	A/A	C/C
317	WJ359	1	348	A/A	C/C
318	WJ4	1	348	A/A	C/C
319	WJ41	1	348	A/A	C/C
320	WJ42	1	348	A/A	C/C
321	WJ47	1	348	A/A	C/C
322	WJ49	1	348	A/A	C/C
323	WJ52	1	348	A/A	C/C
324	WJ55	1	348	A/A	C/C
325	WJ56	1	348	A/A	C/C
326	WJ57	1	348	A/A	C/C
327	WJ58	1	348	A/A	C/C
328	WJ60	1	347	A/A	C/C
329	WJ61	1	347	A/A	C/C
330	WJ66	1	347	A/A	C/C
331	WJ69	1	347	A/A	C/C
332	WJ7	1	347	A/A	C/C
333	WJ71	1	347	A/A	C/C
334	WJ74	1	347	A/A	C/C
335	WJ78	1	347	A/A	C/C
336	WJ81	1	347	A/A	C/C
337	WJ82	1	347	A/A	C/C
338	WJ83	1	347	A/A	C/C
339	WJ85	1	347	A/A	C/C
340	WJ87	1	347	A/A	C/C

341	WJ88	1	347	A/A	C/C
342	WJ9	1	347	A/A	C/C
343	WJ91	1	347	A/A	C/C
344	WJ92	1	347	A/A	C/C
345	WJ93	1	347	A/A	C/C
346	WJ94	1	347	A/A	C/C
347	WJ95	1	347	A/A	C/C
348	WJ96	1	347	A/A	C/C
349	WJ98	1	347	A/A	C/C
350	Y10	1	347	A/A	C/C
351	Y103	1	347	A/A	C/C
352	Y11	1	347	A/A	C/C
353	Y118	1	347	A/A	C/C
354	Y119	1	347	A/A	C/C
355	Y2	1	347	A/A	C/C
356	Y20	1	347	A/A	C/C
357	Y24	1	347	A/A	C/C
358	Y26	1	347	A/A	C/C
359	Y3	1	347	A/A	C/C
360	Y30	1	347	A/A	C/C
361	Y31	1	347	A/A	C/C
362	Y32	1	347	A/A	C/C
363	Y34	1	347	A/A	C/C
364	Y36	1	347	A/A	C/C
365	Y37	1	347	A/A	C/C
366	Y38	1	347	A/A	C/C
367	Y40	1	347	A/A	C/C
368	Y43	1	347	A/A	C/C
369	Y46	1	347	A/A	C/C
370	Y50	1	346	A/A	C/C
371	Y54	1	346	A/A	C/C
372	Y56	1	346	A/A	C/C
373	Y61	1	346	A/A	C/C
374	Y67	1	346	A/A	C/C
375	Y70	1	346	A/A	C/C
376	Y74	1	346	A/A	C/C
377	Y76	1	346	A/A	C/C
378	Y78	1	346	A/A	C/C
379	Y8	1	346	A/A	C/C
380	D10	1	346	A/G	C/C
381	D11	1	346	A/G	C/C
382	D207	1	345	A/G	C/C
383	D22	1	345	A/G	C/C

384	D25	1	345	A/G	C/C
385	D301	1	345	A/G	C/C
386	D331	1	345	A/G	C/C
387	D351	1	345	A/G	C/C
388	D358	1	345	A/G	C/C
389	D37	1	345	A/G	C/C
390	D374	1	344	A/G	C/C
391	D375	1	344	A/G	C/C
392	D376	1	344	A/G	C/C
393	D378	1	344	A/G	C/C
394	D385	1	344	A/G	C/C
395	D388	1	344	A/G	C/C
396	D41	1	344	A/G	C/C
397	D412	1	344	A/G	C/C
398	D414	1	344	A/G	C/C
399	D420	1	344	A/G	C/C
400	D421	1	344	A/G	C/C
401	D427	2	343	A/G	C/C
402	D43	2	343	A/G	C/C
403	D438	2	343	A/G	C/C
404	D44	2	343	A/G	C/C
405	D50	2	343	A/G	C/C
406	D55	2	343	A/G	C/C
407	D56	2	343	A/G	C/C
408	D8	2	343	A/G	C/C
409	LD104	2	343	A/G	C/C
410	LD106	2	343	A/G	C/C
411	LD110	2	343	A/G	C/C
412	LD114	2	343	A/G	C/C
413	LD115	2	343	A/G	C/C
414	LD116	2	343	A/G	C/C
415	LD127	2	343	A/G	C/C
416	LD129	2	343	A/G	C/C
417	LD130	2	343	A/G	C/C
418	LD14	2	343	A/G	C/C
419	LD142	2	343	A/G	C/C
420	LD146	2	342	A/G	C/C
421	LD148	2	342	A/G	C/C
422	LD150	2	342	A/G	C/C
423	LD152	2	342	A/G	C/C
424	LD154	2	342	A/G	C/C
425	LD155	2	342	A/G	C/C
426	LD161	2	342	A/G	C/C

427	LD162	2	342	A/G	C/C
428	LD17	2	342	A/G	C/C
429	LD18	2	342	A/G	C/C
430	LD19	2	342	A/G	C/C
431	LD24	2	342	A/G	C/C
432	LD3	2	342	A/G	C/C
433	LD35	2	341	A/G	C/C
434	LD37	2	341	A/G	C/C
435	LD38	2	341	A/G	C/C
436	LD42	2	341	A/G	C/C
437	LD5	2	341	A/G	C/C
438	LD77	2	341	A/G	C/C
439	S111	2	341	A/G	C/C
440	S127	2	341	A/G	C/C
441	S136	2	341	A/G	C/C
442	S3	2	341	A/G	C/C
443	WJ166	2	341	A/G	C/C
444	WJ2	2	341	A/G	C/C
445	WJ209	2	341	A/G	C/C
446	Y100	2	340	A/G	C/C
447	Y102	2	340	A/G	C/C
448	Y12	2	340	A/G	C/C
449	Y120	2	340	A/G	C/C
450	Y126	2	340	A/G	C/C
451	Y22	2	340	A/G	C/C
452	Y35	2	340	A/G	C/C
453	Y5	2	340	A/G	C/C
454	Y52	2	340	A/G	C/C
455	Y58	2	339	A/G	C/C
456	Y6	2	339	A/G	C/C
457	Y66	2	339	A/G	C/C
458	Y68	2	339	A/G	C/C
459	Y7	2	339	A/G	C/C
460	Y72	2	339	A/G	C/C
461	Y9	2	339	A/G	C/C
462	D100	1	339	G/G	C/C
463	D108	1	339	G/G	C/C
464	D113	1	339	G/G	C/C
465	D124	1	338	G/G	C/C
466	D205	1	338	G/G	C/C
467	D217	1	338	G/G	C/C
468	D223	1	338	G/G	C/C
469	D227	1	338	G/G	C/C

470	D239	1	338	G/G	C/C
471	D253	1	338	G/G	C/C
472	D259	1	338	G/G	C/C
473	D265	2	338	G/G	C/C
474	D281	2	338	G/G	C/C
475	D329	2	338	G/G	C/C
476	D340	2	338	G/G	C/C
477	D63	2	338	G/G	C/C
478	D68	2	338	G/G	C/C
479	LD100	2	338	G/G	C/C
480	LD13	2	337	G/G	C/C
481	LD15	2	337	G/G	C/C
482	D105	1	337	A/A	T/C
483	D109	1	337	A/A	T/C
484	D111	1	337	A/A	T/C
485	D112	1	337	A/A	T/C
486	D118	1	337	A/A	T/C
487	D12	1	337	A/A	T/C
488	D121	1	337	A/A	T/C
489	D123	1	337	A/A	T/C
490	D125	1	337	A/A	T/C
491	D127	1	337	A/A	T/C
492	D132	1	337	A/A	T/C
493	D133	1	337	A/A	T/C
494	D134	1	337	A/A	T/C
495	D136	1	337	A/A	T/C
496	D137	1	337	A/A	T/C
497	D144	1	337	A/A	T/C
498	D145	1	337	A/A	T/C
499	D147	1	337	A/A	T/C
500	D15	1	337	A/A	T/C
501	D152	1	337	A/A	T/C
502	D156	1	337	A/A	T/C
503	D159	1	337	A/A	T/C
504	D16	1	337	A/A	T/C
505	D161	1	336	A/A	T/C
506	D162	1	336	A/A	T/C
507	D164	1	336	A/A	T/C
508	D167	1	336	A/A	T/C
509	D169	1	336	A/A	T/C
510	D177	1	336	A/A	T/C
511	D179	1	336	A/A	T/C
512	D180	1	336	A/A	T/C

513	D183	1	336	A/A	T/C
514	D184	1	336	A/A	T/C
515	D188	1	336	A/A	T/C
516	D196	1	336	A/A	T/C
517	D198	1	336	A/A	T/C
518	D199	1	336	A/A	T/C
519	D204	1	336	A/A	T/C
520	D206	1	336	A/A	T/C
521	D209	1	336	A/A	T/C
522	D21	1	336	A/A	T/C
523	D210	1	336	A/A	T/C
524	D213	1	336	A/A	T/C
525	D219	1	336	A/A	T/C
526	D221	1	336	A/A	T/C
527	D224	1	335	A/A	T/C
528	D225	1	335	A/A	T/C
529	D228	1	335	A/A	T/C
530	D230	1	335	A/A	T/C
531	D234	1	335	A/A	T/C
532	D235	1	335	A/A	T/C
533	D237	1	335	A/A	T/C
534	D241	1	335	A/A	T/C
535	D245	1	335	A/A	T/C
536	D249	1	335	A/A	T/C
537	D251	1	335	A/A	T/C
538	D257	1	335	A/A	T/C
539	D260	1	335	A/A	T/C
540	D263	1	335	A/A	T/C
541	D266	1	335	A/A	T/C
542	D271	1	335	A/A	T/C
543	D276	1	335	A/A	T/C
544	D277	1	335	A/A	T/C
545	D278	1	335	A/A	T/C
546	D28	1	335	A/A	T/C
547	D282	1	335	A/A	T/C
548	D288	1	335	A/A	T/C
549	D29	1	335	A/A	T/C
550	D292	1	335	A/A	T/C
551	D297	1	335	A/A	T/C
552	D3	1	335	A/A	T/C
553	D30	1	335	A/A	T/C
554	D300	1	335	A/A	T/C
555	D304	1	335	A/A	T/C

556	D308	1	335	A/A	T/C
557	D311	1	335	A/A	T/C
558	D315	1	335	A/A	T/C
559	D319	1	335	A/A	T/C
560	D32	1	335	A/A	T/C
561	D320	1	335	A/A	T/C
562	D322	1	335	A/A	T/C
563	D334	1	334	A/A	T/C
564	D335	1	334	A/A	T/C
565	D338	1	334	A/A	T/C
566	D341	1	334	A/A	T/C
567	D344	1	334	A/A	T/C
568	D345	1	334	A/A	T/C
569	D349	1	334	A/A	T/C
570	D355	1	334	A/A	T/C
571	D357	1	334	A/A	T/C
572	D361	1	334	A/A	T/C
573	D363	1	334	A/A	T/C
574	D364	1	334	A/A	T/C
575	D365	1	334	A/A	T/C
576	D366	1	334	A/A	T/C
577	D367	1	334	A/A	T/C
578	D371	1	334	A/A	T/C
579	D373	1	334	A/A	T/C
580	D38	1	334	A/A	T/C
581	D387	1	334	A/A	T/C
582	D389	1	334	A/A	T/C
583	D390	1	334	A/A	T/C
584	D391	1	334	A/A	T/C
585	D392	1	334	A/A	T/C
586	D393	1	334	A/A	T/C
587	D394	1	334	A/A	T/C
588	D395	1	334	A/A	T/C
589	D396	1	334	A/A	T/C
590	D397	1	334	A/A	T/C
591	D400	1	334	A/A	T/C
592	D401	1	334	A/A	T/C
593	D403	1	334	A/A	T/C
594	D406	1	334	A/A	T/C
595	D408	1	333	A/A	T/C
596	D409	1	333	A/A	T/C
597	D419	1	333	A/A	T/C
598	D424	1	333	A/A	T/C

599	D428	1	333	A/A	T/C
600	D430	1	333	A/A	T/C
601	D431	1	333	A/A	T/C
602	D433	1	333	A/A	T/C
603	D435	1	333	A/A	T/C
604	D439	1	333	A/A	T/C
605	D48	1	333	A/A	T/C
606	D49	1	333	A/A	T/C
607	D5	1	333	A/A	T/C
608	D64	1	333	A/A	T/C
609	D66	1	333	A/A	T/C
610	D75	1	333	A/A	T/C
611	D78	1	333	A/A	T/C
612	D79	1	333	A/A	T/C
613	D80	1	333	A/A	T/C
614	D81	1	333	A/A	T/C
615	D85	1	333	A/A	T/C
616	D91	1	333	A/A	T/C
617	D96	1	332	A/A	T/C
618	D99	1	332	A/A	T/C
619	LD10	1	332	A/A	T/C
620	LD102	1	332	A/A	T/C
621	LD112	1	332	A/A	T/C
622	LD118	1	332	A/A	T/C
623	LD140	1	332	A/A	T/C
624	LD143	1	332	A/A	T/C
625	LD2	1	332	A/A	T/C
626	LD21	1	332	A/A	T/C
627	LD22	1	332	A/A	T/C
628	LD23	1	332	A/A	T/C
629	LD25	1	332	A/A	T/C
630	LD27	1	332	A/A	T/C
631	LD45	1	332	A/A	T/C
632	LD46	1	332	A/A	T/C
633	LD51	1	332	A/A	T/C
634	LD58	1	332	A/A	T/C
635	LD59	1	332	A/A	T/C
636	LD62	1	332	A/A	T/C
637	LD68	1	332	A/A	T/C
638	LD74	1	331	A/A	T/C
639	LD80	1	331	A/A	T/C
640	LD83	1	331	A/A	T/C
641	LD87	1	331	A/A	T/C

642	LD89	1	331	A/A	T/C
643	S1	1	331	A/A	T/C
644	S10	1	331	A/A	T/C
645	S100	1	331	A/A	T/C
646	S102	1	331	A/A	T/C
647	S103	1	331	A/A	T/C
648	S105	1	331	A/A	T/C
649	S113	1	331	A/A	T/C
650	S114	1	331	A/A	T/C
651	S118	1	331	A/A	T/C
652	S119	1	331	A/A	T/C
653	S120	1	331	A/A	T/C
654	S122	1	331	A/A	T/C
655	S123	1	331	A/A	T/C
656	S126	1	331	A/A	T/C
657	S130	1	331	A/A	T/C
658	S134	1	331	A/A	T/C
659	S137	1	331	A/A	T/C
660	S138	1	331	A/A	T/C
661	S14	1	331	A/A	T/C
662	S140	1	331	A/A	T/C
663	S16	1	331	A/A	T/C
664	S17	1	331	A/A	T/C
665	S18	1	331	A/A	T/C
666	S19	1	331	A/A	T/C
667	S23	1	331	A/A	T/C
668	S24	1	331	A/A	T/C
669	S34	1	331	A/A	T/C
670	S35	1	330	A/A	T/C
671	S36	1	330	A/A	T/C
672	S37	1	330	A/A	T/C
673	S39	1	330	A/A	T/C
674	S41	1	330	A/A	T/C
675	S46	1	330	A/A	T/C
676	S47	1	330	A/A	T/C
677	S48	1	330	A/A	T/C
678	S49	1	330	A/A	T/C
679	S5	1	330	A/A	T/C
680	S52	1	330	A/A	T/C
681	S55	1	330	A/A	T/C
682	S56	1	330	A/A	T/C
683	S57	1	330	A/A	T/C
684	S58	1	330	A/A	T/C

685	S59	1	330	A/A	T/C
686	S62	1	330	A/A	T/C
687	S63	1	330	A/A	T/C
688	S67	1	330	A/A	T/C
689	S69	1	330	A/A	T/C
690	S7	1	330	A/A	T/C
691	S70	1	330	A/A	T/C
692	S73	1	330	A/A	T/C
693	S75	1	330	A/A	T/C
694	S76	1	329	A/A	T/C
695	S78	1	329	A/A	T/C
696	S79	1	329	A/A	T/C
697	S8	1	329	A/A	T/C
698	S80	1	329	A/A	T/C
699	S82	1	329	A/A	T/C
700	S83	1	329	A/A	T/C
701	S86	1	329	A/A	T/C
702	S88	1	329	A/A	T/C
703	S90	1	329	A/A	T/C
704	S91	1	329	A/A	T/C
705	S97	1	329	A/A	T/C
706	WJ1	1	329	A/A	T/C
707	WJ101	1	329	A/A	T/C
708	WJ105	1	329	A/A	T/C
709	WJ106	1	329	A/A	T/C
710	WJ109	1	329	A/A	T/C
711	WJ11	1	329	A/A	T/C
712	WJ110	1	329	A/A	T/C
713	WJ112	1	329	A/A	T/C
714	WJ116	1	329	A/A	T/C
715	WJ118	1	329	A/A	T/C
716	WJ121	1	328	A/A	T/C
717	WJ124	1	328	A/A	T/C
718	WJ127	1	328	A/A	T/C
719	WJ131	1	328	A/A	T/C
720	WJ136	1	328	A/A	T/C
721	WJ137	1	328	A/A	T/C
722	WJ138	1	328	A/A	T/C
723	WJ139	1	328	A/A	T/C
724	WJ140	1	328	A/A	T/C
725	WJ142	1	328	A/A	T/C
726	WJ144	1	328	A/A	T/C
727	WJ147	1	328	A/A	T/C

728	WJ148	1	328	A/A	T/C
729	WJ150	1	328	A/A	T/C
730	WJ153	1	328	A/A	T/C
731	WJ154	1	328	A/A	T/C
732	WJ155	1	328	A/A	T/C
733	WJ156	1	328	A/A	T/C
734	WJ16	1	328	A/A	T/C
735	WJ160	1	328	A/A	T/C
736	WJ162	1	327	A/A	T/C
737	WJ165	1	327	A/A	T/C
738	WJ167	1	327	A/A	T/C
739	WJ171	1	327	A/A	T/C
740	WJ172	1	327	A/A	T/C
741	WJ174	1	327	A/A	T/C
742	WJ176	1	327	A/A	T/C
743	WJ178	1	327	A/A	T/C
744	WJ180	1	327	A/A	T/C
745	WJ181	1	327	A/A	T/C
746	WJ184	1	327	A/A	T/C
747	WJ185	1	327	A/A	T/C
748	WJ187	1	327	A/A	T/C
749	WJ19	1	327	A/A	T/C
750	WJ194	1	327	A/A	T/C
751	WJ196	1	327	A/A	T/C
752	WJ199	1	327	A/A	T/C
753	WJ200	1	327	A/A	T/C
754	WJ201	1	327	A/A	T/C
755	WJ202	1	327	A/A	T/C
756	WJ205	1	327	A/A	T/C
757	WJ206	1	327	A/A	T/C
758	WJ208	1	327	A/A	T/C
759	WJ21	1	327	A/A	T/C
760	WJ214	1	326	A/A	T/C
761	WJ22	1	326	A/A	T/C
762	WJ224	1	326	A/A	T/C
763	WJ226	1	326	A/A	T/C
764	WJ23	1	326	A/A	T/C
765	WJ231	1	326	A/A	T/C
766	WJ232	1	326	A/A	T/C
767	WJ233	1	326	A/A	T/C
768	WJ235	1	326	A/A	T/C
769	WJ236	1	326	A/A	T/C
770	WJ237	1	326	A/A	T/C

771	WJ238	1	326	A/A	T/C
772	WJ239	1	326	A/A	T/C
773	WJ24	1	326	A/A	T/C
774	WJ242	1	326	A/A	T/C
775	WJ243	1	326	A/A	T/C
776	WJ244	1	326	A/A	T/C
777	WJ245	1	326	A/A	T/C
778	WJ247	1	326	A/A	T/C
779	WJ249	1	325	A/A	T/C
780	WJ255	1	325	A/A	T/C
781	WJ256	1	325	A/A	T/C
782	WJ258	1	325	A/A	T/C
783	WJ260	1	325	A/A	T/C
784	WJ262	1	325	A/A	T/C
785	WJ263	1	325	A/A	T/C
786	WJ264	1	325	A/A	T/C
787	WJ267	1	325	A/A	T/C
788	WJ268	1	325	A/A	T/C
789	WJ27	1	325	A/A	T/C
790	WJ272	1	325	A/A	T/C
791	WJ274	1	325	A/A	T/C
792	WJ275	1	325	A/A	T/C
793	WJ278	1	325	A/A	T/C
794	WJ28	1	325	A/A	T/C
795	WJ280	1	325	A/A	T/C
796	WJ281	1	325	A/A	T/C
797	WJ283	1	325	A/A	T/C
798	WJ285	1	325	A/A	T/C
799	WJ287	1	325	A/A	T/C
800	WJ288	1	325	A/A	T/C
801	WJ289	1	325	A/A	T/C
802	WJ292	1	325	A/A	T/C
803	WJ294	1	324	A/A	T/C
804	WJ295	1	324	A/A	T/C
805	WJ296	1	324	A/A	T/C
806	WJ298	1	324	A/A	T/C
807	WJ3	1	324	A/A	T/C
808	WJ300	1	324	A/A	T/C
809	WJ301	1	324	A/A	T/C
810	WJ302	1	324	A/A	T/C
811	WJ303	1	324	A/A	T/C
812	WJ306	1	324	A/A	T/C
813	WJ307	1	324	A/A	T/C

814	WJ308	1	324	A/A	T/C
815	WJ310	1	324	A/A	T/C
816	WJ311	1	324	A/A	T/C
817	WJ313	1	324	A/A	T/C
818	WJ315	1	324	A/A	T/C
819	WJ316	1	324	A/A	T/C
820	WJ317	1	324	A/A	T/C
821	WJ318	1	324	A/A	T/C
822	WJ320	1	324	A/A	T/C
823	WJ321	1	324	A/A	T/C
824	WJ322	1	324	A/A	T/C
825	WJ324	1	324	A/A	T/C
826	WJ327	1	324	A/A	T/C
827	WJ328	1	323	A/A	T/C
828	WJ331	1	323	A/A	T/C
829	WJ333	1	323	A/A	T/C
830	WJ337	1	323	A/A	T/C
831	WJ338	1	323	A/A	T/C
832	WJ34	1	323	A/A	T/C
833	WJ340	1	323	A/A	T/C
834	WJ341	1	323	A/A	T/C
835	WJ342	1	323	A/A	T/C
836	WJ344	1	323	A/A	T/C
837	WJ346	1	323	A/A	T/C
838	WJ350	1	323	A/A	T/C
839	WJ353	1	323	A/A	T/C
840	WJ354	1	323	A/A	T/C
841	WJ356	1	323	A/A	T/C
842	WJ357	1	323	A/A	T/C
843	WJ358	1	323	A/A	T/C
844	WJ36	1	323	A/A	T/C
845	WJ362	1	323	A/A	T/C
846	WJ363	1	323	A/A	T/C
847	WJ364	1	323	A/A	T/C
848	WJ366	1	323	A/A	T/C
849	WJ37	1	323	A/A	T/C
850	WJ38	1	323	A/A	T/C
851	WJ39	1	323	A/A	T/C
852	WJ44	1	323	A/A	T/C
853	WJ45	1	323	A/A	T/C
854	WJ46	1	322	A/A	T/C
855	WJ48	1	322	A/A	T/C
856	WJ5	1	322	A/A	T/C

857	WJ53	1	322	A/A	T/C
858	WJ54	1	322	A/A	T/C
859	WJ59	1	322	A/A	T/C
860	WJ6	1	322	A/A	T/C
861	WJ62	1	322	A/A	T/C
862	WJ63	1	322	A/A	T/C
863	WJ64	1	322	A/A	T/C
864	WJ67	1	322	A/A	T/C
865	WJ68	1	322	A/A	T/C
866	WJ70	1	322	A/A	T/C
867	WJ72	1	322	A/A	T/C
868	WJ73	1	322	A/A	T/C
869	WJ75	1	322	A/A	T/C
870	WJ76	1	322	A/A	T/C
871	WJ77	1	322	A/A	T/C
872	WJ79	1	322	A/A	T/C
873	WJ8	1	321	A/A	T/C
874	WJ80	1	321	A/A	T/C
875	WJ84	1	321	A/A	T/C
876	WJ86	1	321	A/A	T/C
877	WJ89	1	321	A/A	T/C
878	WJ90	1	321	A/A	T/C
879	WJ97	1	321	A/A	T/C
880	WJ99	1	321	A/A	T/C
881	Y1	1	321	A/A	T/C
882	Y101	1	321	A/A	T/C
883	Y104	1	321	A/A	T/C
884	Y105	1	321	A/A	T/C
885	Y110	1	321	A/A	T/C
886	Y113	1	321	A/A	T/C
887	Y114	1	321	A/A	T/C
888	Y122	1	321	A/A	T/C
889	Y128	1	320	A/A	T/C
890	Y13	1	320	A/A	T/C
891	Y131	1	320	A/A	T/C
892	Y133	1	320	A/A	T/C
893	Y135	1	320	A/A	T/C
894	Y14	1	320	A/A	T/C
895	Y15	1	320	A/A	T/C
896	Y16	1	320	A/A	T/C
897	Y17	1	320	A/A	T/C
898	Y18	1	320	A/A	T/C
899	Y19	1	320	A/A	T/C

900	Y23	1	320	A/A	T/C
901	Y28	1	320	A/A	T/C
902	Y29	1	320	A/A	T/C
903	Y4	1	320	A/A	T/C
904	Y41	1	320	A/A	T/C
905	Y42	1	320	A/A	T/C
906	Y44	1	319	A/A	T/C
907	Y48	1	319	A/A	T/C
908	Y49	1	319	A/A	T/C
909	Y51	1	319	A/A	T/C
910	Y55	1	319	A/A	T/C
911	Y57	1	319	A/A	T/C
912	Y59	1	319	A/A	T/C
913	Y60	1	319	A/A	T/C
914	Y62	1	319	A/A	T/C
915	Y63	1	319	A/A	T/C
916	Y71	1	319	A/A	T/C
917	Y75	1	319	A/A	T/C
918	Y81	1	319	A/A	T/C
919	Y82	1	319	A/A	T/C
920	Y85	1	318	A/A	T/C
921	Y86	1	318	A/A	T/C
922	Y88	1	318	A/A	T/C
923	Y89	1	318	A/A	T/C
924	D1	2	318	A/G	T/C
925	D14	2	318	A/G	T/C
926	D18	2	318	A/G	T/C
927	D197	2	318	A/G	T/C
928	D348	2	318	A/G	T/C
929	D350	2	318	A/G	T/C
930	D382	2	318	A/G	T/C
931	D410	2	318	A/G	T/C
932	D422	2	317	A/G	T/C
933	D423	2	317	A/G	T/C
934	D429	2	317	A/G	T/C
935	D51	2	317	A/G	T/C
936	D53	2	317	A/G	T/C
937	D54	2	317	A/G	T/C
938	D57	2	317	A/G	T/C
939	LD107	2	317	A/G	T/C
940	LD11	2	317	A/G	T/C
941	LD117	2	317	A/G	T/C
942	LD119	2	317	A/G	T/C

943	LD12	2	317	A/G	T/C
944	LD122	2	317	A/G	T/C
945	LD123	2	317	A/G	T/C
946	LD125	2	317	A/G	T/C
947	LD126	2	317	A/G	T/C
948	LD128	2	316	A/G	T/C
949	LD131	2	316	A/G	T/C
950	LD134	2	316	A/G	T/C
951	LD137	2	316	A/G	T/C
952	LD139	2	316	A/G	T/C
953	LD141	2	316	A/G	T/C
954	LD144	2	316	A/G	T/C
955	LD145	2	316	A/G	T/C
956	LD147	2	316	A/G	T/C
957	LD151	2	316	A/G	T/C
958	LD153	2	316	A/G	T/C
959	LD156	2	316	A/G	T/C
960	LD157	2	315	A/G	T/C
961	LD16	2	315	A/G	T/C
962	LD160	2	315	A/G	T/C
963	LD166	2	315	A/G	T/C
964	LD33	2	315	A/G	T/C
965	LD4	2	315	A/G	T/C
966	LD49	2	315	A/G	T/C
967	LD50	2	315	A/G	T/C
968	LD53	2	315	A/G	T/C
969	LD57	2	315	A/G	T/C
970	LD6	2	314	A/G	T/C
971	LD60	2	314	A/G	T/C
972	LD65	2	314	A/G	T/C
973	LD66	2	314	A/G	T/C
974	LD67	2	314	A/G	T/C
975	LD70	2	314	A/G	T/C
976	LD71	2	314	A/G	T/C
977	LD76	2	314	A/G	T/C
978	LD78	2	314	A/G	T/C
979	LD82	2	314	A/G	T/C
980	LD88	2	314	A/G	T/C
981	LD90	2	314	A/G	T/C
982	LD93	2	314	A/G	T/C
983	LD94	2	314	A/G	T/C
984	LD95	2	314	A/G	T/C
985	S38	2	314	A/G	T/C

986	S43	2	314	A/G	T/C
987	WJ103	2	314	A/G	T/C
988	WJ123	2	313	A/G	T/C
989	WJ173	2	313	A/G	T/C
990	WJ336	2	313	A/G	T/C
991	WJ43	2	313	A/G	T/C
992	Y107	2	313	A/G	T/C
993	Y109	2	313	A/G	T/C
994	Y112	2	313	A/G	T/C
995	Y116	2	313	A/G	T/C
996	Y121	2	313	A/G	T/C
997	Y123	2	313	A/G	T/C
998	Y125	2	313	A/G	T/C
999	Y129	2	313	A/G	T/C
1000	Y130	2	312	A/G	T/C
1001	Y21	2	312	A/G	T/C
1002	Y39	2	312	A/G	T/C
1003	Y45	2	312	A/G	T/C
1004	Y79	2	312	A/G	T/C
1005	Y80	2	312	A/G	T/C
1006	Y84	2	312	A/G	T/C
1007	Y87	2	312	A/G	T/C
1008	Y90	2	312	A/G	T/C
1009	Y91	2	312	A/G	T/C
1010	Y92	2	312	A/G	T/C
1011	Y95	2	312	A/G	T/C
1012	Y96	2	312	A/G	T/C
1013	Y98	2	311	A/G	T/C
1014	Y99	2	311	A/G	T/C
1015	D106	2	311	G/G	T/C
1016	D110	2	311	G/G	T/C
1017	D114	2	311	G/G	T/C
1018	D116	2	311	G/G	T/C
1019	D117	2	311	G/G	T/C
1020	D126	2	311	G/G	T/C
1021	D130	2	311	G/G	T/C
1022	D135	2	311	G/G	T/C
1023	D146	2	311	G/G	T/C
1024	D168	2	311	G/G	T/C
1025	D174	2	311	G/G	T/C
1026	D175	2	311	G/G	T/C
1027	D193	2	310	G/G	T/C
1028	D218	2	310	G/G	T/C

1029	D226	2	310	G/G	T/C
1030	D238	2	310	G/G	T/C
1031	D240	2	310	G/G	T/C
1032	D244	2	305	G/G	T/C
1033	D261	2	309	G/G	T/C
1034	D275	2	298	G/G	T/C
1035	D287	2	288	G/G	T/C
1036	D291	2	286	G/G	T/C
1037	D318	2	285	G/G	T/C
1038	D339	2	303	G/G	T/C
1039	D62	2	302	G/G	T/C
1040	D69	2	301	G/G	T/C
1041	D70	2	301	G/G	T/C
1042	D72	2	301	G/G	T/C
1043	LD103	2	301	G/G	T/C
1044	LD159	2	300	G/G	T/C
1045	LD44	2	299	G/G	T/C
1046	LD47	2	299	G/G	T/C
1047	LD52	2	298	G/G	T/C
1048	LD63	2	288	G/G	T/C
1049	LD8	2	286	G/G	T/C
1050	LD86	2	285	G/G	T/C
1051	Y108	2	309	G/G	T/C
1052	Y47	2	309	G/G	T/C
1053	S54	1	307	A/A	T/T
1054	S6	1	307	A/A	T/T
1055	S66	1	307	A/A	T/T
1056	S74	1	307	A/A	T/T
1057	S77	1	307	A/A	T/T
1058	S84	1	307	A/A	T/T
1059	S92	1	307	A/A	T/T
1060	S94	1	307	A/A	T/T
1061	WJ348	1	306	A/A	T/T
1062	WJ351	1	306	A/A	T/T
1063	WJ352	1	306	A/A	T/T
1064	WJ355	1	306	A/A	T/T
1065	WJ360	1	306	A/A	T/T
1066	WJ361	1	306	A/A	T/T
1067	WJ365	1	306	A/A	T/T
1068	WJ367	1	306	A/A	T/T
1069	WJ40	1	306	A/A	T/T
1070	WJ50	1	305	A/A	T/T
1071	WJ51	1	305	A/A	T/T

1072	Y106	1	305	A/A	T/T
1073	Y111	1	305	A/A	T/T
1074	Y115	1	305	A/A	T/T
1075	Y124	1	310	A/A	T/T
1076	Y127	1	310	A/A	T/T
1077	Y134	1	310	A/A	T/T
1078	Y25	1	310	A/A	T/T
1079	Y27	1	310	A/A	T/T
1080	Y33	1	310	A/A	T/T
1081	Y53	1	310	A/A	T/T
1082	Y64	1	310	A/A	T/T
1083	Y65	1	310	A/A	T/T
1084	Y69	1	310	A/A	T/T
1085	Y77	2	310	A/A	T/T
1086	Y83	2	310	A/A	T/T
1087	D142	2	310	A/G	T/T
1088	D307	2	310	A/G	T/T
1089	D4	2	309	A/G	T/T
1090	LD113	2	309	A/G	T/T
1091	LD124	2	309	A/G	T/T
1092	LD149	2	309	A/G	T/T
1093	LD163	2	309	A/G	T/T
1094	LD34	2	310	A/G	T/T
1095	LD41	2	309	A/G	T/T
1096	LD56	2	305	A/G	T/T
1097	LD7	2	304	A/G	T/T
1098	LD84	2	304	A/G	T/T
1099	LD85	2	304	A/G	T/T
1100	LD9	2	304	A/G	T/T
1101	LD97	2	304	A/G	T/T
1102	WJ319	2	304	A/G	T/T
1103	Y117	2	304	A/G	T/T
1104	Y132	2	304	A/G	T/T
1105	Y73	2	304	A/G	T/T
1106	Y93	2	304	A/G	T/T
1107	Y94	2	304	A/G	T/T
1108	Y97	2	284	A/G	T/T
1109	D220	2	282	G/G	T/T
1110	D246	2	265	G/G	T/T
1111	LD133	2	262	G/G	T/T
1112	G2	2	309	G/G	T/T
1113	G20	2	309	A/G	T/T
1114	G32	2	309	A/G	T/T

1115	G35	2	309	A/G	T/T
1116	LG7	3	309	A/G	T/T
1117	LG28	2	309	A/G	T/T
1118	LG43	2	309	A/G	T/T
1119	LG57	3	309	G/G	T/T
1120	LG60	2	309	A/G	T/T
1121	LG70	2	308	A/G	T/T
1122	LG81	2	308	G/G	T/T
1123	LG82	2	308	G/G	T/T
1124	LG83	2	308	A/G	T/T
1125	LG84	2	308	G/G	T/T
1126	LG86	2	308	A/G	T/T
1127	LG108	2	308	A/G	T/T
1128	LG127	2	308	G/G	T/T
1129	LG128	2	308	G/G	T/T
1130	LG141	2	307	A/G	T/T

Table S5. Statistics of ovarian and follicular phenotypes

Group	BW/kg	OW/kg	OW/BW	FN	DNF	DFD/mm	VDF/mm^3
LF1	44.2	1.8	0.04	4	1	8.76	351.97
		0.9	0.02	5			
LF2	43.45	1.8	0.04	11	1	8.55	327.26
		0.5	0.01	8			
LF3	40.85	1.6	0.04	8	1	8.5	321.56
		0.7	0.02	6			
HF1	53.2	1.2	0.02	5	2	4.61	51.30
		1.2	0.02	6		4.58	50.30
HF2	53.4	1.3	0.02	8	2	6.07	117.10
		1.1	0.02	3		6.53	145.79
HF3	51.15	1.8	0.04	5	2	5.96	110.85
		1.2	0.02	4		6.27	129.06

Notes: BW: body weight; OW: ovary weight; FN: follicle number; DNF: dominant follicle number; DFD: dominant follicle diameter; VDF: volume of dominant follicle.

Table S6. Information of genes expression for RNA sequencing (RNA-seq) on ovarian samples

Gene_id	Gene symbol	log2fc	pvalue	qvalue
121816622	*LOC121816622*	10.07	6.50E-14	7.28E-10
443217	*ITGAM*	3.03	7.09E-14	7.28E-10
101109959	*GPNMB*	3.72	1.70E-10	1.17E-06
114110979	*LOC114110979*	23.39	2.16E-09	1.11E-05
443365	*SLC11A1*	4.36	3.78E-09	1.56E-05
114116824	*LOC114116824*	-2.92	1.01E-08	3.47E-05
114118103	*LOC114118103*	-22.28	1.19E-08	3.50E-05
101123199	*CSF2RB*	2.22	1.64E-08	3.76E-05
101116587	*ALOX15*	-3.71	1.64E-08	3.76E-05
443038	*CAPN3*	3.71	3.09E-08	6.35E-05
101106094	*NCF2*	4.04	4.29E-08	7.93E-05
114109690	*LOC114109690*	-2.37	4.63E-08	7.93E-05
114115302	*LOC114115302*	20.67	1.24E-07	1.83E-04
101106078	*MARCHF1*	3.15	1.17E-07	1.83E-04
105603393	*LOC105603393*	20.48	1.61E-07	2.12E-04
101102301	*NAV3*	2.13	1.65E-07	2.12E-04
101123610	*NYAP2*	6.62	3.21E-07	3.65E-04
101113074	*PLCB2*	2.75	3.55E-07	3.65E-04
443122	*STAR*	2.33	3.11E-07	3.65E-04
101113369	*LOC101113369*	-1.92	3.55E-07	3.65E-04
105602100	*LOC105602100*	3.20	4.41E-07	4.32E-04
101123369	*SCIN*	3.15	5.94E-07	5.55E-04
101109494	*SLC37A2*	2.44	1.12E-06	9.99E-04
114111662	*CDR1*	2.17	1.19E-06	1.02E-03
101103023	*LOC101103023*	4.90	1.25E-06	1.03E-03
101109106	*SPAG7*	2.14	1.69E-06	1.34E-03
101120058	*ADAM28*	3.98	1.96E-06	1.40E-03
101123578	*LOC101123578*	2.55	1.93E-06	1.40E-03
101105239	*HOXA10*	-4.30	1.98E-06	1.40E-03
121817402	*LOC121817402*	-1.87	2.13E-06	1.46E-03
101112675	*RAI14*	1.89	2.71E-06	1.80E-03
101105303	*SYK*	2.89	2.88E-06	1.85E-03
114111248	*LOC114111248*	6.92	3.16E-06	1.97E-03
101105005	*VNN1*	3.88	4.02E-06	2.43E-03
114108725	*LOC114108725*	-2.75	4.74E-06	2.79E-03
101102344	*LOC101102344*	-1.79	5.17E-06	2.95E-03
105611415	*RTN4RL2*	4.06	8.58E-06	4.64E-03
101113926	*CRB2*	-3.70	8.56E-06	4.64E-03
101102212	*ECEL1*	6.45	9.29E-06	4.90E-03
101104513	*NPL*	3.07	1.06E-05	5.45E-03

101121746	*MSR1*	2.36	1.18E-05	5.77E-03
101120702	*LOC101120702*	-2.32	1.15E-05	5.77E-03
101120775	*FOLR3*	3.63	1.24E-05	5.94E-03
101118952	*GNA15*	2.23	1.28E-05	5.99E-03
101103753	*ADM*	2.33	1.59E-05	7.25E-03
105601865	*LOC105601865*	1.87	1.77E-05	7.92E-03
114110832	*LOC114110832*	2.48	2.40E-05	1.03E-02
101111582	*SCN5A*	1.90	2.39E-05	1.03E-02
101107815	*CNOT3*	1.78	2.54E-05	1.05E-02
101106582	*PLXNC1*	1.53	2.55E-05	1.05E-02
780489	*NCF1*	3.12	2.71E-05	1.09E-02
101115330	*FERMT3*	2.13	2.77E-05	1.10E-02
101115456	*ECSCR*	2.07	2.89E-05	1.12E-02
121819177	*LOC121819177*	3.99	3.15E-05	1.20E-02
101113658	*IHH*	-3.65	3.76E-05	1.40E-02
101122548	*GALNT18*	1.20	3.85E-05	1.41E-02
101120200	*LOC101120200*	-1.69	4.50E-05	1.62E-02
101113171	*CPT1C*	1.47	5.14E-05	1.82E-02
100302312	*ARL4C*	2.76	6.05E-05	2.11E-02
101114507	*BIN2*	2.45	6.71E-05	2.30E-02
101121536	*BFSP1*	3.88	7.01E-05	2.32E-02
121817401	*LOC121817401*	-2.46	6.93E-05	2.32E-02
101106006	*TGFBI*	1.49	7.46E-05	2.43E-02
101108643	*PTPN6*	2.20	8.10E-05	2.60E-02
101115503	*MLXIPL*	2.37	9.01E-05	2.85E-02
101107354	*VAV1*	1.81	1.16E-04	3.45E-02
101109742	*CLK3*	1.80	1.16E-04	3.45E-02
114109686	*LOC114109686*	-2.24	1.13E-04	3.45E-02
114110569	*IL32*	-2.34	1.15E-04	3.45E-02
101106955	*RGL1*	1.41	1.19E-04	3.51E-02
101103186	*SMTN*	1.30	1.24E-04	3.58E-02
101121193	*TMEM150B*	2.65	1.29E-04	3.65E-02
101120874	*GSTP1*	1.62	1.29E-04	3.65E-02
101105943	*GRIN2D*	1.68	1.52E-04	4.22E-02
101114036	*TK1*	-2.40	1.62E-04	4.45E-02
101103096	*LOC101103096*	1.29	1.66E-04	4.48E-02
URS0000AB010D	*URS0000AB010D*	-1.97	1.69E-04	4.51E-02
101115324	*CDCA8*	-2.50	1.77E-04	4.59E-02
101117946	*KRT19*	-3.50	1.75E-04	4.59E-02
101119464	*ETV6*	1.25	1.79E-04	4.61E-02
114118069	*LOC114118069*	2.13	1.86E-04	4.62E-02
101104011	*DOCK2*	1.81	1.86E-04	4.62E-02
101103521	*PRDX5*	-1.31	1.84E-04	4.62E-02

101105327	*DENND1C*	1.78	1.96E-04	4.75E-02
101119747	*CDH1*	-2.49	1.96E-04	4.75E-02
101111044	*MYBPC2*	2.07	2.07E-04	4.95E-02

Table S7. Statistics on the types of alternative splicing (AS) events per sample.

Sample	A3SS	A5SS	MXE	RI	SE	Total
HO1	5705	6428	1789	1502	19414	34838
HO2	5667	6375	1587	1499	17317	32445
HO3	5706	6416	1950	1504	21177	36753
LO1	5638	6340	1414	1500	15344	30236
LO2	5698	6418	1771	1505	19359	34751
LO3	5703	6415	1761	1506	19300	34685

Note: A3SS: alternative 3′ splice site; A5SS: alternative 5′ splice site; MXE: mutually exclusive exons; SE: skipped exon; RI: retained intron.

Table S8. Statistics on the differences between the two groups alternative splicing (AS) events

Type	Gene	1ES	1EE	2ES	2EE	upES	upEE	downES	downEE	IJC(LO)	SJC(LO)	IJC(HO)	SJC(HO)	P-value	FDR
MXE	*BMPR1B*	30081491	30081651	30195835	30195929	30069641	30069744	30254161	30254206	36,45,12	26,125,96	0,8,3	105,151,164	3.17E-09	4.02E-07
MXE	*BMPR1B*	30050588	30050781	30057212	30057351	30049283	30049581	30058658	30058755	12,50,41	25,79,52	17,15,0	48,75,72	5.92E-06	1.93E-04
MXE	*BMPR1B*	30195835	30195929	30254161	30254263	30081491	30081651	30482440	30482585	11,17,2	15,30,26	2,18,25	7,4,0	2.13E-03	1.90E-02
SE	*BMPR1B*	30037772	30037948			30033900	30034031	30049283	30,049,581	19,252,167	0,0,4	136,238,177	0,0,0	4.76E-01	1.00E+00
SE	*BMPR1B*	30195835	30195929			30081491	30081651	30254161	30254206	51,77,36	5,3,2	7,12,3	0,4,0	7.13E-01	1.00E+00
SE	*BMPR1B*	30050588	30050781			30049283	30049581	30057212	30057351	27,89,53	0,0,1	55,79,82	0,0,0	1.00E+00	1.00E+00
SE	*BMPR1B*	30057212	30057351			30050588	30050781	30058658	30058755	14,60,41	0,0,0	26,19,10	2,0,0	1.00E+00	1.00E+00
SE	*BMPR1B*	30081491	30081651			30069641	30069744	30195835	30,195,929	36,156,118	0,2,0	112,151,164	0,0,0	1.00E+00	1.00E+00
SE	*BMPR1B*	30254161	30254263			30195835	30195929	30482440	30482585	42,62,12	0,0,2	2,22,28	0,0,0	1.00E+00	1.00E+00

Note: 1ES: 1stExonStart; 1EE: 1stExonEnd; 2ES:2ndExonStart; 2EE: 2ndExonEnd; upES: upstreamES; upEE: upstreamEE; downES: downstreamES; downEE: downstreamEE; SJC: skipping junction counts; IJC: inclusion junction counts.

Table S9. Statistical quality control filtered cell count.

Sample Name	Estimated Number of Cells	Fraction Reads in Cells	Mean Reads per Cell	Median Genes per Cell	Total Genes Detected	Median UMI Counts per Cell	Filtered Min Count Cells	Filtered Doublet Cells	Filtered mt cells	Final Cell Num
HO1	26638	0.816	33016	1832	20512	3336	26638	21908	18618	18618
HO2	22327	0.737	38716	1696	20231	2755	22327	18384	15624	15624
HO3	25575	0.79	30881	2077	20564	3706	25575	20471	17395	17395
LO1	26601	0.59	28568	1908	20256	3061	26601	23499	19969	19969
LO2	14244	0.583	62937	2182	19806	4669	14244	12639	10737	10737
LO3	37944	0.673	23074	1404	20591	2090	37944	34368	29205	29205

Table S10. A list of ovarian marker genes in the references

Cellname	Marker gene	References
Epithelial cells (EP)	*ALDH1A2*	Pei et al. 2023
Epithelial cells (EP)	*ANXA2*	Pei et al. 2023
Epithelial cells (EP)	*BNC1*	Pei et al. 2023
Epithelial cells (EP)	*C3*	Pei et al. 2023
Epithelial cells (EP)	*CCN3*	Pei et al. 2023
Epithelial cells (EP)	*CLDN1*	Pei et al. 2023
Epithelial cells (EP)	*COL6A5*	Pei et al. 2023
Epithelial cells (EP)	*CST3*	Pei et al. 2023
Epithelial cells (EP)	*DSP*	Pei et al. 2023
Epithelial cells (EP)	*FBLN2*	Pei et al. 2023
Epithelial cells (EP)	*GAS6*	Pei et al. 2023
Epithelial cells (EP)	*GPM6A*	Pei et al. 2023
Epithelial cells (EP)	*LGALS3*	Pei et al. 2023
Epithelial cells (EP)	*LY6E*	Pei et al. 2023
Epithelial cells (EP)	*PKM*	Pei et al. 2023
Epithelial cells (EP)	*RSPO1*	Pei et al. 2023
Epithelial cells (EP)	*S100A16*	Pei et al. 2023
Epithelial cells (EP)	*SERPING1*	Pei et al. 2023
Epithelial cells (EP)	*TF*	Pei et al. 2023
Epithelial cells (EP)	*UPK3B*	Pei et al. 2023
Epithelial cells (EP)	*WT1*	Pei et al. 2023
Epithelial cells (EP)	*KRT19*	Sheng et al. 2022
Granulosa cells (GC)	*APOA1*	Pei et al. 2023
Granulosa cells (GC)	*APOD*	Pei et al. 2023
Granulosa cells (GC)	*APOE*	Pei et al. 2023
Granulosa cells (GC)	*ARFGAP3*	Pei et al. 2023
Granulosa cells (GC)	*COL4A1*	Pei et al. 2023
Granulosa cells (GC)	*COL4A2*	Pei et al. 2023
Granulosa cells (GC)	*CXCL14*	Pei et al. 2023
Granulosa cells (GC)	*CYB5R3*	Pei et al. 2023
Granulosa cells (GC)	*CYP11A1*	Pei et al. 2023
Granulosa cells (GC)	*DCTPP1*	Pei et al. 2023
Granulosa cells (GC)	*FDXR*	Pei et al. 2023
Granulosa cells (GC)	*GSTM3*	Pei et al. 2023
Granulosa cells (GC)	*IGFBP4*	Pei et al. 2023
Granulosa cells (GC)	*INHA*	Pei et al. 2023
Granulosa cells (GC)	*LRPAP1*	Pei et al. 2023
Granulosa cells (GC)	*P4HB*	Pei et al. 2023
Granulosa cells (GC)	*PDE5A*	Pei et al. 2023
Granulosa cells (GC)	*PRKAR2B*	Pei et al. 2023

Granulosa cells (GC)	*PRR5*	Pei et al. 2023
Granulosa cells (GC)	*PXDN*	Pei et al. 2023
Granulosa cells (GC)	*SMOC2*	Pei et al. 2023
Granulosa cells (GC)	*TIMP2*	Pei et al. 2023
Granulosa cells (GC)	*TKT*	Pei et al. 2023
Granulosa cells (GC)	*FOXL2*	Wagner et al.2020
Granulosa cells (GC)	*AMH*	Wagner et al.2020
Granulosa cells (GC)	*BEX1*	Wagner et al.2020
Granulosa cells (GC)	*FST*	Wagner et al.2020
Granulosa cells (GC)	*NR5A2*	Wang et al.2020
Granulosa cells (GC)	*AMH*	Fan et al.2019
Granulosa cells (GC)	*HSD17B1*	Fan et al.2019
Granulosa cells (GC)	*SERPINE2*	Fan et al.2019
Granulosa cells (GC)	*GSTA1*	Fan et al.2019
Granulosa cells (GC)	*WT1*	Fan et al.2019
Granulosa cells (GC)	*EGR4*	Fan et al.2019
Granulosa cells (GC)	*VCAN*	Fan et al.2019
Granulosa cells (GC)	*FST*	Fan et al.2019
Granulosa cells (GC)	*IGFBP2*	Fan et al.2019
Granulosa cells (GC)	*HTRA1*	Fan et al.2019
Granulosa cells (GC)	*INHBB*	Fan et al.2019
Granulosa cells (GC)	*IHH*	Fan et al.2019
Granulosa cells (GC)	*KRT18*	Fan et al.2019
Granulosa cells (GC)	*CITED2*	Fan et al.2019
Granulosa cells (GC)	*LIPH*	Fan et al.2019
Granulosa cells (GC)	*AKIRIN1*	Fan et al.2019
Granulosa cells (GC)	*TNNI3*	Fan et al.2019
Granulosa cells (GC)	*MAGED2*	Fan et al.2019
Granulosa cells (GC)	*SPINT2*	Fan et al.2019
Granulosa cells (GC)	*PLA2G16*	Fan et al.2019
Granulosa cells (GC)	*BEX1*	Fan et al.2019
Granulosa cells (GC)	*DSP*	Fan et al.2019
Granulosa cells (GC)	*TSPAN6*	Fan et al.2019
Granulosa cells (GC)	*LCMT1*	Fan et al.2019
Granulosa cells (GC)	*AMHR2*	Sheng et al. 2022
Granulosa cells (GC)	*AMHR2*	Wang et al.2021
Granulosa cells (GC)	*KITL*	Wang et al.2021
Granulosa cells (GC)	*FST*	Wang et al.2021
Granulosa cells (GC)	*AMHR2*	Zhao et al.2020
Oocytes (OO)	*CAPRIN2*	Pei et al. 2023
Oocytes (OO)	*CENPE*	Pei et al. 2023
Oocytes (OO)	*CENPF*	Pei et al. 2023
Oocytes (OO)	*CKAP2*	Pei et al. 2023

Oocytes (OO)	*FST*	Pei et al. 2023
Oocytes (OO)	*GRB14*	Pei et al. 2023
Oocytes (OO)	*HSPA5*	Pei et al. 2023
Oocytes (OO)	*INHA*	Pei et al. 2023
Oocytes (OO)	*MANF*	Pei et al. 2023
Oocytes (OO)	*MIS18BP1*	Pei et al. 2023
Oocytes (OO)	*NES*	Pei et al. 2023
Oocytes (OO)	*PTGES2*	Pei et al. 2023
Oocytes (OO)	*SERPINE2*	Pei et al. 2023
Oocytes (OO)	*SMC4*	Pei et al. 2023
Oocytes (OO)	*STRA6*	Pei et al. 2023
Oocytes (OO)	*TIMP1*	Pei et al. 2023
Oocytes (OO)	*TNNI3*	Pei et al. 2023
Oocytes (OO)	*TOP2A*	Pei et al. 2023
Oocytes (OO)	*UCHL1*	Pei et al. 2023
Oocytes (OO)	*GDF9*	Wagner et al.2020
Oocytes (OO)	*ZP3*	Wagner et al.2020
Oocytes (OO)	*FIGLA*	Wagner et al.2020
Oocytes (OO)	*OOSP2*	Wagner et al.2020
Oocytes (OO)	*FIGLA*	Wang et al.2020
Oocytes (OO)	*DIAPH3*	Wang et al.2020
Oocytes (OO)	*SYCP3*	Sheng et al. 2022
Stroma cells (SC)	*A2M*	Pei et al. 2023
Stroma cells (SC)	*ACTA2*	Pei et al. 2023
Stroma cells (SC)	*ACTG2*	Pei et al. 2023
Stroma cells (SC)	*ACTN1*	Pei et al. 2023
Stroma cells (SC)	*ADAMTS1*	Pei et al. 2023
Stroma cells (SC)	*ADAMTS4*	Pei et al. 2023
Stroma cells (SC)	*APOD*	Pei et al. 2023
Stroma cells (SC)	*APOE*	Pei et al. 2023
Stroma cells (SC)	*AR*	Pei et al. 2023
Stroma cells (SC)	*ARHGAP5*	Pei et al. 2023
Stroma cells (SC)	*ASH1L*	Pei et al. 2023
Stroma cells (SC)	*ASPN*	Pei et al. 2023
Stroma cells (SC)	*ATP2B1*	Pei et al. 2023
Stroma cells (SC)	*BAG3*	Pei et al. 2023
Stroma cells (SC)	*BAX*	Pei et al. 2023
Stroma cells (SC)	*BAZ2B*	Pei et al. 2023
Stroma cells (SC)	*BTG2*	Pei et al. 2023
Stroma cells (SC)	*CACYBP*	Pei et al. 2023
Stroma cells (SC)	*CCDC186*	Pei et al. 2023
Stroma cells (SC)	*CCDC80*	Pei et al. 2023

Stroma cells (SC)	*CCN2*	Pei et al. 2023
Stroma cells (SC)	*CDCA7L*	Pei et al. 2023
Stroma cells (SC)	*CITED2*	Pei et al. 2023
Stroma cells (SC)	*CKB*	Pei et al. 2023
Stroma cells (SC)	*CNN1*	Pei et al. 2023
Stroma cells (SC)	*COL12A1*	Pei et al. 2023
Stroma cells (SC)	*COL14A1*	Pei et al. 2023
Stroma cells (SC)	*COL1A2*	Pei et al. 2023
Stroma cells (SC)	*COL3A1*	Pei et al. 2023
Stroma cells (SC)	*COL4A4*	Pei et al. 2023
Stroma cells (SC)	*COL4A6*	Pei et al. 2023
Stroma cells (SC)	*COL5A1*	Pei et al. 2023
Stroma cells (SC)	*COLEC11*	Pei et al. 2023
Stroma cells (SC)	*CRYAB*	Pei et al. 2023
Stroma cells (SC)	*CRYM*	Pei et al. 2023
Stroma cells (SC)	*CST3*	Pei et al. 2023
Stroma cells (SC)	*CXCL12*	Pei et al. 2023
Stroma cells (SC)	*DCN*	Pei et al. 2023
Stroma cells (SC)	*DDIT3*	Pei et al. 2023
Stroma cells (SC)	*DES*	Pei et al. 2023
Stroma cells (SC)	*DHX57*	Pei et al. 2023
Stroma cells (SC)	*DST*	Pei et al. 2023
Stroma cells (SC)	*EGR1*	Pei et al. 2023
Stroma cells (SC)	*F3*	Pei et al. 2023
Stroma cells (SC)	*FAM71A*	Pei et al. 2023
Stroma cells (SC)	*FBLN1*	Pei et al. 2023
Stroma cells (SC)	*FBN1*	Pei et al. 2023
Stroma cells (SC)	*FHL1*	Pei et al. 2023
Stroma cells (SC)	*FILIP1L*	Pei et al. 2023
Stroma cells (SC)	*FLNA*	Pei et al. 2023
Stroma cells (SC)	*FNDC1*	Pei et al. 2023
Stroma cells (SC)	*FOS*	Pei et al. 2023
Stroma cells (SC)	*FOSB*	Pei et al. 2023
Stroma cells (SC)	*GADD45A*	Pei et al. 2023
Stroma cells (SC)	*GSN*	Pei et al. 2023
Stroma cells (SC)	*HMOX1*	Pei et al. 2023
Stroma cells (SC)	*HSP90AA1*	Pei et al. 2023
Stroma cells (SC)	*HSPA5*	Pei et al. 2023
Stroma cells (SC)	*HSPA8*	Pei et al. 2023
Stroma cells (SC)	*HSPD1*	Pei et al. 2023
Stroma cells (SC)	*HSPH1*	Pei et al. 2023
Stroma cells (SC)	*ID1*	Pei et al. 2023
Stroma cells (SC)	*ID2*	Pei et al. 2023

Stroma cells (SC)	*IER3*	Pei et al. 2023
Stroma cells (SC)	*IER5L*	Pei et al. 2023
Stroma cells (SC)	*IFRD1*	Pei et al. 2023
Stroma cells (SC)	*IGF1*	Pei et al. 2023
Stroma cells (SC)	*IGFBP3*	Pei et al. 2023
Stroma cells (SC)	*IGFBP5*	Pei et al. 2023
Stroma cells (SC)	*IGFBP6*	Pei et al. 2023
Stroma cells (SC)	*IVNS1ABP*	Pei et al. 2023
Stroma cells (SC)	*JUN*	Pei et al. 2023
Stroma cells (SC)	*KLF4*	Pei et al. 2023
Stroma cells (SC)	*LAMA1*	Pei et al. 2023
Stroma cells (SC)	*LAMB1*	Pei et al. 2023
Stroma cells (SC)	*LAMC3*	Pei et al. 2023
Stroma cells (SC)	*LMOD1*	Pei et al. 2023
Stroma cells (SC)	*LRP1*	Pei et al. 2023
Stroma cells (SC)	*LTBP4*	Pei et al. 2023
Stroma cells (SC)	*LUC7L3*	Pei et al. 2023
Stroma cells (SC)	*LUM*	Pei et al. 2023
Stroma cells (SC)	*LY6E*	Pei et al. 2023
Stroma cells (SC)	*MARCHF7*	Pei et al. 2023
Stroma cells (SC)	*MGP*	Pei et al. 2023
Stroma cells (SC)	*MRPL18*	Pei et al. 2023
Stroma cells (SC)	*MT2A*	Pei et al. 2023
Stroma cells (SC)	*MYH11*	Pei et al. 2023
Stroma cells (SC)	*MYLK*	Pei et al. 2023
Stroma cells (SC)	*MYOCD*	Pei et al. 2023
Stroma cells (SC)	*NEXN*	Pei et al. 2023
Stroma cells (SC)	*NKTR*	Pei et al. 2023
Stroma cells (SC)	*NOVA1*	Pei et al. 2023
Stroma cells (SC)	*OGN*	Pei et al. 2023
Stroma cells (SC)	*OSR2*	Pei et al. 2023
Stroma cells (SC)	*PAWR*	Pei et al. 2023
Stroma cells (SC)	*PDE5A*	Pei et al. 2023
Stroma cells (SC)	*PDLIM7*	Pei et al. 2023
Stroma cells (SC)	*PNISR*	Pei et al. 2023
Stroma cells (SC)	*PRPF39*	Pei et al. 2023
Stroma cells (SC)	*PRUNE2*	Pei et al. 2023
Stroma cells (SC)	*PTN*	Pei et al. 2023
Stroma cells (SC)	*RALGPS2*	Pei et al. 2023
Stroma cells (SC)	*RARRES1*	Pei et al. 2023
Stroma cells (SC)	*RESF1*	Pei et al. 2023
Stroma cells (SC)	*REV3L*	Pei et al. 2023
Stroma cells (SC)	*RGS4*	Pei et al. 2023

Stroma cells (SC)	*RNPC3*	Pei et al. 2023
Stroma cells (SC)	*RPL10L*	Pei et al. 2023
Stroma cells (SC)	*RPL26*	Pei et al. 2023
Stroma cells (SC)	*RRAD*	Pei et al. 2023
Stroma cells (SC)	*SERPINE2*	Pei et al. 2023
Stroma cells (SC)	*SLMAP*	Pei et al. 2023
Stroma cells (SC)	*SMOC2*	Pei et al. 2023
Stroma cells (SC)	*SPARC*	Pei et al. 2023
Stroma cells (SC)	*SPARCL1*	Pei et al. 2023
Stroma cells (SC)	*SPON1*	Pei et al. 2023
Stroma cells (SC)	*SVEP1*	Pei et al. 2023
Stroma cells (SC)	*TAGLN*	Pei et al. 2023
Stroma cells (SC)	*TGFBR3*	Pei et al. 2023
Stroma cells (SC)	*TIMP1*	Pei et al. 2023
Stroma cells (SC)	*TOB1*	Pei et al. 2023
Stroma cells (SC)	*TPM2*	Pei et al. 2023
Stroma cells (SC)	*TUBA1B*	Pei et al. 2023
Stroma cells (SC)	*TUBB2A*	Pei et al. 2023
Stroma cells (SC)	*TUT4*	Pei et al. 2023
Stroma cells (SC)	*UBB*	Pei et al. 2023
Stroma cells (SC)	*VEGFA*	Pei et al. 2023
Stroma cells (SC)	*VIPR2*	Pei et al. 2023
Stroma cells (SC)	*WFDC1*	Pei et al. 2023
Stroma cells (SC)	*ZFAND2A*	Pei et al. 2023
Stroma cells (SC)	*ZNF638*	Pei et al. 2023
Stroma cells (SC)	*ZRANB2*	Pei et al. 2023
Stroma cells (SC)	*PDGFRA*	Wagner et al.2020
Stroma cells (SC)	*DCN*	Wagner et al.2020
Stroma cells (SC)	*COL1A1*	Wagner et al.2020
Stroma cells (SC)	*COL6A1*	Wagner et al.2020
Stroma cells (SC)	*TCF21*	Wang et al.2020
Stroma cells (SC)	*DCN*	Fan et al.2019
Stroma cells (SC)	*LUM*	Fan et al.2019
Stroma cells (SC)	*GNL3*	Fan et al.2019
Stroma cells (SC)	*ARID5B*	Fan et al.2019
Stroma cells (SC)	*GPRC5A*	Fan et al.2019
Stroma cells (SC)	*TNFRSF12A*	Fan et al.2019
Stroma cells (SC)	*XBP1*	Fan et al.2019
Stroma cells (SC)	*SELK*	Fan et al.2019
Stroma cells (SC)	*COL1A1*	Sheng et al. 2022
Stroma cells (SC)	*MFAP4*	Wang et al.2021
Stroma cells (SC)	*NR2F2*	Wang et al.2021
Stroma cells (SC)	*COL1A1*	Wang et al.2021

Endothelial cells (EC)	*ABCG2*	Pei et al. 2023
Endothelial cells (EC)	*ADAMTSL5*	Pei et al. 2023
Endothelial cells (EC)	*ADGRL4*	Pei et al. 2023
Endothelial cells (EC)	*AKAP12*	Pei et al. 2023
Endothelial cells (EC)	*APOA1*	Pei et al. 2023
Endothelial cells (EC)	*ARL15*	Pei et al. 2023
Endothelial cells (EC)	*ARL4A*	Pei et al. 2023
Endothelial cells (EC)	*ASH1L*	Pei et al. 2023
Endothelial cells (EC)	*ATP13A3*	Pei et al. 2023
Endothelial cells (EC)	*ATP6*	Pei et al. 2023
Endothelial cells (EC)	*BIRC6*	Pei et al. 2023
Endothelial cells (EC)	*CA4*	Pei et al. 2023
Endothelial cells (EC)	*CAVIN2*	Pei et al. 2023
Endothelial cells (EC)	*CCL21*	Pei et al. 2023
Endothelial cells (EC)	*CD74*	Pei et al. 2023
Endothelial cells (EC)	*COBLL1*	Pei et al. 2023
Endothelial cells (EC)	*COX1*	Pei et al. 2023
Endothelial cells (EC)	*COX2*	Pei et al. 2023
Endothelial cells (EC)	*COX3*	Pei et al. 2023
Endothelial cells (EC)	*CRYBG3*	Pei et al. 2023
Endothelial cells (EC)	*CYTB*	Pei et al. 2023
Endothelial cells (EC)	*DEPP1*	Pei et al. 2023
Endothelial cells (EC)	*DIAPH2*	Pei et al. 2023
Endothelial cells (EC)	*ECE1*	Pei et al. 2023
Endothelial cells (EC)	*EFNA1*	Pei et al. 2023
Endothelial cells (EC)	*EMCN*	Pei et al. 2023
Endothelial cells (EC)	*EPAS1*	Pei et al. 2023
Endothelial cells (EC)	*F8*	Pei et al. 2023
Endothelial cells (EC)	*FABP5*	Pei et al. 2023
Endothelial cells (EC)	*FBLN2*	Pei et al. 2023
Endothelial cells (EC)	*FN1*	Pei et al. 2023
Endothelial cells (EC)	*GALNT1*	Pei et al. 2023
Endothelial cells (EC)	*GAS6*	Pei et al. 2023
Endothelial cells (EC)	*GJA4*	Pei et al. 2023
Endothelial cells (EC)	*GNG11*	Pei et al. 2023
Endothelial cells (EC)	*GPM6A*	Pei et al. 2023
Endothelial cells (EC)	*HLA-DRA*	Pei et al. 2023
Endothelial cells (EC)	*IGFBP7*	Pei et al. 2023
Endothelial cells (EC)	*IRF1*	Pei et al. 2023
Endothelial cells (EC)	*ITGA5*	Pei et al. 2023
Endothelial cells (EC)	*JAG1*	Pei et al. 2023
Endothelial cells (EC)	*JAG2*	Pei et al. 2023
Endothelial cells (EC)	*KANK3*	Pei et al. 2023

Endothelial cells (EC)	*KDR*	Pei et al. 2023
Endothelial cells (EC)	*KLF2*	Pei et al. 2023
Endothelial cells (EC)	*LIMA1*	Pei et al. 2023
Endothelial cells (EC)	*LPAR6*	Pei et al. 2023
Endothelial cells (EC)	*LUZP1*	Pei et al. 2023
Endothelial cells (EC)	*MACF1*	Pei et al. 2023
Endothelial cells (EC)	*MECOM*	Pei et al. 2023
Endothelial cells (EC)	*MMRN1*	Pei et al. 2023
Endothelial cells (EC)	*NBEA*	Pei et al. 2023
Endothelial cells (EC)	*ND3*	Pei et al. 2023
Endothelial cells (EC)	*ND5*	Pei et al. 2023
Endothelial cells (EC)	*NES*	Pei et al. 2023
Endothelial cells (EC)	*NFIB*	Pei et al. 2023
Endothelial cells (EC)	*NFKBIZ*	Pei et al. 2023
Endothelial cells (EC)	*NOSTRIN*	Pei et al. 2023
Endothelial cells (EC)	*NR2F1*	Pei et al. 2023
Endothelial cells (EC)	*PARD6G*	Pei et al. 2023
Endothelial cells (EC)	*PKHD1L1*	Pei et al. 2023
Endothelial cells (EC)	*PROX1*	Pei et al. 2023
Endothelial cells (EC)	*PRRC2C*	Pei et al. 2023
Endothelial cells (EC)	*PTPRM*	Pei et al. 2023
Endothelial cells (EC)	*RAB11FIP1*	Pei et al. 2023
Endothelial cells (EC)	*RAMP2*	Pei et al. 2023
Endothelial cells (EC)	*RELN*	Pei et al. 2023
Endothelial cells (EC)	*RND1*	Pei et al. 2023
Endothelial cells (EC)	*ROBO4*	Pei et al. 2023
Endothelial cells (EC)	*SEMA3D*	Pei et al. 2023
Endothelial cells (EC)	*SPTBN1*	Pei et al. 2023
Endothelial cells (EC)	*STAB1*	Pei et al. 2023
Endothelial cells (EC)	*SYNE2*	Pei et al. 2023
Endothelial cells (EC)	*TFPI2*	Pei et al. 2023
Endothelial cells (EC)	*TM4SF18*	Pei et al. 2023
Endothelial cells (EC)	*TNFSF10*	Pei et al. 2023
Endothelial cells (EC)	*UACA*	Pei et al. 2023
Endothelial cells (EC)	*UTRN*	Pei et al. 2023
Endothelial cells (EC)	*VWF*	Pei et al. 2023
Endothelial cells (EC)	*XDH*	Pei et al. 2023
Endothelial cells (EC)	*ZNF521*	Pei et al. 2023
Endothelial cells (EC)	*VWF*	Wagner et al.2020
Endothelial cells (EC)	*CDH5*	Wagner et al.2020
Endothelial cells (EC)	*FLI1*	Wagner et al.2020
Endothelial cells (EC)	*CD34*	Wagner et al.2020
Endothelial cells (EC)	*ERG*	Wang et al.2020

Endothelial cells (EC)	*VWF*	Fan et al.2019
Endothelial cells (EC)	*CLDN5*	Fan et al.2019
Endothelial cells (EC)	*CD34*	Fan et al.2019
Endothelial cells (EC)	*PECAM1*	Fan et al.2019
Endothelial cells (EC)	*CCL14*	Fan et al.2019
Endothelial cells (EC)	*SOCS3*	Fan et al.2019
Endothelial cells (EC)	*TXNIP*	Fan et al.2019
Endothelial cells (EC)	*CTGF*	Fan et al.2019
Endothelial cells (EC)	*TM4SF1*	Fan et al.2019
Endothelial cells (EC)	*NNMT*	Fan et al.2019
Endothelial cells (EC)	*ANGPT2*	Fan et al.2019
Endothelial cells (EC)	*EGFL7*	Fan et al.2019
Endothelial cells (EC)	*CCL21*	Fan et al.2019
Endothelial cells (EC)	*TFF3*	Fan et al.2019
Endothelial cells (EC)	*CDH5*	Sheng et al. 2022
Endothelial cells (EC)	*APLNR*	Wang et al.2021
Endothelial cells (EC)	*PECAM1*	Wang et al.2021
Endothelial cells (EC)	*EGFL7*	Wang et al.2021
Endothelial cells (EC)	*PECAM1*	Zhao et al.2020
Immune cells	*CD69*	Wagner et al.2020
Immune cells	*ITGB2*	Wagner et al.2020
Immune cells	*CD2*	Wagner et al.2020
Immune cells	*CD3G*	Wagner et al.2020
Immune cells	*CD8A*	Wagner et al.2020
Immune cells	*CD14*	Wagner et al.2020
Immune cells	*HLA-DRA*	Wagner et al.2020
Immune cells	*B2M*	Wagner et al.2020
Immune cells	*HLA-DQB1*	Wagner et al.2020
Immune cells	*CCL5*	Wagner et al.2020
Immune cells	*CXCR4*	Wagner et al.2020
Immune cells	*CCL4*	Wagner et al.2020
Immune cells	*REL*	Wang et al.2020
Immune cells	*CD53*	Fan et al.2019
Immune cells	*CXCR4*	Fan et al.2019
Immune cells	*PTPRC*	Sheng et al. 2022
Immune cells	*TYROBP*	Wang et al.2021
Immune cells	*CD52*	Wang et al.2021
Immune cells	*PLAC8*	Wang et al.2021
Perivascular cells	*MYH11*	Wagner et al.2020
Perivascular cells	*MCAM*	Wagner et al.2020
Perivascular cells	*RGS5*	Wagner et al.2020
Perivascular cells	*RERGL*	Wagner et al.2020
Perivascular cells	*TAGLN*	Wagner et al.2020

Perivascular cells	*DDX4*	Wagner et al.2020
Perivascular cells	*PDE3A*	Wagner et al.2020
Perivascular cells	*EBF1*	Wagner et al.2020
Perivascular cells	*DLC1*	Wagner et al.2020
Perivascular cells	*PRKG1*	Wagner et al.2020
Less differentiated GC	*WT1*	Sheng et al. 2022
Less differentiated GC	*FOXL2*	Sheng et al. 2022
Cumulus GC	*SLC38A3*	Sheng et al. 2022
Cumulus GC	*AMH*	Sheng et al. 2022
Mural GC	*CYP19A1(CYP19)*	Sheng et al. 2022
Steroidogenic GC	*CYP11A1*	Sheng et al. 2022
Steroidogenic GC	*LHCGR*	Sheng et al. 2022

Table S11. The expression of genes characterized from oocytes in ovary.

gene	p_val	Log_2FC	pct.1	pct.2	p_val_adj	cluster
DIAPH3	0	2.38	0.929	0.106	0	oocytes
CENPE	0	1.66	0.737	0.059	0	oocytes
MIS18BP1	0	1.65	0.799	0.043	0	oocytes
TRHDE	0	1.54	0.664	0.198	0	oocytes
CENPF	0	1.48	0.7	0.113	0	oocytes
IGSF11	0	1.40	0.842	0.288	0	oocytes
CIT	0	1.39	0.748	0.056	0	oocytes
GPC5	0	1.36	0.951	0.737	0	oocytes
KIF23	0	1.35	0.724	0.043	0	oocytes
TOP2A	0	1.29	0.781	0.142	0	oocytes
TPX2	0	1.28	0.696	0.055	0	oocytes
KIF4A	0	1.28	0.709	0.025	0	oocytes
ECT2	0	1.24	0.666	0.037	0	oocytes
HS6ST2	0	1.24	0.918	0.446	0	oocytes
NDC80	0	1.24	0.666	0.026	0	oocytes

Table S12. Statistical information on genes of all clusters in the ovaries of high-fertility (HF) and low-fertility (LF)Tibetan sheep

gene	log_2FC	p_val	p_val_adj	cluster
BMPR1B	0.99	6.75E-275	1.20E-270	5
LHCGR	1.33	2.69E-239	4.78E-235	5
BMPR1B	0.59	1.08E-235	1.92E-231	0
BMPR1B	0.59	1.08E-235	1.92E-231	2
BMPR1B	0.49	1.64E-231	2.93E-227	1
BMPR1B	0.93	3.37E-119	5.99E-115	14
BMPR1B	0.32	2.72E-108	4.84E-104	3
BMPR1B	0.54	1.76E-92	3.13E-88	7
BMPR1B	0.84	3.20E-86	5.70E-82	9
BMPR1B	0.37	8.16E-66	1.45E-61	8
LHCGR	1.30	8.01E-44	1.42E-39	14
BMPR1B	0.26	2.04E-21	3.63E-17	6
BMPR1B	0.59	9.38E-21	1.67E-16	20
BMPR1B	0.26	6.50E-19	1.16E-14	17
BMPR1B	0.42	1.32E-12	2.35E-08	12
BMPR1B	0.57	2.84E-11	5.05E-07	10
BMPR1B	-0.29	3.17E-09	5.63E-05	19

Table S13. The top 20 genes at highly expressed level in LF and HF groups

Gene ID	LO1	LO2	LO3	HO1	HO2	HO3
MGAT4C	1.7601624	0.8546282	0.95978	8.8480506	8.2690126	3.1317759
ROBO2	11.430227	12.781736	15.418718	24.478554	26.484012	38.483119
PRKG1	8.9149937	5.9059957	14.887813	18.030855	27.558118	15.380348
DLG2	4.9707465	3.959856	6.6542066	15.34747	17.593986	11.983684
PCDH7	5.6999873	3.4647847	11.52049	9.7797275	29.82145	8.9646578
PLXDC2	5.9430387	4.3836117	9.0230575	14.59232	19.35253	12.382974
LTBP1	6.4688094	3.7521719	9.2064332	12.739218	18.85661	13.280756
LRP1B	3.2815613	0.8389486	1.5851623	8.1146839	17.735481	1.4597673
DGKB	4.9649584	1.6408669	6.1390197	12.260337	14.252073	7.3236156
SGCD	6.8927307	4.6880549	7.3610525	15.081108	10.650137	12.218882
DLC1	4.3293694	1.5301621	6.9588698	7.4539309	13.208833	10.928066
NAALADL2	7.6111613	6.0944061	7.3929348	12.795629	15.549496	11.134405
FBXL7	6.794636	13.779923	7.698196	14.313829	13.727401	18.533986
BMPR1B	10.870887	16.657097	9.6850149	20.974361	15.294037	18.843164
BMX	0.0380754	0.0216894	0.1603797	0.1880912	0.5296843	0.6901174
BRINP3	0.4428546	0.1782412	0.3963012	1.5794276	2.8335438	0.9418843
ADAMTSL1	0.5084791	0.3120811	0.6572937	1.3840196	2.2050405	1.7926342
IGF1	0.8589418	0.1429093	1.3238681	2.0113742	2.1990028	0.9437187
MITF	3.2200744	4.2815921	4.6496555	7.0140101	7.1169337	7.5704492
ANO6	3.1463936	3.7085451	3.3001655	6.6846822	6.6331719	5.201692

Table S14. Statistical information on the function of biological processes for all clusters

GO ID	Description	Rich Ratio	P value	Q value	Cluster
GO:0006412	translation	0.25	5.33E-54	8.55E-51	0
GO:0015986	ATP synthesis coupled proton transport	0.63	1.07E-14	8.59E-12	0
GO:0002181	cytoplasmic translation	0.86	3.60E-09	1.92E-06	0
GO:0006754	ATP biosynthetic process	0.47	8.09E-09	3.24E-06	0
GO:0006457	protein folding	0.18	3.78E-08	1.21E-05	0
GO:0006414	translational elongation	0.19	1.77E-06	4.73E-04	0
GO:0042776	mitochondrial ATP synthesis coupled proton transport	0.80	3.22E-06	7.37E-04	0
GO:0006119	oxidative phosphorylation	0.35	4.98E-06	9.93E-04	0
GO:0050821	protein stabilization	0.12	5.57E-06	9.93E-04	0
GO:0006122	mitochondrial electron transport, ubiquinol to cytochrome c	0.67	9.43E-06	1.51E-03	0
GO:0006413	translational initiation	0.15	1.44E-05	2.09E-03	0
GO:0006123	mitochondrial electron transport, cytochrome c to oxygen	0.38	1.98E-05	2.65E-03	0
GO:0006749	glutathione metabolic process	0.17	4.19E-05	5.17E-03	0
GO:0071230	cellular response to amino acid stimulus	0.19	6.04E-05	6.92E-03	0
GO:0051603	proteolysis involved in cellular protein catabolic process	0.19	7.27E-05	7.77E-03	0
GO:1901798	positive regulation of signal transduction by p53 class mediator	0.75	9.08E-05	9.10E-03	0
GO:0032981	mitochondrial respiratory chain complex I assembly	0.18	1.03E-04	9.28E-03	0
GO:0098869	cellular oxidant detoxification	0.15	1.04E-04	9.28E-03	0
GO:0042026	protein refolding	0.40	1.20E-04	1.02E-02	0
GO:1900026	positive regulation of substrate adhesion-dependent cell spreading	0.19	2.11E-04	1.69E-02	0
GO:0007155	cell adhesion	0.07	5.93E-07	5.73E-04	1
GO:0030324	lung development	0.14	8.10E-07	5.73E-04	1
GO:0035108	limb morphogenesis	0.26	3.02E-06	1.32E-03	1
GO:0071230	cellular response to amino acid stimulus	0.19	3.73E-06	1.32E-03	1

GO:0030198	extracellular matrix organization	0.13	6.30E-06	1.78E-03	1
GO:0007160	cell-matrix adhesion	0.13	1.42E-05	3.35E-03	1
GO:0045669	positive regulation of osteoblast differentiation	0.15	2.04E-05	3.97E-03	1
GO:0048598	embryonic morphogenesis	0.40	2.24E-05	3.97E-03	1
GO:0048565	digestive tract development	0.25	2.63E-05	4.14E-03	1
GO:0008285	negative regulation of cell proliferation	0.06	2.96E-05	4.18E-03	1
GO:0030154	cell differentiation	0.07	4.28E-05	5.50E-03	1
GO:0048568	embryonic organ development	0.22	5.46E-05	6.44E-03	1
GO:0007169	transmembrane receptor protein tyrosine kinase signaling pathway	0.13	6.71E-05	7.30E-03	1
GO:0007411	axon guidance	0.09	7.32E-05	7.40E-03	1
GO:0009953	dorsal/ventral pattern formation	0.19	1.23E-04	1.06E-02	1
GO:0042475	odontogenesis of dentin-containing tooth	0.14	1.33E-04	1.06E-02	1
GO:0048566	embryonic digestive tract development	0.27	1.35E-04	1.06E-02	1
GO:0050770	regulation of axonogenesis	0.27	1.35E-04	1.06E-02	1
GO:0045944	positive regulation of transcription by RNA polymerase II	0.04	1.47E-04	1.10E-02	1
GO:0060831	smoothened signaling pathway involved in dorsal/ventral neural tube patterning	0.43	2.10E-04	1.49E-02	1
GO:0016310	phosphorylation	0.06	4.01E-05	4.49E-02	2
GO:0017157	regulation of exocytosis	0.33	7.37E-05	4.49E-02	2
GO:0050790	regulation of catalytic activity	0.07	5.16E-05	4.49E-02	2
GO:0050790	regulation of catalytic activity	0.07	4.06E-08	6.56E-05	3
GO:0032869	cellular response to insulin stimulus	0.18	8.51E-06	6.88E-03	3
GO:0030335	positive regulation of cell migration	0.09	4.91E-05	2.64E-02	3
GO:0008286	insulin receptor signaling pathway	0.17	1.45E-04	3.36E-02	3
GO:0015810	aspartate transmembrane transport	0.60	1.23E-04	3.36E-02	3
GO:0016310	phosphorylation	0.05	9.88E-05	3.36E-02	3
GO:0016477	cell migration	0.09	1.38E-04	3.36E-02	3
GO:0007264	small GTPase mediated signal transduction	0.10	3.56E-04	4.22E-02	3

GO:0007411	axon guidance	0.09	4.17E-04	4.22E-02	3
GO:0010718	positive regulation of epithelial to mesenchymal transition	0.17	5.14E-04	4.22E-02	3
GO:0017157	regulation of exocytosis	0.27	3.30E-04	4.22E-02	3
GO:0021636	trigeminal nerve morphogenesis	1.00	5.48E-04	4.22E-02	3
GO:0030505	inorganic diphosphate transport	1.00	5.48E-04	4.22E-02	3
GO:0031022	nuclear migration along microfilament	1.00	5.48E-04	4.22E-02	3
GO:0043490	malate-aspartate shuttle	1.00	5.48E-04	4.22E-02	3
GO:0043547	positive regulation of GTPase activity	0.08	3.37E-04	4.22E-02	3
GO:0045944	positive regulation of transcription by RNA polymerase II	0.04	4.28E-04	4.22E-02	3
GO:0046328	regulation of JNK cascade	0.29	2.47E-04	4.22E-02	3
GO:0048009	insulin-like growth factor receptor signaling pathway	0.43	4.17E-04	4.22E-02	3
GO:0048340	paraxial mesoderm morphogenesis	1.00	5.48E-04	4.22E-02	3
GO:0001525	angiogenesis	0.20	1.12E-12	2.38E-09	4
GO:0001569	branching involved in blood vessel morphogenesis	0.38	1.65E-08	1.75E-05	4
GO:0001570	vasculogenesis	0.26	3.03E-08	1.91E-05	4
GO:0043542	endothelial cell migration	0.42	3.61E-08	1.91E-05	4
GO:0045766	positive regulation of angiogenesis	0.16	4.03E-07	1.70E-04	4
GO:0030335	positive regulation of cell migration	0.12	5.54E-07	1.73E-04	4
GO:0061028	establishment of endothelial barrier	0.50	5.74E-07	1.73E-04	4
GO:0035556	intracellular signal transduction	0.09	6.90E-07	1.83E-04	4
GO:0002040	sprouting angiogenesis	0.35	1.19E-06	2.66E-04	4
GO:0006468	protein phosphorylation	0.07	1.38E-06	2.66E-04	4
GO:0007166	cell surface receptor signaling pathway	0.13	1.31E-06	2.66E-04	4
GO:0002042	cell migration involved in sprouting angiogenesis	0.43	1.77E-06	3.12E-04	4
GO:0048870	cell motility	0.40	2.88E-06	4.35E-04	4
GO:0090050	positive regulation of cell migration involved in sprouting angiogenesis	0.40	2.88E-06	4.35E-04	4
GO:0045765	regulation of angiogenesis	0.30	3.47E-06	4.90E-04	4
GO:0008360	regulation of cell shape	0.15	5.20E-06	6.87E-04	4

GO:0050790	regulation of catalytic activity	0.07	7.12E-06	8.86E-04	4
GO:0045669	positive regulation of osteoblast differentiation	0.20	7.73E-06	9.08E-04	4
GO:0001934	positive regulation of protein phosphorylation	0.12	8.61E-06	9.17E-04	4
GO:0098609	cell-cell adhesion	0.14	8.67E-06	9.17E-04	4
GO:0016126	sterol biosynthetic process	0.43	8.29E-08	1.15E-04	5
GO:0006694	steroid biosynthetic process	0.27	2.68E-07	1.43E-04	5
GO:0008203	cholesterol metabolic process	0.17	3.10E-07	1.43E-04	5
GO:0008202	steroid metabolic process	0.19	2.92E-06	1.01E-03	5
GO:0006695	cholesterol biosynthetic process	0.26	1.68E-05	4.63E-03	5
GO:0008299	isoprenoid biosynthetic process	0.33	4.44E-05	1.02E-02	5
GO:0010811	positive regulation of cell-substrate adhesion	0.19	1.04E-04	2.04E-02	5
GO:0032964	collagen biosynthetic process	0.43	1.88E-04	3.25E-02	5
GO:0030199	collagen fibril organization	0.16	2.40E-04	3.32E-02	5
GO:0051897	positive regulation of protein kinase B signaling	0.10	2.28E-04	3.32E-02	5
GO:0010899	regulation of phosphatidylcholine catabolic process	1.00	3.19E-04	3.39E-02	5
GO:0021955	central nervous system neuron axonogenesis	0.38	2.97E-04	3.39E-02	5
GO:0097324	melanocyte migration	1.00	3.19E-04	3.39E-02	5
GO:0045725	positive regulation of glycogen biosynthetic process	0.33	4.40E-04	4.34E-02	5
GO:0043588	skin development	0.14	4.83E-04	4.45E-02	5
GO:0001568	blood vessel development	0.13	6.99E-04	4.67E-02	5
GO:0007160	cell-matrix adhesion	0.10	6.94E-04	4.67E-02	5
GO:0010812	negative regulation of cell-substrate adhesion	0.30	6.21E-04	4.67E-02	5
GO:0032765	positive regulation of mast cell cytokine production	0.67	9.47E-04	4.67E-02	5
GO:0035722	interleukin-12-mediated signaling pathway	0.67	9.47E-04	4.67E-02	5
GO:0007411	axon guidance	0.14	2.34E-07	3.65E-04	6
GO:0071230	cellular response to amino acid stimulus	0.22	1.08E-06	8.43E-04	6
GO:0030324	lung development	0.14	4.64E-06	2.42E-03	6
GO:0007155	cell adhesion	0.07	1.15E-05	4.49E-03	6

GO:0007507	heart development	0.09	3.55E-05	1.11E-02	6
GO:0008016	regulation of heart contraction	0.33	1.09E-04	2.85E-02	6
GO:0007399	nervous system development	0.08	1.87E-04	4.17E-02	6
GO:0007010	cytoskeleton organization	0.10	6.08E-04	5.00E-02	6
GO:0009954	proximal/distal pattern formation	0.24	4.80E-04	5.00E-02	6
GO:0030036	actin cytoskeleton organization	0.09	3.68E-04	5.00E-02	6
GO:0032355	response to estradiol	0.43	3.73E-04	5.00E-02	6
GO:0035295	tube development	1.00	5.08E-04	5.00E-02	6
GO:0045184	establishment of protein localization	0.19	3.05E-04	5.00E-02	6
GO:0045669	positive regulation of osteoblast differentiation	0.13	5.53E-04	5.00E-02	6
GO:0048681	negative regulation of axon regeneration	0.38	5.86E-04	5.00E-02	6
GO:0050919	negative chemotaxis	0.27	2.86E-04	5.00E-02	6
GO:1900119	positive regulation of execution phase of apoptosis	0.38	5.86E-04	5.00E-02	6
GO:1903225	negative regulation of endodermal cell differentiation	1.00	5.08E-04	5.00E-02	6
GO:2000544	regulation of endothelial cell chemotaxis to fibroblast growth factor	1.00	5.08E-04	5.00E-02	6
GO:0007155	cell adhesion	0.06	1.02E-07	1.11E-04	7
GO:0040037	negative regulation of fibroblast growth factor receptor signaling pathway	0.36	1.01E-05	5.49E-03	7
GO:0016477	cell migration	0.07	4.27E-05	1.55E-02	7
GO:0021740	principal sensory nucleus of trigeminal nerve development	1.00	1.84E-04	3.70E-02	7
GO:0030154	cell differentiation	0.05	2.20E-04	3.70E-02	7
GO:0030505	inorganic diphosphate transport	1.00	1.84E-04	3.70E-02	7
GO:0035108	limb morphogenesis	0.17	2.39E-04	3.70E-02	7
GO:0007411	axon guidance	0.07	3.30E-04	4.48E-02	7
GO:0003007	heart morphogenesis	0.13	8.81E-04	4.65E-02	7
GO:0007223	Wnt signaling pathway, calcium modulating pathway	0.67	5.46E-04	4.65E-02	7
GO:0007275	multicellular organism development	0.08	7.11E-04	4.65E-02	7
GO:0007442	hindgut morphogenesis	0.67	5.46E-04	4.65E-02	7

GO:0009887	animal organ morphogenesis	0.10	6.22E-04	4.65E-02	7
GO:0016525	negative regulation of angiogenesis	0.09	1.04E-03	4.65E-02	7
GO:0034138	toll-like receptor 3 signaling pathway	0.50	1.08E-03	4.65E-02	7
GO:0045599	negative regulation of fat cell differentiation	0.13	8.81E-04	4.65E-02	7
GO:0048566	embryonic digestive tract development	0.20	9.95E-04	4.65E-02	7
GO:0048706	embryonic skeletal system development	0.12	1.11E-03	4.65E-02	7
GO:0051781	positive regulation of cell division	0.14	6.01E-04	4.65E-02	7
GO:0051928	positive regulation of calcium ion transport	0.25	4.96E-04	4.65E-02	7
GO:0007049	cell cycle	0.29	2.04E-25	4.09E-22	8
GO:0051301	cell division	0.30	6.69E-23	6.71E-20	8
GO:0007059	chromosome segregation	0.33	1.18E-10	7.09E-08	8
GO:0007099	centriole replication	0.63	1.41E-10	7.09E-08	8
GO:0051298	centrosome duplication	0.62	1.29E-08	5.17E-06	8
GO:0034501	protein localization to kinetochore	0.70	3.03E-08	1.01E-05	8
GO:0000278	mitotic cell cycle	0.26	4.80E-08	1.38E-05	8
GO:0006281	DNA repair	0.14	1.34E-07	3.13E-05	8
GO:0032467	positive regulation of cytokinesis	0.41	1.56E-07	3.13E-05	8
GO:0050790	regulation of catalytic activity	0.11	1.41E-07	3.13E-05	8
GO:0006260	DNA replication	0.20	3.23E-07	5.88E-05	8
GO:0006974	cellular response to DNA damage stimulus	0.12	4.14E-07	6.92E-05	8
GO:0000724	double-strand break repair via homologous recombination	0.24	4.68E-07	7.07E-05	8
GO:0030261	chromosome condensation	0.67	4.94E-07	7.07E-05	8
GO:0007018	microtubule-based movement	0.20	1.25E-06	1.67E-04	8
GO:0000086	G2/M transition of mitotic cell cycle	0.36	2.26E-06	2.83E-04	8
GO:0051988	regulation of attachment of spindle microtubules to kinetochore	0.71	2.98E-06	3.51E-04	8
GO:0007094	mitotic spindle assembly checkpoint	0.41	3.76E-06	4.18E-04	8
GO:0007076	mitotic chromosome condensation	0.50	4.85E-06	5.12E-04	8
GO:0007095	mitotic G2 DNA damage checkpoint	0.32	6.81E-06	6.83E-04	8

GO:0030324	lung development	0.11	3.98E-07	4.08E-04	9
GO:0071230	cellular response to amino acid stimulus	0.14	2.17E-05	1.11E-02	9
GO:0030198	extracellular matrix organization	0.08	5.79E-05	1.25E-02	9
GO:0035108	limb morphogenesis	0.17	6.07E-05	1.25E-02	9
GO:0050679	positive regulation of epithelial cell proliferation	0.12	4.68E-05	1.25E-02	9
GO:0002053	positive regulation of mesenchymal cell proliferation	0.18	5.22E-04	2.11E-02	9
GO:0003007	heart morphogenesis	0.13	2.31E-04	2.11E-02	9
GO:0007155	cell adhesion	0.03	5.00E-04	2.11E-02	9
GO:0007442	hindgut morphogenesis	0.67	2.69E-04	2.11E-02	9
GO:0008284	positive regulation of cell proliferation	0.03	4.74E-04	2.11E-02	9
GO:0030154	cell differentiation	0.04	3.94E-04	2.11E-02	9
GO:0038179	neurotrophin signaling pathway	0.67	2.69E-04	2.11E-02	9
GO:0040037	negative regulation of fibroblast growth factor receptor signaling pathway	0.27	1.32E-04	2.11E-02	9
GO:0048286	lung alveolus development	0.13	1.78E-04	2.11E-02	9
GO:0048566	embryonic digestive tract development	0.20	3.54E-04	2.11E-02	9
GO:0048752	semicircular canal morphogenesis	0.50	5.34E-04	2.11E-02	9
GO:0051145	smooth muscle cell differentiation	0.25	1.75E-04	2.11E-02	9
GO:0055012	ventricular cardiac muscle cell differentiation	0.50	5.34E-04	2.11E-02	9
GO:0060157	urinary bladder development	0.67	2.69E-04	2.11E-02	9
GO:0060513	prostatic bud formation	0.50	5.34E-04	2.11E-02	9
GO:0060271	cilium assembly	0.48	5.15E-48	1.18E-44	10
GO:0003341	cilium movement	0.76	8.49E-20	9.70E-17	10
GO:1905515	non-motile cilium assembly	0.58	1.84E-19	1.41E-16	10
GO:0030030	cell projection organization	0.45	5.96E-15	2.63E-12	10
GO:0030317	flagellated sperm motility	0.38	5.49E-15	2.63E-12	10
GO:0035082	axoneme assembly	0.92	6.90E-15	2.63E-12	10
GO:0007368	determination of left/right symmetry	0.44	1.01E-14	3.29E-12	10
GO:0044458	motile cilium assembly	0.72	1.97E-13	5.64E-11	10

GO:0007018	microtubule-based movement	0.32	4.98E-12	1.27E-09	10
GO:0061512	protein localization to cilium	0.54	4.25E-11	9.72E-09	10
GO:0036159	inner dynein arm assembly	0.75	7.01E-10	1.34E-07	10
GO:0070286	axonemal dynein complex assembly	0.75	7.01E-10	1.34E-07	10
GO:0060287	epithelial cilium movement involved in determination of left/right asymmetry	1.00	1.29E-09	2.27E-07	10
GO:0003351	epithelial cilium movement	0.69	2.17E-09	3.21E-07	10
GO:0007288	sperm axoneme assembly	0.52	2.25E-09	3.21E-07	10
GO:0036158	outer dynein arm assembly	0.69	2.17E-09	3.21E-07	10
GO:0035721	intraciliary retrograde transport	0.80	2.82E-09	3.80E-07	10
GO:0090660	cerebrospinal fluid circulation	0.88	9.85E-09	1.25E-06	10
GO:0008589	regulation of smoothened signaling pathway	0.62	6.98E-08	8.40E-06	10
GO:0042073	intraciliary transport	0.50	1.15E-07	1.32E-05	10
GO:0099560	synaptic membrane adhesion	0.36	2.81E-05	4.79E-02	11
GO:0035556	intracellular signal transduction	0.17	2.29E-21	4.44E-18	12
GO:0050790	regulation of catalytic activity	0.12	2.78E-15	2.70E-12	12
GO:0050852	T cell receptor signaling pathway	0.31	1.20E-11	7.76E-09	12
GO:0016310	phosphorylation	0.08	1.58E-10	7.68E-08	12
GO:0006468	protein phosphorylation	0.08	1.91E-09	7.05E-07	12
GO:0030217	T cell differentiation	0.34	2.18E-09	7.05E-07	12
GO:0007264	small GTPase mediated signal transduction	0.18	6.00E-09	1.67E-06	12
GO:0042110	T cell activation	0.41	1.09E-08	2.35E-06	12
GO:0050870	positive regulation of T cell activation	0.50	1.04E-08	2.35E-06	12
GO:0043547	positive regulation of GTPase activity	0.14	5.53E-08	1.07E-05	12
GO:0045954	positive regulation of natural killer cell mediated cytotoxicity	0.47	1.65E-07	2.68E-05	12
GO:2000114	regulation of establishment of cell polarity	0.47	1.65E-07	2.68E-05	12
GO:0002250	adaptive immune response	0.22	3.75E-07	5.60E-05	12
GO:0045061	thymic T cell selection	1.00	1.02E-06	1.42E-04	12

GO:0002376	immune system process	0.14	1.30E-06	1.68E-04	12
GO:0042102	positive regulation of T cell proliferation	0.25	1.38E-06	1.68E-04	12
GO:0019885	antigen processing and presentation of endogenous peptide antigen via MHC class I	0.63	1.67E-06	1.91E-04	12
GO:0032743	positive regulation of interleukin-2 production	0.29	1.79E-06	1.93E-04	12
GO:0030890	positive regulation of B cell proliferation	0.27	3.18E-06	3.25E-04	12
GO:0030866	cortical actin cytoskeleton organization	0.32	3.61E-06	3.51E-04	12
GO:0050853	B cell receptor signaling pathway	0.61	1.22E-15	3.35E-12	13
GO:0035556	intracellular signal transduction	0.17	2.31E-14	3.18E-11	13
GO:0043547	positive regulation of GTPase activity	0.23	1.07E-12	9.81E-10	13
GO:0050790	regulation of catalytic activity	0.14	6.12E-11	4.21E-08	13
GO:0019882	antigen processing and presentation	0.36	3.46E-09	1.90E-06	13
GO:0016310	phosphorylation	0.10	4.89E-09	2.24E-06	13
GO:0006955	immune response	0.16	9.75E-09	3.83E-06	13
GO:0030593	neutrophil chemotaxis	0.44	1.36E-08	4.69E-06	13
GO:0002504	antigen processing and presentation of peptide or polysaccharide antigen via MHC class II	0.57	1.04E-07	3.14E-05	13
GO:0071222	cellular response to lipopolysaccharide	0.23	1.14E-07	3.14E-05	13
GO:0030890	positive regulation of B cell proliferation	0.37	1.32E-07	3.30E-05	13
GO:0007264	small GTPase mediated signal transduction	0.20	2.30E-07	4.86E-05	13
GO:0016477	cell migration	0.18	2.18E-07	4.86E-05	13
GO:0070374	positive regulation of ERK1 and ERK2 cascade	0.18	3.83E-07	7.53E-05	13
GO:0002250	adaptive immune response	0.26	9.18E-07	1.58E-04	13
GO:0050731	positive regulation of peptidyl-tyrosine phosphorylation	0.23	8.86E-07	1.58E-04	13
GO:0006897	endocytosis	0.17	1.20E-06	1.95E-04	13
GO:0034446	substrate adhesion-dependent cell spreading	0.31	2.70E-06	4.12E-04	13
GO:0031623	receptor internalization	0.35	3.29E-06	4.76E-04	13
GO:0006468	protein phosphorylation	0.10	3.71E-06	5.11E-04	13
GO:0006468	protein phosphorylation	0.06	2.57E-06	5.07E-03	14

GO:0050790	regulation of catalytic activity	0.07	2.77E-05	1.82E-02	14
GO:0051726	regulation of cell cycle	0.10	2.77E-05	1.82E-02	14
GO:0016310	phosphorylation	0.05	5.44E-05	2.69E-02	14
GO:0006469	negative regulation of protein kinase activity	0.15	2.36E-04	4.23E-02	14
GO:0038083	peptidyl-tyrosine autophosphorylation	0.33	2.32E-04	4.23E-02	14
GO:0042541	hemoglobin biosynthetic process	0.60	1.97E-04	4.23E-02	14
GO:0045822	negative regulation of heart contraction	0.60	1.97E-04	4.23E-02	14
GO:0045944	positive regulation of transcription by RNA polymerase II	0.05	1.23E-04	4.23E-02	14
GO:0060019	radial glial cell differentiation	0.60	1.97E-04	4.23E-02	14
GO:1990456	mitochondrion-endoplasmic reticulum membrane tethering	0.60	1.97E-04	4.23E-02	14
GO:0006710	androgen catabolic process	1.00	7.51E-04	4.78E-02	14
GO:0007155	cell adhesion	0.06	4.82E-04	4.78E-02	14
GO:0008285	negative regulation of cell proliferation	0.07	6.86E-04	4.78E-02	14
GO:0009791	post-embryonic development	0.13	3.46E-04	4.78E-02	14
GO:0016199	axon midline choice point recognition	1.00	7.51E-04	4.78E-02	14
GO:0021636	trigeminal nerve morphogenesis	1.00	7.51E-04	4.78E-02	14
GO:0021785	branchiomotor neuron axon guidance	0.50	3.85E-04	4.78E-02	14
GO:0030007	cellular potassium ion homeostasis	0.50	3.85E-04	4.78E-02	14
GO:0036376	sodium ion export across plasma membrane	0.43	6.61E-04	4.78E-02	14
GO:0050790	regulation of catalytic activity	0.10	4.70E-10	1.03E-06	15
GO:0001525	angiogenesis	0.17	4.44E-09	4.88E-06	15
GO:0007264	small GTPase mediated signal transduction	0.17	1.05E-07	7.69E-05	15
GO:0035556	intracellular signal transduction	0.10	6.92E-07	3.36E-04	15
GO:0043547	positive regulation of GTPase activity	0.13	7.64E-07	3.36E-04	15
GO:0008285	negative regulation of cell proliferation	0.10	1.36E-06	5.00E-04	15
GO:0050804	modulation of chemical synaptic transmission	0.21	3.25E-06	1.02E-03	15
GO:0010628	positive regulation of gene expression	0.09	5.07E-06	1.39E-03	15
GO:0014068	positive regulation of phosphatidylinositol 3-kinase signaling	0.20	7.10E-06	1.74E-03	15

GO:0007155	cell adhesion	0.09	8.67E-06	1.91E-03	15
GO:0010634	positive regulation of epithelial cell migration	0.28	1.45E-05	2.86E-03	15
GO:0072659	protein localization to plasma membrane	0.14	1.56E-05	2.86E-03	15
GO:0016477	cell migration	0.12	1.72E-05	2.92E-03	15
GO:0006468	protein phosphorylation	0.07	5.74E-05	9.03E-03	15
GO:0007411	axon guidance	0.13	1.01E-04	1.48E-02	15
GO:0001764	neuron migration	0.13	1.15E-04	1.58E-02	15
GO:0030032	lamellipodium assembly	0.25	1.22E-04	1.58E-02	15
GO:0031581	hemidesmosome assembly	0.75	1.54E-04	1.69E-02	15
GO:0045669	positive regulation of osteoblast differentiation	0.17	1.45E-04	1.69E-02	15
GO:0048105	establishment of body hair planar orientation	0.75	1.54E-04	1.69E-02	15
GO:0035556	intracellular signal transduction	0.09	6.51E-08	1.25E-04	16
GO:0030154	cell differentiation	0.10	5.34E-07	3.43E-04	16
GO:0043406	positive regulation of MAP kinase activity	0.24	4.03E-07	3.43E-04	16
GO:0048008	platelet-derived growth factor receptor signaling pathway	0.28	2.90E-06	1.40E-03	16
GO:0001525	angiogenesis	0.12	6.58E-06	1.94E-03	16
GO:0050790	regulation of catalytic activity	0.07	5.98E-06	1.94E-03	16
GO:0055007	cardiac muscle cell differentiation	0.32	7.03E-06	1.94E-03	16
GO:0009190	cyclic nucleotide biosynthetic process	0.30	9.82E-06	2.37E-03	16
GO:0001666	response to hypoxia	0.15	1.20E-05	2.56E-03	16
GO:0008285	negative regulation of cell proliferation	0.08	1.53E-05	2.95E-03	16
GO:0070588	calcium ion transmembrane transport	0.12	3.29E-05	5.77E-03	16
GO:0007015	actin filament organization	0.14	4.15E-05	6.67E-03	16
GO:0030036	actin cytoskeleton organization	0.11	5.95E-05	8.39E-03	16
GO:0043547	positive regulation of GTPase activity	0.10	6.59E-05	8.39E-03	16
GO:0045636	positive regulation of melanocyte differentiation	0.75	7.40E-05	8.39E-03	16
GO:0055012	ventricular cardiac muscle cell differentiation	0.75	7.40E-05	8.39E-03	16
GO:1904753	negative regulation of vascular associated smooth muscle cell migration	0.75	7.40E-05	8.39E-03	16

GO:0006171	cAMP biosynthetic process	0.40	9.24E-05	8.91E-03	16
GO:0032060	bleb assembly	0.40	9.24E-05	8.91E-03	16
GO:0038063	collagen-activated tyrosine kinase receptor signaling pathway	0.40	9.24E-05	8.91E-03	16
GO:0016310	phosphorylation	0.07	3.60E-06	8.39E-03	17
GO:0030509	BMP signaling pathway	0.20	1.08E-05	1.26E-02	17
GO:0034446	substrate adhesion-dependent cell spreading	0.25	1.72E-05	1.33E-02	17
GO:0050790	regulation of catalytic activity	0.08	2.55E-05	1.49E-02	17
GO:0007179	transforming growth factor beta receptor signaling pathway	0.18	4.33E-05	2.02E-02	17
GO:0002062	chondrocyte differentiation	0.23	9.42E-05	2.45E-02	17
GO:0006468	protein phosphorylation	0.07	8.76E-05	2.45E-02	17
GO:0007015	actin filament organization	0.16	1.05E-04	2.45E-02	17
GO:0030308	negative regulation of cell growth	0.15	1.00E-04	2.45E-02	17
GO:0035335	peptidyl-tyrosine dephosphorylation	0.13	6.31E-05	2.45E-02	17
GO:0001649	osteoblast differentiation	0.17	1.58E-04	3.06E-02	17
GO:0043547	positive regulation of GTPase activity	0.11	1.57E-04	3.06E-02	17
GO:0006956	complement activation	0.43	2.46E-06	2.52E-03	18
GO:0007411	axon guidance	0.15	2.03E-06	2.52E-03	18
GO:0019221	cytokine-mediated signaling pathway	0.16	4.06E-06	2.77E-03	18
GO:0008285	negative regulation of cell proliferation	0.09	1.75E-05	5.98E-03	18
GO:0010811	positive regulation of cell-substrate adhesion	0.26	1.64E-05	5.98E-03	18
GO:0048738	cardiac muscle tissue development	0.45	1.27E-05	5.98E-03	18
GO:0010716	negative regulation of extracellular matrix disassembly	1.00	3.22E-05	7.34E-03	18
GO:0019218	regulation of steroid metabolic process	1.00	3.22E-05	7.34E-03	18
GO:0070487	monocyte aggregation	1.00	3.22E-05	7.34E-03	18
GO:0007605	sensory perception of sound	0.12	6.50E-05	1.33E-02	18
GO:0050919	negative chemotaxis	0.33	7.44E-05	1.39E-02	18
GO:0007507	heart development	0.10	9.03E-05	1.54E-02	18
GO:0045104	intermediate filament cytoskeleton organization	0.29	1.45E-04	2.29E-02	18

GO:0034332	adherens junction organization	0.40	1.84E-04	2.69E-02	18
GO:0035335	peptidyl-tyrosine dephosphorylation	0.11	2.59E-04	3.54E-02	18
GO:2000556	positive regulation of T-helper 1 cell cytokine production	0.60	3.07E-04	3.93E-02	18
GO:0030198	extracellular matrix organization	0.13	4.07E-04	4.91E-02	18
GO:0001525	angiogenesis	0.31	2.53E-19	7.12E-16	19
GO:0035556	intracellular signal transduction	0.15	4.83E-12	6.80E-09	19
GO:0006468	protein phosphorylation	0.12	8.07E-12	7.58E-09	19
GO:0016310	phosphorylation	0.10	4.35E-11	3.06E-08	19
GO:0045766	positive regulation of angiogenesis	0.24	6.55E-10	3.69E-07	19
GO:0001570	vasculogenesis	0.33	3.66E-09	1.29E-06	19
GO:0043547	positive regulation of GTPase activity	0.19	3.63E-09	1.29E-06	19
GO:0050790	regulation of catalytic activity	0.12	2.83E-09	1.29E-06	19
GO:0030334	regulation of cell migration	0.29	9.61E-09	3.01E-06	19
GO:0001938	positive regulation of endothelial cell proliferation	0.33	1.89E-08	5.32E-06	19
GO:0030036	actin cytoskeleton organization	0.20	2.41E-08	6.18E-06	19
GO:0030155	regulation of cell adhesion	0.37	5.70E-08	1.34E-05	19
GO:0007219	Notch signaling pathway	0.22	6.54E-08	1.42E-05	19
GO:0008285	negative regulation of cell proliferation	0.13	1.57E-07	3.16E-05	19
GO:0043536	positive regulation of blood vessel endothelial cell migration	0.36	3.09E-07	5.81E-05	19
GO:0030335	positive regulation of cell migration	0.15	4.89E-07	8.18E-05	19
GO:0045765	regulation of angiogenesis	0.39	4.94E-07	8.18E-05	19
GO:0016477	cell migration	0.16	1.02E-06	1.60E-04	19
GO:0001568	blood vessel development	0.28	1.57E-06	2.32E-04	19
GO:0007179	transforming growth factor beta receptor signaling pathway	0.23	1.83E-06	2.58E-04	19
GO:0007399	nervous system development	0.11	2.40E-06	3.39E-03	20
GO:0016311	dephosphorylation	0.08	8.93E-05	4.28E-02	20
GO:0035335	peptidyl-tyrosine dephosphorylation	0.10	9.09E-05	4.28E-02	20
GO:0051045	negative regulation of membrane protein ectodomain proteolysis	0.60	1.23E-04	4.36E-02	20

Table S15. Information on protein expression from proteomics sequencing of ovarian samples

Protein_ID	Description	Ratio	SD	Pvalue
A0A6P7EP28	complement factor H-like	4.73E-01	0.065	2.09E-07
A0A6P3TRN0	bcl-2-associated transcription factor 1 isoform X4	1.22E+00	0.043	2.85E-07
A0A835ZHN5	2-iminobutanoate/2-iminopropanoate deaminase	7.83E-01	0.035	2.99E-07
P68240	Hemoglobin subunit	5.71E-01	0.06	3.83E-07
W5Q0S2	Glutaminyl-tRNA synthetase	5.11E-01	0.071	7.47E-07
A0A6P3TJ18	Membrane cofactor protein	1.53E+00	0.141	1.14E-06
A0A6P3E5Q1	Aldehyde dehydrogenase (NAD(+))	4.94E-01	0.077	1.15E-06
W5PVY4	Laminin subunit alpha 2	7.67E-01	0.045	1.35E-06
W5Q6V2	Keratin 7	7.23E-01	0.054	1.70E-06
W5NTK8	NDRG family member 4	7.21E-01	0.056	2.13E-06
W5P7B7	Pappalysin 1	7.44E-01	0.052	2.20E-06
A0A836D7U8	Anoctamin	1.42E+00	0.124	3.43E-06
A0A835ZWU3	Threonyl-tRNA synthetase	1.20E+00	0.058	4.46E-06
A0A836AAA8	G protein gamma domain-containing protein	1.42E+00	0.128	4.63E-06
A0A6P3EH01	Choline-specific glycerophosphodiester phosphodiesterase	1.29E+00	0.085	4.89E-06
A0A836AD50	HMG box transcription factor 1	1.30E+00	0.093	6.59E-06
A0A836D225	Dihydrofolate reductase	1.21E+00	0.062	7.15E-06
W5QH60	Vesicle associated membrane protein 8	7.08E-01	0.07	7.37E-06
A0A6P3ECT1	apolipoprotein A-IV	8.02E-01	0.05	7.49E-06
W5P1G7	Heterogeneous nuclear ribonucleoprotein	1.27E+00	0.085	7.61E-06
A0A835ZL90	PHB domain-containing protein	1.27E+00	0.086	7.73E-06
W5PSI7	Angiopoietin like	1.26E+00	0.081	9.27E-06
A0A836CRU9	TLC domain-containing protein	1.25E+00	0.079	9.84E-06
A0A6P7ED26	Carbonic anhydrase	6.61E-01	0.08	1.13E-05
A0A6P7EGN9	exopolyphosphatase PRUNE1 isoform X2	1.29E+00	0.095	1.16E-05
W5NZR6	von Willebrand factor A domain containing	1.43E+00	0.149	1.30E-05

W5P336	Complement subcomponent C1r	8.24E-01	0.048	1.34E-05
A0A836AC75	Complement C3	8.28E-01	0.049	1.64E-05
W5PEJ5	Osteomodulin	7.15E-01	0.074	1.65E-05
A0A6P7EAI2	filamin-A-interacting protein 1 isoform X2	8.02E-01	0.055	1.69E-05
W5QHH2	Peroxisomal biogenesis factor 11 beta	1.55E+00	0.213	1.81E-05
W5P9X8	Nicotinamide-nucleotide adenylyltransferase	1.39E+00	0.139	2.19E-05
A0A835ZSQ7	NIPSNAP domain-containing protein	1.31E+00	0.112	2.22E-05
A0A6P3YQF9	Biglycan	8.13E-01	0.057	2.27E-05
W5PY57	RNA helicase	7.74E-01	0.067	2.36E-05
W5QDU4	Tetratricopeptide repeat protein 38	1.47E+00	0.181	2.44E-05
W5QBC8	Syndecan binding protein 2	1.35E+00	0.133	2.49E-05
W5P5C5	Glycogenin 1	1.31E+00	0.118	3.25E-05
A0A6P3TUQ5	disheveled-associated activator of morphogenesis 1 isoform X3	6.83E-01	0.092	3.75E-05
A0A6P7EJ41	CREB-regulated transcription coactivator 2 isoform X5	7.06E-01	0.09	4.02E-05
A0A6P7DE62	nuclear distribution protein nudE homolog 1 isoform X2	8.06E-01	0.062	4.19E-05
A0A6P7CZM4	SH3 domain-containing protein 19 isoform X2	7.83E-01	0.067	4.66E-05
A0A6P7DGC3	homeobox protein unc-4 homolog	1.29E+00	0.114	5.06E-05
W5PF30	Transmembrane and coiled-coil domains 3	1.33E+00	0.14	5.22E-05
W5PJB8	Glyco_tran_10_N domain-containing	8.32E-01	0.057	5.23E-05
A0A6P7DXN6	keratin, type I cytoskeletal 18	6.15E-01	0.119	5.69E-05
W5P8V7	Collapsin response mediator protein 1	1.40E+00	0.164	5.98E-05
A0A6P7EN50	mitofusin-2	1.21E+00	0.087	6.82E-05
A0A6P7DJS7	NHS-like protein 2	1.21E+00	0.086	7.38E-05
W5Q802	Mitochondrial ribosomal protein	7.98E-01	0.071	7.96E-05
A0A836A4C5	Signal recognition particle subunit SRP68	1.35E+00	0.156	8.14E-05
A0A6P7EV79	60S ribosomal protein L34	5.84E-01	0.135	8.20E-05
A0A6P3TNS9	Transmembrane 9 superfamily member	7.39E-01	0.086	8.27E-05
A0A6P7EUF9	protein Aster-B isoform X2	8.09E-01	0.067	8.95E-05

A0A6P3E7H1	ras-related protein Rab-2B	1.21E+00	0.091	9.49E-05
W5PI20	Perilipin 4	1.32E+00	0.14	9.59E-05
A0A836ACH5	Uncharacterized protein	6.20E-01	0.12	9.97E-05
A0A6P3TW05	60S ribosomal protein L13a	5.07E-01	0.15	9.99E-05
A0A6P7EVU4	EH domain-containing protein 2	7.92E-01	0.075	1.01E-04
A0A6P3YRY7	Cap-specific mRNA (nucleoside-2'-O-)-methyltransferase 1	1.24E+00	0.107	1.02E-04
W5PWQ9	Death associated protein	1.25E+00	0.106	1.03E-04
A0A835ZXR8	Coronin	7.95E-01	0.072	1.04E-04
W5P328	Eukaryotic translation initiation factor 2A	1.47E+00	0.217	1.11E-04
A0A6P7EGG9	keratin, type I cytoskeletal 19	7.07E-01	0.1	1.13E-04
A0A6P7EN84	la-related protein 4B isoform X2	7.63E-01	0.087	1.18E-04
W5P912	SEC24 homolog B, COPII coat complex component	8.02E-01	0.073	1.18E-04
A0A6P3E411	60S ribosomal protein L3	8.21E-01	0.066	1.29E-04
A0A836AAZ9	Uncharacterized protein	1.35E+00	0.158	1.29E-04
W5PMP7	CUB domain containing protein 1	2.07E+00	0.584	1.31E-04
A0A6P3EHN2	26S proteasome non-ATPase regulatory subunit 12	1.20E+00	0.092	1.34E-04
W5Q5N9	Keratin 8	5.30E-01	0.135	1.36E-04
W5PZH8	Membrane bound O-acyltransferase domain containing 2	1.22E+00	0.096	1.39E-04
A0A6P7EGR9	RNA helicase	1.21E+00	0.097	1.40E-04
A0A6P3EKU5	Protein lin-7 homolog	7.71E-01	0.087	1.40E-04
A0A6P3T2M4	Creatine kinase	7.91E-01	0.081	1.43E-04
A0A836AC50	Eukaryotic translation initiation factor 2 subunit 1	7.64E-01	0.09	1.47E-04
W5PH80	Chromosome alignment maintaining phosphoprotein 1	1.29E+00	0.139	1.52E-04
A0A6P3TAC5	60S ribosomal protein L35	4.04E-01	0.185	1.55E-04
A0A835ZY56	Proteasome subunit beta	1.28E+00	0.129	1.55E-04
A0A6P3TU07	ubiquitin-conjugating enzyme E2 J2	1.26E+00	0.12	1.64E-04
A0A6P7DC78	MMS19 nucleotide excision repair protein	1.43E+00	0.201	1.65E-04
A0A6P3TFN4	glomulin isoform X2	1.23E+00	0.109	1.68E-04

W5Q8U7	Torsin 1A interacting protein 1	1.47E+00	0.223	1.75E-04
W5NY95	C3/C5 convertase	7.95E-01	0.078	1.80E-04
A0A6P7E3J4	Perilipin	1.25E+00	0.126	1.88E-04
A0A835ZZH0	Uncharacterized protein	1.27E+00	0.133	1.92E-04
A0A836D034	ATP-binding cassette sub-family C member 7	7.35E-01	0.105	2.02E-04
A0A6P3YFK7	Down syndrome critical region protein 3 isoform X2	1.33E+00	0.157	2.03E-04
W5NRW4	Tropomyosin 4	6.99E-01	0.114	2.09E-04
A0A6P3E9K2	Calponin	6.15E-01	0.131	2.24E-04
A0A6P3E9B8	Platelet-activating factor acetylhydrolase	8.18E-01	0.073	2.27E-04
A0A6P7F1K3	erbin isoform X10	6.94E-01	0.116	2.31E-04
A0A6P3E4P2	heterogeneous nuclear ribonucleoprotein A/B isoform X2	1.22E+00	0.105	2.32E-04
W5PF04	alpha-1,2-Mannosidase	1.39E+00	0.2	2.33E-04
A0A6P7DXW5	HMG domain-containing protein 4 isoform X3	1.20E+00	0.098	2.36E-04
A0A6P3EJB2	Peptidylprolyl isomerase	7.69E-01	0.091	2.40E-04
A0A6P3YMH0	CKLF-like MARVEL transmembrane domain-containing protein 3	4.92E-01	0.175	2.49E-04
A0A835ZLR7	PABS domain-containing protein	4.77E-01	0.19	2.60E-04
W5Q6W0	DET1- and DDB1-associated protein 1	7.13E-01	0.113	2.62E-04
A0A6P3YLS9	solute carrier family 12 member 2 isoform X2	7.90E-01	0.089	2.63E-04
A0A6P3T8V3	caspase-13 isoform X3	1.28E+00	0.138	2.64E-04
A0A6P3YL58	60S ribosomal protein L18a	6.45E-01	0.141	2.65E-04
W5PQ22	Vacuolar protein sorting-associated protein 53 homolog	7.37E-01	0.107	2.68E-04
A0A6P7EUQ0	protein MRVI1 isoform X3	8.33E-01	0.073	2.84E-04
W5PG41	Hexose-6-phosphate dehydrogenase/glucose 1-dehydrogenase	1.23E+00	0.116	2.86E-04
A0A6P7EPY0	C-Maf-inducing protein isoform X2	1.43E+00	0.237	2.89E-04
A0A6P7EJB8	protein PRRC2C isoform X12	8.23E-01	0.077	2.92E-04
W5NQ68	SZT2 subunit of KICSTOR complex	1.27E+00	0.138	2.92E-04
A0A6P7EY29	brain acid soluble protein 1	7.92E-01	0.087	2.94E-04
W5QFQ0	Malate dehydrogenase	7.86E-01	0.089	2.95E-04

A0A6P7ETB1	Alpha-ketoglutarate-dependent dioxygenase FTO	6.81E-01	0.125	3.02E-04
A0A6P3T787	Protein YIPF	1.39E+00	0.197	3.07E-04
A0A6P3EDC9	non-histone chromosomal protein HMG-17	1.89E+00	0.554	3.10E-04
A0A6P3E5K6	G-rich sequence factor 1 isoform X2	1.25E+00	0.133	3.17E-04
A0A836A3E0	Glycylpeptide N-tetradecanoyltransferase	6.82E-01	0.125	3.23E-04
W5Q6N7	Thioredoxin domain containing	1.23E+00	0.114	3.32E-04
W5P9E8	PHD finger protein 14	1.27E+00	0.146	3.33E-04
A0A6P3ELN9	exosome complex component MTR3	1.22E+00	0.114	3.36E-04
W5NV61	Sorting nexin	6.68E-01	0.134	3.39E-04
W5PEK7	Formin like 2	1.21E+00	0.106	3.40E-04
A0A836CV59	Oxysterol-binding protein	1.30E+00	0.16	3.45E-04
A0A6P3TJ75	3-hydroxyisobutyryl-CoA hydrolase, mitochondrial	1.69E+00	0.397	3.53E-04
A0A835ZNY2	Serine/threonine-protein kinase RIO1	1.36E+00	0.19	3.54E-04
A0A6P3E0K4	asporin	5.28E-01	0.165	3.61E-04
W5P900	60S ribosomal protein L29	6.29E-01	0.151	3.79E-04
A0A6P7E3B9	ran-binding protein 3 isoform X4	1.22E+00	0.111	3.84E-04
A0A6P7D831	uncharacterized protein LOC101120961	7.48E-01	0.105	3.85E-04
A0A6P7EGU6	secernin-2 isoform X2	7.14E-01	0.124	4.09E-04
A0A6P3EBE7	transmembrane emp24 domain-containing protein 10	1.29E+00	0.159	4.12E-04
W5P710	General transcription factor IIIC subunit 4	1.43E+00	0.246	4.14E-04
A0A6P7EI32	hypermethylated in cancer 1 protein isoform X2	1.27E+00	0.143	4.14E-04
W5NPJ2	General transcription factor IIIC subunit 1	7.84E-01	0.097	4.18E-04
A0A6P3E242	U5 small nuclear ribonucleoprotein 40 kDa protein	1.37E+00	0.209	4.38E-04
A0A6P7EUV4	LOW QUALITY PROTEIN: tripartite motif-containing protein 5-like	8.26E-01	0.08	4.60E-04
W5Q2G1	Arachidonate--CoA ligase	1.20E+00	0.105	4.62E-04
W5Q905	BTB domain containing 11	7.84E-01	0.095	4.63E-04
A0A836A229	Hexosyltransferase	8.25E-01	0.081	4.65E-04
W5P3B0	Phosphatidylinositol-3,4,5-trisphosphate 5-phosphatase	7.99E-01	0.093	4.70E-04

I1WXR8	Alpha-1-antitrypsin transcript variant 3	5.72E-01	0.157	4.73E-04
C8BKE5	ELAV-like protein	1.26E+00	0.138	4.96E-04
A0A6P7DJE0	A-kinase anchor protein 4 isoform X1	6.52E-01	0.148	5.09E-04
A0A6P3TL35	Ribosomal protein L19	6.93E-01	0.129	5.11E-04
A0A6P7DWJ6	Adenylate cyclase	8.29E-01	0.081	5.22E-04
W5PD43	HtrA serine peptidase 1	7.40E-01	0.12	5.26E-04
A0A6P3TFC1	Protein kinase C	1.23E+00	0.121	5.26E-04
W5P382	Zinc binding alcohol dehydrogenase domain containing	1.27E+00	0.146	5.26E-04
Q1KYZ7	Beta-A globin chain	5.54E-01	0.16	5.63E-04
A0A6P7DUD4	Ribokinase	1.27E+00	0.153	5.76E-04
A0A6P7D2I7	two pore calcium channel protein 1 isoform X3	1.28E+00	0.156	6.09E-04
P14639	Albumin	8.26E-01	0.082	6.15E-04
W5QI00	Lactamase beta	1.26E+00	0.144	6.42E-04
A0A6P7DAT6	Tetraspanin	1.22E+00	0.119	6.45E-04
A0A6P7DXP1	LOW QUALITY PROTEIN: collagen alpha-3(VI) chain	8.00E-01	0.096	6.47E-04
A0A6P7F1D9	SH3 and PX domain-containing protein 2B	1.23E+00	0.131	6.49E-04
Q6ITZ6	Glycine amidinotransferase	1.35E+00	0.2	6.50E-04
A0A6P7E030	dedicator of cytokinesis protein 4 isoform X3	1.44E+00	0.261	6.57E-04
W5P6Q4	Death domain-containing	7.33E-01	0.118	6.57E-04
A0A6P7EAS1	sphingomyelin phosphodiesterase 2	1.33E+00	0.192	6.93E-04
A0A6P3YJB2	LOW QUALITY PROTEIN: zinc finger protein basonuclin-2	1.25E+00	0.139	7.06E-04
A0A6P7EQ87	liver carboxylesterase-like	1.63E+00	0.376	7.07E-04
W5QFF9	Complex I-15 kDa	8.15E-01	0.092	7.14E-04
A0A6P3T3X5	histone H1.5	4.42E-01	0.238	7.29E-04
A0A6P3TN39	Glucosamine-6-phosphate isomerase	1.21E+00	0.121	7.30E-04
A0A835ZRV9	CULLIN_2 domain-containing protein	1.32E+00	0.189	7.47E-04
A0A836A6Y4	Uncharacterized protein	7.26E-01	0.135	7.48E-04
A0A6P7EMS4	UPF0687 protein C20orf27 homolog	1.24E+00	0.133	7.49E-04

A0A6P3E5T8	60S ribosomal protein L4	6.30E-01	0.156	7.60E-04
A0A6P7EUN2	60S ribosomal protein L13	6.65E-01	0.146	7.66E-04
A0A6P3TV87	Cadherin-13	8.33E-01	0.086	7.74E-04
A0A836A4D6	DUF4211 domain-containing protein	7.30E-01	0.135	7.80E-04
A0A6P3ELJ0	transportin-1 isoform X2	1.26E+00	0.15	7.81E-04
A0A6P7E1H4	transmembrane protein 53	1.41E+00	0.259	7.91E-04
A0A6P7D1G5	Very-long-chain (3R)-3-hydroxyacyl-CoA dehydratase	1.48E+00	0.293	7.97E-04
W5P561	Actin related protein T2	7.62E-01	0.119	8.06E-04
A0A836A7C4	Antithrombin-III	7.62E-01	0.112	8.18E-04
A0A835ZTF3	Uncharacterized protein	8.22E-01	0.093	8.36E-04
W5PEY5	Protein-glucosylgalactosylhydroxylysine glucosidase	6.86E-01	0.144	8.37E-04
A0A6P7DDW5	protein disulfide-isomerase TMX3 isoform X1	8.05E-01	0.095	8.37E-04
W5Q0Y6	Ankyrin repeat domain 44	7.05E-01	0.143	8.43E-04
A0A6P7D3L4	laminin subunit beta-2 isoform X3	7.71E-01	0.116	8.49E-04
W5PQ38	Transportin 2	1.21E+00	0.127	8.82E-04
A0A836CXC3	t-SNARE coiled-coil homology domain-containing protein	7.30E-01	0.135	8.91E-04
A0A835ZMY9	MHC class I region proline-rich protein CAT53	1.31E+00	0.174	8.96E-04
A0A6P3EB14	Non-specific serine/threonine protein kinase	8.29E-01	0.088	9.11E-04
A0A6P7DHF2	Galectin	8.28E-01	0.091	9.21E-04
A0A836D477	Uncharacterized protein	1.27E+00	0.16	9.29E-04
W5P5W1	Integrin subunit alpha 3	7.70E-01	0.12	9.39E-04
W5NQD0	RNA binding motif protein 26	1.24E+00	0.14	9.40E-04
A0A6P3EJQ7	dnaJ homolog subfamily C member 11	1.24E+00	0.155	9.58E-04
W5P5J0	Drebrin 1	7.44E-01	0.134	9.61E-04
A0A6P3TEU5	Homeodomain-only protein	4.75E-01	0.25	9.66E-04
A0A6P3E922	syntaxin-17	7.97E-01	0.106	9.76E-04
W5P2Z4	Embryonic ectoderm development	7.41E-01	0.124	9.76E-04
A0A836D6T0	Exonuclease domain-containing protein	1.20E+00	0.124	9.78E-04

W5QEU2	Poly [ADP-ribose] polymerase	6.98E-01	0.151	9.94E-04
A0A6P3EB30	probable ribosome biogenesis protein RLP24	6.82E-01	0.151	1.05E-03
A0A6P7EDL7	Vacuolar protein sorting-associated protein 28 homolog	1.21E+00	0.127	1.08E-03
W5NRQ0	Tectonin beta-propeller repeat containing 1	7.86E-01	0.115	1.12E-03
A0A6P7ECF5	60S ribosomal protein L6	8.24E-01	0.096	1.12E-03
A0A6P7E5W9	Serine/threonine-protein kinase receptor	6.25E-01	0.175	1.13E-03
A0A6P3EAI8	Bax inhibitor 1	1.32E+00	0.191	1.14E-03
W5PV32	Piezo-type mechanosensitive ion channel component	1.20E+00	0.125	1.15E-03
W5PWK1	Gamma-aminobutyric acid type A receptor subunit alpha6	1.31E+00	0.196	1.16E-03
A0A6P3YKC6	Cell cycle control protein	1.25E+00	0.154	1.19E-03
A0A6P9FQX8	Guanine nucleotide-binding protein subunit beta-2-like 1	1.21E+00	0.123	1.19E-03
A0A6P7E7L5	negative elongation factor A	1.28E+00	0.171	1.20E-03
W5P8G3	3-ketodihydrosphingosine reductase	1.23E+00	0.14	1.20E-03
A0A6P7EVR4	RNA helicase	1.26E+00	0.161	1.21E-03
A0A6P3EK83	Ras-related protein Rab-18	7.80E-01	0.111	1.22E-03
A0A6P3E936	aldose reductase	1.23E+00	0.145	1.25E-03
W5PC91	Spectrin alpha chain, erythrocytic 1	7.91E-01	0.115	1.26E-03
W5Q8T1	Caseinolytic mitochondrial matrix peptidase chaperone subunit X	8.02E-01	0.105	1.28E-03
A0A836CSI1	Uncharacterized protein	8.16E-01	0.104	1.28E-03
W5QGM7	Histone H1.0	3.63E-01	0.243	1.29E-03
W5NSH2	Inter-alpha-trypsin inhibitor heavy chain 3	7.37E-01	0.145	1.39E-03
A0A836APB1	Secreted phosphoprotein 2	7.90E-01	0.119	1.41E-03
A0A6P3CX99	H/ACA ribonucleoprotein complex subunit 4	1.25E+00	0.152	1.43E-03
W5QFC8	Trophoblast	7.92E-01	0.11	1.44E-03
A0A6P7DAJ2	L-serine-phosphatidylethanolamine phosphatidyltransferase	1.53E+00	0.341	1.48E-03
A0A6P3YL25	serine incorporator 3	1.37E+00	0.249	1.48E-03
W5PVM6	Inter-alpha-trypsin inhibitor heavy chain 5	1.21E+00	0.129	1.50E-03
A0A6P7D278	Adenylate cyclase	1.26E+00	0.167	1.54E-03

A0A6P3T3Z3	histone H1.3	4.14E-01	0.307	1.57E-03
A0A836D1T7	Anti-Muellerian hormone	1.27E+00	0.168	1.58E-03
W5P1E2	Consortin, connexin sorting protein	1.25E+00	0.162	1.60E-03
A0A6P3CXP0	cytoglobin	1.38E+00	0.268	1.60E-03
W5Q0Y4	Fumarylacetoacetase	1.22E+00	0.139	1.63E-03
W5PZ14	Non-specific serine/threonine protein	1.29E+00	0.197	1.64E-03
A0A6P3THA8	Peptidylprolyl isomerase	1.22E+00	0.136	1.65E-03
A0A6P7E2H5	Vacuolar protein sorting-associated protein 41 homolog	1.28E+00	0.18	1.65E-03
A0A6P3ECC2	Glutaminyl-tRNA synthetase	1.61E+00	0.4	1.66E-03
W5PFY5	Ceramidase	1.36E+00	0.251	1.67E-03
A0A6P3EG68	60S ribosomal protein L8	6.41E-01	0.179	1.67E-03
A0A6P7ET62	40S ribosomal protein S9	7.92E-01	0.122	1.70E-03
A0A835ZY88	CSN12-like protein	8.14E-01	0.109	1.74E-03
A0A6P7EAM0	guanine nucleotide-binding protein subunit beta-5 isoform X1	8.04E-01	0.11	1.76E-03
A0A836ACJ6	Uncharacterized protein	1.36E+00	0.247	1.77E-03
W5P5F1	G protein-coupled receptor 108	8.29E-01	0.101	1.79E-03
W5P6M3	Family with sequence similarity 241 member B	1.77E+00	0.585	1.80E-03
A0A836CXL8	Splicing factor 45	7.53E-01	0.144	1.80E-03
A0A6P7DIH1	alpha-(1,3)-fucosyltransferase 10 isoform X2	1.45E+00	0.326	1.83E-03
Q30DR4	Macrophage migration inhibitory factor (Fragment)	7.76E-01	0.129	1.85E-03
W5QG72	Carbohydrate sulfotransferase	1.36E+00	0.245	1.88E-03
A0A6P3EG44	LOW QUALITY PROTEIN: serpin B4-like	6.63E-01	0.176	1.89E-03
A0A6P7D5G2	60S ribosomal protein L14	7.32E-01	0.144	1.90E-03
A0A6P7DY91	KICSTOR complex protein ITFG2	7.56E-01	0.132	1.91E-03
A0A6P3ERN3	Protein-serine/threonine kinase	1.22E+00	0.142	1.93E-03
A0A6P7DI35	kelch repeat and BTB domain-containing protein 11	6.29E-01	0.217	1.94E-03
A0A6P7EL27	Fibromodulin	5.10E-01	0.273	1.94E-03
W5P8H1	Asparagine--tRNA ligase	1.35E+00	0.251	1.95E-03

A0A6P3EEA2	immunoglobulin superfamily containing leucine-rich repeat protein	1.45E+00	0.315	2.03E-03
W5PHS4	Nucleus accumbens associated 1	7.72E-01	0.125	2.08E-03
W5NPR6	Apoptosis antagonizing transcription factor	1.34E+00	0.246	2.08E-03
W5NUN7	Tetraspanin	1.26E+00	0.174	2.09E-03
A0A6P7EU85	pleckstrin homology-like domain family B member 1 isoform X10	7.22E-01	0.165	2.09E-03
A0A6P7E8E5	RBR-type E3 ubiquitin transferase	7.99E-01	0.117	2.10E-03
W5PF53	ADP ribosylation factor like GTPase 5A	1.21E+00	0.138	2.11E-03
W5QJ02	Dehydrogenase/reductase 7	8.33E-01	0.099	2.12E-03
A0A6P7D0U1	merlin isoform X2	5.52E-01	0.262	2.12E-03
A0A6P3E581	leucine-rich repeat-containing protein 40	7.65E-01	0.137	2.13E-03
W5QAZ1	Coronin	1.23E+00	0.156	2.16E-03
A0A6P7EGN8	serine/threonine-protein kinase Nek3 isoform X6	1.63E+00	0.486	2.19E-03
A0A6P7EHM0	developmentally-regulated GTP-binding protein 2	6.84E-01	0.194	2.23E-03
A0A836A8Q9	DnaJ homolog subfamily C member 27	7.08E-01	0.157	2.28E-03
A0A836D4M4	60S ribosomal protein L7a	5.13E-01	0.267	2.29E-03
A0A6P7D290	zinc finger CCHC domain-containing protein 8 isoform X3	1.30E+00	0.202	2.30E-03
W5NU17	Uncharacterized	7.65E-01	0.14	2.30E-03
A0A6P7DX94	inhibitor of nuclear factor kappa-B kinase-interacting protein isoform X2	5.00E-01	0.283	2.33E-03
R4R2H5	Beta-casein	7.81E-01	0.137	2.33E-03
A0A6P7EK68	torsin-3A isoform X2	7.21E-01	0.156	2.36E-03
A0A835ZZ98	Epoxide hydratase	1.25E+00	0.175	2.37E-03
A0A6P7D5Q5	Glutathione S-transferase	7.15E-01	0.159	2.50E-03
W5QCB3	Solute carrier family 25 member 46	7.27E-01	0.155	2.50E-03
A0A6P7EKK6	cysteine and glycine-rich protein 1 isoform X2	6.41E-01	0.225	2.51E-03
A0A6P7EIE9	beta-arrestin-2 isoform X2	1.22E+00	0.151	2.51E-03
A0A6P7E936	zinc finger protein 280D isoform X4	1.29E+00	0.201	2.52E-03
A0A6P3EHB5	exosome complex component RRP41	7.74E-01	0.143	2.56E-03

A0A6P3T818	RING-type E3 ubiquitin transferase	1.55E+00	0.439	2.61E-03
W5QHG2	Pentatricopeptide repeat domain 3	1.41E+00	0.298	2.66E-03
A0A6P3E758	V-type proton ATPase subunit H	1.35E+00	0.248	2.66E-03
A0A6P3E812	dnaJ homolog subfamily B member 11	7.88E-01	0.129	2.68E-03
W5Q8F8	Coproporphyrinogen oxidase	1.43E+00	0.308	2.68E-03
W5NTA0	H1.2 linker histone, cluster	4.63E-01	0.335	2.74E-03
A0A6P3EA80	Transgelin	7.23E-01	0.18	2.77E-03
A0A6P7DAM2	delta(14)-sterol reductase isoform X3	1.23E+00	0.158	2.78E-03
W5PCV5	Ral GTPase activating protein catalytic subunit alpha 1	1.28E+00	0.209	2.82E-03
O78751	ATP synthase protein 8	1.26E+00	0.183	2.83E-03
P50413	Thioredoxin	6.43E-01	0.227	2.83E-03
A0A6P3DZ62	T-cell surface glycoprotein CD1e, membrane-associated	1.49E+00	0.379	2.84E-03
A0A6P3T974	glycophorin-C isoform X2	1.30E+00	0.21	2.85E-03
A0A6P7DS58	Neuroblastoma suppressor of tumorigenicity 1	7.83E-01	0.128	2.86E-03
A0A6P3ECY0	Beta-MPP	7.91E-01	0.133	2.86E-03
A0A6P3ECY3	acyl-coenzyme A thioesterase 13	1.50E+00	0.346	2.89E-03
A0A836CTH6	Uncharacterized protein	7.99E-01	0.128	2.93E-03
A0A6P3EAK6	mitoguardin 2	4.69E-01	0.322	2.96E-03
A0A835ZRX6	Complement component C9	7.35E-01	0.164	2.99E-03
W5PTS2	MAM domain containing 2	1.46E+00	0.335	2.99E-03
A0A6P7EK69	Protein-serine/threonine kinase	1.31E+00	0.218	3.05E-03
W5PZ55	C-X-C motif chemokine	7.00E-01	0.182	3.13E-03
A0A836AIN9	Epithelial cell adhesion molecule	6.43E-01	0.221	3.14E-03
A0A835ZS79	Transmembrane protein 231	1.22E+00	0.16	3.15E-03
A0A6P3E097	kininogen-1 isoform X1	8.31E-01	0.106	3.16E-03
A0A6P7E1V8	Calponin	7.02E-01	0.197	3.16E-03
A0A6P3ESE9	WD repeat domain phosphoinositide-interacting protein 4	1.21E+00	0.157	3.17E-03
A0A6P7DLX7	filamin-A isoform X4	8.22E-01	0.12	3.23E-03

A0A6P7EWG8	Phosphodiesterase	1.74E+00	0.563	3.33E-03
W5QBU3	Netrin G1	6.79E-01	0.182	3.38E-03
W5PIN0	Aldehyde dehydrogenase 1 family member A3	7.74E-01	0.153	3.43E-03
W5PXE1	Leucine rich repeat neuronal 1	1.35E+00	0.265	3.46E-03
W5PJW1	Succinate dehydrogenase cytochrome b560 subunit, mitochondrial	1.28E+00	0.202	3.57E-03
W5P050	Elongator complex protein 6	7.49E-01	0.152	3.60E-03
A0A6P7D8I8	Cathepsin D	1.30E+00	0.222	3.65E-03
W5QIU5	Glia maturation factor	1.24E+00	0.183	3.69E-03
A0A6P3T3Y7	histone H1.4	4.48E-01	0.382	3.82E-03
A0A6P3DYN5	Glutathione transferase	1.58E+00	0.481	3.82E-03
A0A6P3T825	LOW QUALITY PROTEIN: sushi, von Willebrand factor type A, EGF and pentraxin domain-containing protein 1	8.18E-01	0.117	3.85E-03
A0A6P3YIJ0	Receptor expression-enhancing protein	1.39E+00	0.315	3.95E-03
A0A6P7D337	Phosphatidylinositol-3-phosphate phosphatase	1.28E+00	0.215	3.95E-03
A0A6P7EHD5	leucine-rich repeat-containing protein 75A isoform X1	6.95E-01	0.18	4.02E-03
W5PLI1	THAP-type domain-containing	1.24E+00	0.175	4.05E-03
A0A6P3TCX5	m7GpppX diphosphatase	1.27E+00	0.202	4.14E-03
A0A836CRN2	SpaA domain-containing protein	1.25E+00	0.186	4.14E-03
A0A0P0KLF3	Cytochrome c oxidase subunit 2 (Fragment)	1.44E+00	0.33	4.19E-03
A0A6P3E422	plasminogen activator inhibitor 1 RNA-binding protein isoform X4	1.27E+00	0.215	4.26E-03
A0A6P7DWP4	periphilin-1 isoform X8	1.60E+00	0.487	4.26E-03
A0A6P3DZQ8	thrombospondin-3	8.01E-01	0.134	4.26E-03
W5P6X2	Insulin like growth factor binding protein 7	1.35E+00	0.259	4.27E-03
A0A6P3TKX2	krev interaction trapped protein 1	1.21E+00	0.163	4.30E-03
A0A835ZRV7	Lysyl oxidase homolog	8.12E-01	0.126	4.34E-03
A0A836ADS2	2-C-methyl-D-erythritol 4-phosphate cytidylyltransferase-like protein	1.36E+00	0.277	4.34E-03
A0A6P7DGL2	Major vault protein	1.22E+00	0.168	4.46E-03

W5Q7T8	Thrombospondin 4	7.62E-01	0.157	4.50E-03
A0A6P3T0Z2	inactive serine protease PAMR1 isoform X2	8.22E-01	0.118	4.51E-03
W5PLU1	Uncharacterized	7.26E-01	0.169	4.59E-03
A0A6P7DHC3	wings apart-like protein homolog isoform X2	1.22E+00	0.169	4.62E-03
W5Q8B1	Glutathione	6.75E-01	0.235	4.76E-03
W5PEJ0	Regulator of G protein signaling 19	4.69E-01	0.404	4.79E-03
A0A836AJV3	Receptor protein serine/threonine kinase	7.75E-01	0.158	4.80E-03
W5PXI3	Afamin	7.88E-01	0.143	4.80E-03
A0A6P7E366	ceramide synthase 4	1.45E+00	0.336	4.85E-03
W5PS64	G protein subunit alpha 14	1.22E+00	0.17	4.85E-03
A0A835ZZL2	SERPIN domain-containing protein	6.69E-01	0.217	4.87E-03
W5PTE9	Collagen type VI alpha 1 chain	7.72E-01	0.147	4.96E-03
A0A6P7DRY3	aflatoxin B1 aldehyde reductase member 2	1.32E+00	0.238	4.98E-03
A0A6P7DEA4	cytosolic purine 5'-nucleotidase isoform X6	1.26E+00	0.195	5.06E-03
A0A6P3EF14	TBC1 domain family member 25	7.90E-01	0.153	5.10E-03
W5NYW9	BHLH domain-containing	1.21E+00	0.167	5.13E-03
W5PZ18	SWI/SNF related, matrix associated, actin dependent regulator of chromatin, subfamily a, member 1	1.28E+00	0.221	5.15E-03
W5P1Z2	Spermidine synthase	1.20E+00	0.162	5.17E-03
A0A6P3YGV0	Ubiquitinyl hydrolase 1	7.60E-01	0.156	5.23E-03
A0A6P3E8G2	acyl-CoA dehydrogenase family member 11	8.14E-01	0.129	5.23E-03
A0A836A5X7	Uncharacterized protein	7.66E-01	0.157	5.28E-03
A0A6P3YPI0	hematopoietic progenitor cell antigen CD34 isoform X2	8.18E-01	0.13	5.31E-03
W5P333	Caspase recruitment domain family member 9	6.70E-01	0.208	5.32E-03
A0A6P3TMA6	programmed cell death protein 10	8.19E-01	0.126	5.38E-03
A0A6P7EB80	transforming growth factor beta receptor type 3 isoform X3	6.50E-01	0.209	5.48E-03
W5Q517	Procollagen C-endopeptidase enhancer	7.37E-01	0.182	5.53E-03
A0A6P7DZK5	Paraoxonase	7.53E-01	0.187	5.58E-03
A0A6P3CY24	1-Cys peroxiredoxin	1.33E+00	0.269	5.59E-03

A0A6P7EUE9	NLR family member X1 isoform X2	7.63E-01	0.158	5.69E-03
A0A6P3T7X6	serpin A3-7-like	6.25E-01	0.273	5.71E-03
A0A836AQC4	Uncharacterized protein	6.48E-01	0.245	5.76E-03
W5QF56	Condensin-2 complex subunit H2	1.29E+00	0.239	5.81E-03
A0A6P7EFL1	RING finger protein unkempt homolog isoform X2	7.81E-01	0.15	5.84E-03
W5PP26	Potassium channel tetramerization domain containing 2	2.00E+00	0.791	5.86E-03
A0A6P3E407	leucine-rich repeat-containing protein 17	8.00E-01	0.154	5.91E-03
W5P1I0	Lysine demethylase 6A	7.89E-01	0.162	5.92E-03
A0A836CWV2	HELP domain-containing protein	7.99E-01	0.14	5.95E-03
W5P7C8	Coiled-coil domain containing 178	1.43E+00	0.344	5.96E-03
A0A6P3EFU8	40S ribosomal protein S11	7.99E-01	0.139	6.02E-03
W5PW43	URB1 ribosome biogenesis homolog	1.22E+00	0.175	6.03E-03
A0A835ZLB6	Uncharacterized protein	8.02E-01	0.143	6.15E-03
A0A836AGQ4	Brix domain-containing protein	8.22E-01	0.133	6.23E-03
A0A836D6F9	Activin beta-A chain	6.00E-01	0.324	6.26E-03
A0A6P7D2B0	glutathione S-transferase theta-1 isoform X2	7.79E-01	0.151	6.26E-03
A0A6P3TDF7	Decorin	1.26E+00	0.212	6.27E-03
A0A6P3EJR0	PRP4 pre-mRNA-processing factor 4 homolog	1.36E+00	0.299	6.35E-03
A0A6P7DXX0	DNA replication licensing factor MCM5	8.26E-01	0.124	6.47E-03
W5NTE8	NSF attachment protein gamma	1.25E+00	0.202	6.50E-03
W5Q2Q1	WD repeat containing antisense to TP53	7.98E-01	0.148	6.55E-03
A0A6P7DMG1	ATP-binding cassette sub-family A member 1 isoform X3	1.46E+00	0.368	6.59E-03
A0A836CU08	Uncharacterized protein	7.50E-01	0.175	6.65E-03
A0A6P3E5B6	heparan sulfate 2-O-sulfotransferase 1 isoform X2	1.21E+00	0.166	6.67E-03
W5PW51	N-alpha-acetyltransferase 15, NatA auxiliary subunit	1.22E+00	0.182	6.68E-03
W5PXR1	Ectonucleotide pyrophosphatase/phosphodiesterase 1	1.30E+00	0.252	6.81E-03
W5P0D5	Proteasome 26S subunit, non-ATPase 7	1.23E+00	0.193	6.94E-03
W5QDY5	H(+)-transporting two-sector ATPase	6.73E-01	0.265	7.35E-03

W5PGJ4	Chimaerin	6.94E-01	0.225	7.41E-03
W5P1M4	Acetyl-CoA acyltransferase 2	1.30E+00	0.258	7.42E-03
A0A6P7D5E6	Phospholipid-transporting ATPase	7.72E-01	0.18	7.43E-03
B0LRQ2	Proteasome z subunit-like protein	1.58E+00	0.487	7.62E-03
A0A836CYM4	GDP-Man:Man(3)GlcNAc(2)-PP-Dol alpha-1,2-mannosyltransferase	1.20E+00	0.171	7.62E-03
A0A836AIV6	MPN domain-containing protein	7.46E-01	0.193	7.65E-03
W5Q6D2	Rabenosyn, RAB effector	1.22E+00	0.188	7.66E-03
W5PF65	Beta-1 metal-binding globulin	7.95E-01	0.158	7.68E-03
A0A6P3TRP8	Chloride intracellular channel protein	7.00E-01	0.251	7.73E-03
A0A6P3E9C5	60S ribosomal protein L36	7.54E-01	0.188	7.74E-03
W5P4P4	Fucose kinase	1.27E+00	0.233	7.83E-03
A0A6P7ESA0	protein kinase C-binding protein 1 isoform X14	1.27E+00	0.224	7.98E-03
P29701	Alpha-2-HS-glycoprotein	7.98E-01	0.151	7.98E-03
W5NUY7	Utrophin	7.90E-01	0.172	7.98E-03
A0A6P7DQP6	LOW QUALITY PROTEIN: dnaJ homolog subfamily B member 2	2.46E+00	1.292	8.02E-03
A0A835ZIA9	SEC7 domain-containing protein	1.61E+00	0.5	8.25E-03
A0A835ZQW8	Protein disulfide-isomerase	2.26E+00	1.092	8.27E-03
A0A6P3YPG0	Actin-depolymerizing factor	6.51E-01	0.238	8.27E-03
A0A6P3E4I9	basic leucine zipper and W2 domain-containing protein 2	7.81E-01	0.175	8.40E-03
A0A6P3YJE3	1-acyl-sn-glycerol-3-phosphate acyltransferase gamma	1.23E+00	0.184	8.44E-03
W5Q3C2	Epiplakin 1	7.44E-01	0.205	8.48E-03
A0A6P3YTT8	HIV Tat-specific factor 1	1.23E+00	0.192	8.58E-03
W5PFD5	PHD finger protein 11	1.88E+00	0.71	8.67E-03
A0A6P7E558	Pre-mRNA-splicing factor RBM22	1.53E+00	0.431	8.71E-03
A0A6P3YHL4	DNA-directed RNA polymerase I subunit RPA49	8.28E-01	0.13	8.71E-03
W5Q0Z6	Melanoma cell adhesion molecule	7.52E-01	0.207	8.81E-03
A0A6P7D4T8	eIF-2B GDP-GTP exchange factor subunit epsilon	1.58E+00	0.554	8.86E-03

A0A6P3EF83	Gc-globulin	7.82E-01	0.165	8.89E-03
A0A6P3E7U7	Mimecan	7.68E-01	0.195	9.06E-03
W5Q285	Programmed cell death 6	7.27E-01	0.192	9.14E-03
A0A6P3TY17	kinetochore-associated protein NSL1 homolog isoform X2	1.24E+00	0.211	9.28E-03
W5PEZ7	Cytochrome	1.33E+00	0.298	9.30E-03
A0A6P7DUV3	proto-oncogene c-Rel isoform X2	8.32E-01	0.137	9.39E-03
W5P2R7	Synapse defective Rho GTPase homolog 1	8.11E-01	0.15	9.41E-03
A0A6P3TCA3	Decapping nuclease	8.29E-01	0.136	9.41E-03
A0A6P7DKM7	uncharacterized protein LOC114111667	1.44E+00	0.41	9.60E-03
A0A6P3T9C7	dystrobrevin alpha isoform X18	7.08E-01	0.261	9.66E-03
W5PUP9	Complex III subunit 8	1.44E+00	0.417	9.76E-03
A0A6P3T603	complement C1q tumor necrosis factor-related protein 3 isoform X2	8.03E-01	0.152	9.77E-03
A0A6P7D9A0	Menin	6.64E-01	0.298	9.85E-03
A0A6P7D7V4	Phospholipase	8.20E-01	0.15	9.92E-03
A0A6P7DMP6	polypyrimidine tract-binding protein 3 isoform X4	1.34E+00	0.287	9.92E-03
W5PDT3	SEC24 homolog C, COPII coat complex component	1.38E+00	0.343	9.95E-03
W5PUI2	WD repeat domain 73	7.51E-01	0.21	1.00E-02
A0A835ZYU2	Uncharacterized protein	1.45E+00	0.365	1.00E-02
A0A6P3EKS3	twinkle protein, mitochondrial	1.44E+00	0.417	1.00E-02
A0A836D4X7	Lumican	8.33E-01	0.141	1.01E-02
W5QH50	Histidine rich glycoprotein	8.00E-01	0.154	1.02E-02
A0A6P7E426	PDZ and LIM domain protein 7 isoform X2	8.01E-01	0.169	1.02E-02
A0A6P7E5Y0	Protein-tyrosine-phosphatase	7.00E-01	0.238	1.02E-02
A0A6P7EA85	E3 ubiquitin-protein transferase MAEA	1.20E+00	0.173	1.02E-02
W5P2F6	Fas associated factor 1	1.29E+00	0.26	1.05E-02
W5Q297	Protein kinase C	7.93E-01	0.174	1.05E-02
A0A6P7ELZ6	hepatoma-derived growth factor isoform X1	1.25E+00	0.23	1.05E-02
A0A6P3CWZ0	MICOS complex subunit MIC19	1.72E+00	0.742	1.06E-02

W5PSQ7	Uncharacterized	7.67E-01	0.19	1.06E-02
A0A6P7D281	smoothelin isoform X4	6.13E-01	0.322	1.07E-02
W5P867	Non-specific serine/threonine protein kinase	8.31E-01	0.139	1.08E-02
A0A835ZW29	Haptoglobin	7.58E-01	0.218	1.09E-02
A0A6P7EQ05	Kelch-like protein 36	1.44E+00	0.39	1.10E-02
W5NWY8	Ankyrin repeat domain 49	8.24E-01	0.149	1.12E-02
A0A6P7DMH3	F-box/WD repeat-containing protein 12 isoform X3	1.21E+00	0.196	1.12E-02
A0A836A391	Alpha-1,6-mannosyl-glycoprotein 2-beta-N-acetylglucosaminyltransferase	8.21E-01	0.146	1.13E-02
A0A836A1L3	EF-hand domain-containing protein	1.24E+00	0.215	1.13E-02
A0A835ZMK0	Christmas factor	5.06E-01	0.283	1.14E-02
W5QJ74	SPARC related modular calcium binding 1	7.27E-01	0.251	1.15E-02
W5NTH9	DNA replication complex GINS protein PSF3	1.28E+00	0.248	1.16E-02
W5NRE6	Complex I subunit	1.69E+00	0.626	1.16E-02
A0A836AJD1	Uncharacterized protein	1.24E+00	0.227	1.16E-02
A0A836A9C0	Uncharacterized protein	7.86E-01	0.186	1.20E-02
A0A6P7E8Q6	mitogen-activated protein kinase-binding protein 1 isoform X3	8.19E-01	0.16	1.21E-02
A0A6P3TF53	tropomyosin alpha-1 chain isoform X10	7.81E-01	0.201	1.22E-02
A0A6P7EIQ4	vacuolar protein sorting-associated protein 45 isoform X2	1.34E+00	0.332	1.26E-02
A0A6P3TMF6	5'-3' exoribonuclease	1.23E+00	0.204	1.26E-02
A0A6P7EGL6	nucleotide triphosphate diphosphatase NUDT15 isoform X1	1.37E+00	0.331	1.29E-02
A0A679IU55	MHC class II DRB (Fragment)	8.22E-01	0.159	1.30E-02
A0A835ZJL3	V-type proton ATPase proteolipid subunit	1.73E+00	0.619	1.30E-02
A0A836AII1	Uncharacterized protein	7.58E-01	0.204	1.31E-02
A0A836A2M3	Butyryl-CoA dehydrogenase	1.38E+00	0.339	1.31E-02
A0A835ZZH1	Gastrin domain-containing protein	1.41E+00	0.36	1.33E-02
A0A6P3ERW4	ribosomal biogenesis protein LAS1L isoform X3	1.25E+00	0.228	1.34E-02
A0A6P7DJU5	dedicator of cytokinesis protein 11 isoform X3	7.39E-01	0.253	1.34E-02
A0A6P3EIU6	keratin, type I microfibrillar 48 kDa, component 8C-1	7.32E-01	0.236	1.37E-02

W5Q7U7	Serine/threonine-protein phosphatase	8.28E-01	0.16	1.37E-02
A0A6P7DZI6	EF-hand calcium-binding domain-containing protein 4B	1.21E+00	0.2	1.38E-02
A0A6P3YK36	angiogenin-2-like	1.22E+00	0.213	1.39E-02
A0A6P3EBB9	influenza virus NS1A-binding protein	6.78E-01	0.244	1.39E-02
A0A6P3YID4	SLAIN motif-containing protein 2 isoform X2	8.00E-01	0.175	1.40E-02
A0A835ZLM5	Angiotensinogen	8.33E-01	0.14	1.40E-02
A0A6P7EM38	Neuropilin	1.27E+00	0.236	1.41E-02
W5NUA0	Proline and arginine rich end leucine rich repeat protein	7.34E-01	0.252	1.42E-02
A0A6P3TJM2	SPATS2-like protein isoform X1	1.34E+00	0.307	1.48E-02
A0A6P3TWW7	dehydrogenase/reductase SDR family member 7B isoform X3	7.60E-01	0.195	1.48E-02
A0A6P7DBV9	sorbin and SH3 domain-containing protein 1 isoform X23	7.69E-01	0.226	1.54E-02
A0A6P3TJN4	ketimine reductase mu-crystallin isoform X2	6.68E-01	0.256	1.55E-02
A0A6P7E317	Transmembrane protein 205	1.24E+00	0.224	1.55E-02
A0A6P3E1J4	splicing factor 3B subunit 1 isoform X1	1.24E+00	0.237	1.58E-02
W5PW09	RING-type E3 ubiquitin transferase	1.24E+00	0.232	1.59E-02
W5NYL2	Caspase-1-like	1.23E+00	0.225	1.60E-02
A0A6P7E566	Mediator of RNA polymerase II transcription subunit 7	1.20E+00	0.189	1.60E-02
A0A6P7DBG5	Metalloendopeptidase	1.21E+00	0.194	1.61E-02
A0A6P9FQK3	Tumor necrosis factor receptor 1	8.23E-01	0.167	1.63E-02
A0A6P7EKM8	nuclear receptor corepressor 1 isoform X16	1.21E+00	0.205	1.64E-02
A0A6P3YFZ8	vitamin K-dependent protein C	1.37E+00	0.363	1.64E-02
A0A835ZZ02	Uncharacterized protein	6.99E-01	0.235	1.65E-02
A0A836CTJ5	LIM zinc-binding domain-containing protein	7.17E-01	0.298	1.69E-02
A0A6P3E8A9	Glycine cleavage system P protein	8.09E-01	0.173	1.73E-02
A0A6P3EBB2	laminin subunit gamma-1	8.31E-01	0.161	1.73E-02
G0UE67	MHC class II antigen (Fragment)	2.23E+00	1.411	1.74E-02
A0A835ZJQ4	Peptidase S1 domain-containing protein	7.50E-01	0.253	1.75E-02
A0A6P3E8I8	Tetratricopeptide repeat protein 30	7.44E-01	0.217	1.75E-02

W5Q233	Versican	1.23E+00	0.236	1.75E-02
W5P1B7	Hexosyltransferase	7.41E-01	0.218	1.76E-02
A0A6P3EJ13	Coiled-coil domain-containing protein 167	7.52E-01	0.226	1.78E-02
A0A6P7EGX7	ABI gene family member 3	8.09E-01	0.171	1.78E-02
W5PQV8	Protein phosphatase 1 regulatory subunit 18	8.26E-01	0.157	1.78E-02
A0A836D6R2	RECA_2 domain-containing protein	7.19E-01	0.261	1.80E-02
W5P259	Nuclear factor of activated T cells	1.21E+00	0.197	1.80E-02
A0A6P9FR04	Inhibin alpha chain	7.17E-01	0.307	1.82E-02
A0A836CTG3	Uncharacterized protein	8.02E-01	0.176	1.83E-02
A0A6P3YK05	GRB2-related adapter protein isoform X2	7.83E-01	0.22	1.84E-02
A0A836D2F2	Uncharacterized protein	1.23E+00	0.229	1.84E-02
A0A6P3E821	Dihydrolipoyllysine-residue succinyltransferase component of 2-oxoglutarate dehydrogenase complex, mitochondrial	1.30E+00	0.294	1.88E-02
A0A6P3EA70	testis-specific serine/threonine-protein kinase 3	7.28E-01	0.284	1.88E-02
W5PZ03	Sorting nexin 16	2.26E+00	1.468	1.89E-02
W5PZI8	Interferon induced protein with tetratricopeptide repeats	1.31E+00	0.314	1.89E-02
A0A835ZMT9	Vitamin-K-epoxide reductase (warfarin-sensitive)	1.46E+00	0.481	1.94E-02
A0A835ZPN7	NADH dehydrogenase [ubiquinone] flavoprotein 1, mitochondrial	1.32E+00	0.311	1.95E-02
A0A6P7EH88	GA-binding protein subunit beta-2 isoform X3	7.96E-01	0.199	1.96E-02
A0A835ZII2	Uncharacterized protein	8.30E-01	0.171	1.99E-02
W5QHJ3	Atlastin GTPase 1	1.22E+00	0.208	2.03E-02
A0A6P3EEG7	centrin-3	1.26E+00	0.274	2.07E-02
A0A6P3TNI7	Heterochromatin protein 1-binding protein 3	8.26E-01	0.18	2.08E-02
A0A6P3EPM7	histone H1x	8.31E-01	0.179	2.08E-02
W5PP85	TBC1 domain family member 10C	7.83E-01	0.194	2.09E-02
A0A6P7E008	synaptophysin-like protein 1	1.26E+00	0.264	2.12E-02
W5PJ41	DLC1 Rho GTPase activating protein	6.35E-01	0.285	2.15E-02
A0A6P3YMT5	Beta-galactosidase	1.25E+00	0.254	2.18E-02
A0A836A4R9	Uncharacterized protein	7.71E-01	0.252	2.22E-02

W5PUF6	Laminin subunit beta 3	1.22E+00	0.227	2.27E-02
Q7M371	Plasma proteinase inhibitor	8.19E-01	0.191	2.33E-02
A0A6P3E7G9	Apolipoprotein A-II	7.23E-01	0.324	2.33E-02
W5NSB1	Protein-serine/threonine kinase	8.06E-01	0.21	2.33E-02
A0A6P3TJ52	serine/arginine-rich splicing factor 7 isoform X3	1.29E+00	0.319	2.35E-02
W5PVQ4	Transmembrane protein 120A	1.46E+00	0.437	2.37E-02
A0A6P3T4B6	Hermansky-Pudlak syndrome 5 protein homolog	1.32E+00	0.353	2.39E-02
W5PHE9	Melanoma-associated antigen D4	8.22E-01	0.176	2.40E-02
A0A6P3TY70	kinesin-like protein KIF16B isoform X4	1.23E+00	0.229	2.40E-02
A0A0R5Z2R2	Cysteine and glycine-rich protein 2	7.44E-01	0.302	2.44E-02
W5PG82	WAS/WASL interacting protein family member 3	1.78E+00	0.914	2.47E-02
A0A836CTX9	Ig-like domain-containing protein	7.13E-01	0.276	2.48E-02
A0A6P3TWD0	Eukaryotic translation initiation factor 5A	1.22E+00	0.22	2.52E-02
A0A6P7DYQ1	NADH dehydrogenase [ubiquinone] 1 alpha subcomplex subunit 6	1.50E+00	0.571	2.56E-02
A0A6P3TDX5	Microtubule-associated protein RP/EB family member 2	7.20E-01	0.351	2.62E-02
W5P6D5	BPTI/Kunitz inhibitor domain-containing	8.29E-01	0.188	2.62E-02
A0A6P3TI71	Very-long-chain (3R)-3-hydroxyacyl-CoA dehydratase	1.38E+00	0.396	2.63E-02
A0A836D558	Uncharacterized protein	1.20E+00	0.221	2.65E-02
W5P374	Ribosomal protein	7.82E-01	0.241	2.65E-02
A0A6P3E3Z3	transmembrane glycoprotein NMB	3.60E+00	2.97	2.66E-02
A0A6P7DJR1	apoptosis-inducing factor 2 isoform X4	2.57E+00	1.925	2.68E-02
A0A6P3EIE1	complement factor H-related protein 2	7.13E-01	0.264	2.69E-02
A0A6P3EC56	Lon protease homolog 2, peroxisomal	1.24E+00	0.256	2.70E-02
W5Q6F1	Starch binding domain	7.97E-01	0.227	2.75E-02
W5P473	Cytochrome c oxidase polypeptide	1.33E+00	0.371	2.75E-02
A0A835ZWH6	Amine oxidase	8.18E-01	0.188	2.77E-02
A0A6P7D9Q7	mitochondrial carrier homolog 1 isoform X2	1.22E+00	0.221	2.80E-02
A0A6P7DTF6	sarcosine dehydrogenase, mitochondrial	8.16E-01	0.201	2.83E-02

A0A836A6W2	Methyltransf_11 domain-containing protein	1.22E+00	0.237	2.92E-02
A0A6P7DBU0	5'-3' exoribonuclease	1.35E+00	0.338	2.92E-02
W5Q497	Geranylgeranyl transferase type I subunit beta	1.25E+00	0.269	2.93E-02
W5PID7	Ubiquitination factor E4A	8.02E-01	0.24	2.94E-02
A0A836CUT2	SRCR domain-containing protein	1.20E+00	0.219	2.96E-02
A0A6P7D3U0	neurobeachin-like protein 2 isoform X3	1.21E+00	0.213	2.96E-02
W5PQL7	Tropomyosin 2	8.30E-01	0.185	2.99E-02
W5Q098	Homeobox containing 1	1.25E+00	0.268	3.05E-02
W5P0G4	Zinc finger and BTB domain containing 47	6.53E-01	0.382	3.07E-02
A0A6P7D3X4	Creatine kinase	7.13E-01	0.4	3.07E-02
Q9GJY3	Sulfate transporter	1.27E+00	0.288	3.18E-02
W5Q8P7	Uncharacterized	1.23E+00	0.265	3.18E-02
A0A6P7DMG3	protein NipSnap homolog 3A	1.26E+00	0.266	3.19E-02
A0A6P3TAC1	heat shock 70 kDa protein 4L	1.38E+00	0.414	3.21E-02
A0A835ZZW7	Prothrombin	8.21E-01	0.199	3.25E-02
Q9XT27	Ceruloplasmin	8.05E-01	0.204	3.25E-02
A0A6P3TAC4	Electron transfer flavoprotein-ubiquinone oxidoreductase	1.38E+00	0.448	3.30E-02
W5PW89	Rho GTPase activating protein 18	7.72E-01	0.276	3.33E-02
W5PPX8	Sortilin related VPS10 domain containing receptor 2	7.94E-01	0.261	3.33E-02
W5Q878	Protein kinase C	7.90E-01	0.264	3.34E-02
B0LRN3	Histone H3	7.97E-01	0.261	3.40E-02
W5PE46	Mitochondrial ribosomal protein L48	1.52E+00	0.546	3.49E-02
W5PY85	Single-pass membrane and coiled-coil domain-containing protein	1.21E+00	0.227	3.56E-02
A0A836CR40	DNA polymerase	1.25E+00	0.289	3.56E-02
A0A836CU14	Uncharacterized protein	7.95E-01	0.228	3.60E-02
W5NVF2	Uncharacterized	8.19E-01	0.195	3.65E-02
A0A6P3EDV5	UPF0462 protein C4orf33 homolog	8.08E-01	0.235	3.67E-02
W5QBE4	Fibrinogen like 2	7.75E-01	0.305	3.73E-02

A0A6P7DMR8	Tyrosine-protein kinase	1.39E+00	0.458	3.75E-02
W5P794	Rho guanine nucleotide exchange factor 25	8.32E-01	0.182	3.76E-02
A0A6P3ECA8	RNA-binding protein 5	1.22E+00	0.262	3.77E-02
A0A6P3TU23	acidic leucine-rich nuclear phosphoprotein 32 family member A isoform X2	1.42E+00	0.49	3.80E-02
W5QGV5	Dedicator of cytokinesis 10	7.96E-01	0.22	3.85E-02
W5PUB7	Spermatogenesis associated	1.30E+00	0.327	3.85E-02
A0A6P7DWR8	Glypican-1	1.23E+00	0.244	3.87E-02
A0A6P3EC26	Cytochrome b-c1 complex subunit Rieske, mitochondrial	1.35E+00	0.41	3.88E-02
A0A836DA11	Myelin peripheral protein	8.21E-01	0.208	3.88E-02
A0A6P7ESV3	Cytoplasmic tRNA 2-thiolation protein 1	5.99E-01	0.357	3.89E-02
A0A6P3TD93	GTPase IMAP family member 5-like	7.47E-01	0.33	3.92E-02
A0A6P3TF12	protein arginine N-methyltransferase 3 isoform X2	7.39E-01	0.361	3.94E-02
W5QI99	E3 ubiquitin-protein ligase	1.22E+00	0.251	4.01E-02
E1CEX6	Vomeronasal type-1 receptor (Fragment)	1.30E+00	0.347	4.02E-02
W5NTX1	Uncharacterized	7.07E-01	0.687	4.04E-02
A0A6P7DWK8	sentrin-specific protease 1 isoform X2	7.76E-01	0.24	4.06E-02
A0A6P7EFM0	Acyl-coenzyme A oxidase	1.37E+00	0.414	4.06E-02
W5PVR9	Endoplasmic reticulum metallopeptidase 1	1.21E+00	0.239	4.11E-02
A0A6P7EFB5	LOW QUALITY PROTEIN: fas-binding factor 1	8.24E-01	0.214	4.12E-02
A0A6P7DWZ3	sideroflexin-5 isoform X2	8.10E-01	0.23	4.13E-02
A0A6P3TMC3	pre-mRNA 3' end processing protein WDR33 isoform X4	1.33E+00	0.361	4.28E-02
W5PGT1	Nucleoporin 188	7.98E-01	0.272	4.38E-02
W5PBM9	Carboxypeptidase	3.58E+00	2.708	4.39E-02
W5Q8U3	Chordin like 1	1.47E+00	0.561	4.39E-02
A0A6P7E9S4	LOW QUALITY PROTEIN: latent-transforming growth factor beta-binding protein 2	8.01E-01	0.275	4.51E-02
W5NVR9	Chromosome 21 C11orf54 homolog	8.13E-01	0.242	4.51E-02
A0A836AAW8	Calcitonin gene-related peptide type 1 receptor	8.00E-01	0.258	4.64E-02

W5PP47	Cystatin domain-containing	1.31E+00	0.369	4.67E-02
A0A6P7D1Y5	iron-sulfur cluster co-chaperone protein HscB, mitochondrial isoform X2	8.22E-01	0.251	4.88E-02
A0A6P7DWC3	40S ribosomal protein SA-like	1.25E+00	0.319	4.91E-02

Table S16. Statistics of hormone levels in serum of different groups

Samples	E_2(ng/L)	P_4(ng/mL)	LH(UI/L)	T(pg/mL)	FSH(IU/L)
LF1	10.62	1.29	4.74	279.99	2.61
LF2	9.82	1.33	5.52	340.49	2.63
LF3	9.06	1.43	5.08	297.63	2.75
LF4	10.22	1.33	4.49	302.67	2.55
LF5	9.60	1.25	5.31	325.36	2.69
LF6	9.82	1.39	4.90	312.76	2.83
HF1	11.82	1.69	6.30	359.39	3.64
HF2	12.72	1.48	6.06	399.72	2.95
HF3	10.51	1.79	6.62	356.87	3.26
HF4	12.22	1.73	6.05	350.57	3.84
HF5	12.07	1.44	5.81	384.60	3.00
HF6	10.37	1.74	6.30	372.00	3.09

Table S17. The information of the KASP primers for the *PAPPA* gene and *BMPR1B* gene

Gene	Primer Name	Primer sequence
PAPPA	PAPPA-Primer1	GAAGGTGACCAAGTTCATGCTTGAATTTGAACATTTCTAGGAGGCAA
	PAPPA-Primer2	GAAGGTCGGAGTCAACGGATTTGAATTTGAACATTTCTAGGAGGCAG
	PAPPA-Common	AAACACTTTAAGGCTCTGAATGGC
BMPR1B	BMPR1B-Primer1	GAAGGTGACCAAGTTCATGCTTTTCATGCCTCATCAACACCGTCT
	BMPR1B-Primer2	GAAGGTCGGAGTCAACGGATTCATGCCTCATCAACACCGTCC
	BMPR1B-Common	CAGCTGGTTCCGAGAGACAGAAATA

SI References

Pei J, Xiong L, Guo S, Wang X, Bao P, Wu X, Yan P, Guo X. 2023. A single-cell transcriptomic atlas characterizes cell types and their molecular features in yak ovarian cortex. *FASEB J.* 37: e22718.

Wagner M, Yoshihara M, Douagi I, Damdimopoulos A, Panula S, Petropoulos S, Lu H, Pettersson K, Palm K, Katayama S, et al. 2020. Single-cell analysis of human ovarian cortex identifies distinct cell populations but no oogonial stem cells. *Nat Commun.* 11:1147.

Wang S, Zheng YX, Li JY, Yu Y, Zhang WQ, Song MS, Liu ZP, Min ZY, Hu HF, Jing Y, et al. 2020. Single-Cell Transcriptomic Atlas of Primate Ovarian Aging. *Cell* 180:585-600.

Fan X, Bialecka M, Moustakas I, Lam E, Torrens-Juaneda V, Borggreven NV, Trouw L, Louwe LA, Pilgram GSK, Mei H, et al. 2019. Single-cell reconstruction of follicular remodeling in the human adult ovary. *Nat Commun.* 10:3164.

Sheng X, Zhou J, Kang N, Liu W, Yu L, Zhang Z, Zhang Y, Yue Q, Yang Q, Zhang X, et al. 2022. Temporal and spatial dynamics mapping reveals follicle development regulated by different stromal cell populations. Developmental Biology Available from: http://biorxiv.org/lookup/doi/10.1101/2022.03.04.480328

Wang J-J, Tian Y, Li M-H, Feng Y-Q, Kong L, Zhang F-L, Shen W. 2021. Single-cell transcriptome dissection of the toxic impact of Di (2-ethylhexyl) phthalate on primordial follicle assembly. *Theranostics* 11:4992–5009.

Zhao Z-H, Li C-Y, Meng T-G, Wang Y, Liu W-B, Li A, Cai Y-J, Hou Y, Schatten H, Wang Z-B, et al. 2020. Single-cell RNA sequencing reveals regulation of fetal ovary development in the monkey (*Macaca fascicularis*). *Cell Discov*. 6:97.
